# The genetic architecture of cortical similarity networks

**DOI:** 10.1038/s41467-026-73714-9

**Published:** 2026-06-20

**Authors:** Isaac Sebenius, Varun Warrier, Richard A. I. Bethlehem, Richard Dear, Eva-Maria Stauffer, Yuanjun Gu, Rafael Romero-Garcia, Jakob Seidlitz, Edward Bullmore, Sarah Morgan

**Affiliations:** 1 Department of Psychiatry, University of Cambridge, Cambridge, United Kingdom; 2 Department of Computer Science and Technology, University of Cambridge, Cambridge, United Kingdom; 3 Harvard Medical School, Boston, MA US; 4 Department of Psychology, University of Cambridge, Cambridge, United Kingdom; 5 Autism Research Centre, University of Cambridge, Cambridge, United Kingdom; 6 Instituto de Biomedicina de Sevilla (IBiS) HUVR/CSIC/Universidad de Sevilla/CIBERSAM, ISCIII, Departamento de Fisiología Médica y Biofísica, Seville, Spain; 7 Department of Psychiatry, University of Pennsylvania, Philadelphia, PA USA; 8 Department of Child and Adolescent Psychiatry and Behavioral Science, The Children’s Hospital of Philadelphia, Philadelphia, PA USA; 9 Lifespan Brain Institute, The Children’s Hospital of Philadelphia, Philadelphia, PA USA; 10 School of Academic Psychiatry, Institute of Psychiatry, Psychology & Neuroscience, King’s College London, London, United Kingdom; 11 School of Biomedical Engineering & Imaging Sciences, King’s College London, London, United Kingdom

**Keywords:** Genetics of the nervous system, Neuroscience

## Abstract

The genetic architecture of human brain networks is central to understanding cortical organisation and evolution, the causal links between brain structure and function, and the pathogenesis of neuropsychiatric disorders. Using N > 48,000 subjects, we investigated common genetic effects on Morphometric INverse Divergence (MIND), a heritable, multi-modal structural MRI metric of inter-areal similarity and connectivity. Genetic correlations between MIND network edges were largely reducible to two gradients, each aligned with distance from one of the two phylogenetically primitive areas (paleocortex and archicortex) predicted by the dual origin theory of cortical evolution. MIND was more heritable than comparable measures of functional (f)MRI connectivity, and the paleocortically-aligned MIND gradient was genetically correlated with, and causally predictive of, fMRI connectivity. Finally, we identified genetic overlaps between MIND gradients and neuropsychiatric and biomedical traits. These results provide fresh insight into the dual origins of the cortex and their implications for brain function and health.

## Abstract (web summary)

Brain networks are heritable, but their genetics remain incompletely understood. Here, authors show that the genetics of cortical similarity networks relate to cortical evolution, causally predict functional connectivity, and link to psychiatric risk.

## Introduction

Identifying the genetic basis of the human brain’s complex network structure^1,2^ is essential for understanding how the brain develops, functions, and relates to health and cognition^3^. Over a decade of work, rooted in seminal twin studies, has shown that individual differences in brain network organisation measured by multiple modalities of magnetic resonance imaging (MRI) are under genetic influence^3–6^. Recent genome-wide association studies (GWAS) have since enabled the investigation of common genetic variation associated with certain brain network phenotypes, including (f)MRI-derived functional connectivity (FC) from correlational analysis of regional time-series, and diffusion (d)MRI-derived structural connectivity (SC) from tractographic reconstruction of axonal projections between regions^7–11^. However, our understanding of genetic influences on brain network organisation remains fragmented. For example, it has proven difficult to identify any meaningful genetic overlap between brain structural and functional connectivity phenotypes derived from different MRI modalities^8,9,12,13^. The spatially varying genetic architecture of localised (regional) MRI measures of brain structure has been well characterised by both twin studies^14,15^ and more contemporary GWAS^16–22^. It has also been shown by both twin studies^14^ and GWAS^20^ that common genetic effects are largely distributed across major systems or classes of cortical areas, implying that formation of large-scale cortical gradients (of lamination) or cortical networks (of axonal or synaptic connectivity) are under genetic control. However, it has not yet been shown how such genetically-driven cortical gradients derived from structural MRI data can be related to prior theories of cortical organisation, or measures of functional connectivity, or genetic risks for neuropsychiatric disorders and biomedical traits.

To address these key knowledge gaps, we used an innovative approach to human MRI network phenotyping, based on estimating the structural similarity between cortical areas. It has already been established that the similarity between cortical areas, which is measurable at the whole-brain scale of MRI, e.g., in terms of inter-areal correlation of macro- and/or micro-structural MRI feature vectors, is representative of the architectonic similarity between areas at a cellular scale^23–26^. Architectonically similar areas of cortex are also more likely to be inter-connected by axonal projections^27–29^. As such, structural similarity is well positioned to bridge structural MRI features defined locally (e.g., cortical thickness) with network-based measures that are defined relationally between brain areas (e.g., functional connectivity).

Several methods have emerged recently to estimate the connectome from MRI similarity analysis^24,25,30,31^. We used Morphometric INverse Divergence (MIND) for several technical, biological, and practical reasons. First, it has been biologically validated and technically benchmarked favourably against alternative structural connectivity and similarity estimators^25^ Importantly, MIND similarity was strongly co-located with cortical patterns of genomic co-expression, and has moderate twin-based heritability (average nodal ${h}_{twin}^{2} \sim 0.15$), indicating that MIND phenotypes might be advantageous for analysis of genetic effects on brain networks^25^. Finally, since its recent development, MIND has been rapidly taken up by the research community for the analysis of brain network organisation across many different applications, including lifespan development^32^, neuropsychiatric and neurodegenerative disorders^33–37^, cross-species connectomics^38,39^, and beyond. As such, a comprehensive genetic analysis of MIND network phenotypes is not only empirically and theoretically justified, but also timely.

Here, we used multimodal MRI from *N* > 30,000 subjects (and replicated in an additional *N* > 18,000) from the UK Biobank (UKB) to estimate GWAS statistics independently for each edge of the MIND structural similarity network. This preliminary analysis demonstrated that MIND network edges have moderate SNP-based heritability (mean ${h}_{SNP}^{2}=0.15$) and 54 genomic loci are significantly associated with variation of MIND similarity at one or more of the 276 constituent edges in each individual brain similarity network. On this basis, we addressed three linked questions concerning the genetic architecture of human cortical networks.

The long-standing dual origin theory of cortical evolution and development posits that mammalian cortex evolved along two genetically differentiated cytoarchitectonic trends of expansion and increasing lamination, originating from two specific loci of primitive cortical tissue^40–45^. If this theory is true, we reasoned, there should be evidence that genetic effects on structural similarity between cortical areas are systematically co-ordinated along two dimensions or gradients originating at or close to the theoretically predicted origins of cortical evolution: the piriform cortex (paleo-cortex) and the hippocampus (archi-cortex). We tested this hypothesis by analysing the MIND genetic correlation matrix, where each element represents the correlation between the GWAS statistics for each pair of 276 edges in the MIND network. We found that over 70% of the co-variation in the MIND genetic correlation matrix was reducible to two principal gradients, G1 and G2, which were tested for spatial alignment with the two theoretically predicted trends of paleo- and archi-cortical architectonic differentiation^41,42,44,45^. We found that the weighting of cortical areas on the first genetic gradient was uniquely highly correlated with geodesic distance from the paleo-cortical origin in piriform cortex; whereas cortical weighting on the second gradient was most correlated with distance from parahippocampal cortex, adjacent to the archi-cortical origin. Building on these results, we investigated two further key questions through the lens of these dual origin genetic gradients in human MIND networks.

We investigated the hypothesis that inter-areal metrics of structural similarity and functional connectivity (FC) should be genetically correlated. To that end, we estimated comparable GWAS statistics for single-subject fMRI connectivity networks measured in the same (discovery) cohort of UKB participants, and with identical cortical nodes and edges to the MIND networks. We found, as expected, that FC heritability was significantly less than MIND heritability, overall, but structural similarity across the paleocortical gradient was robustly genetically correlated with functional connectivity. Indeed, for the paleocortical trend specifically, Mendelian randomisation analysis indicated that genetically determined variation in structural similarity caused concordant changes in functional connectivity.

We next explored how the genetics of MIND were related to the genetics of clinical disorders or biomedical traits that putatively lie downstream of both structural similarity and functional connectivity phenotypes. Using global and local genetic correlation analysis, we identified multiple genomic loci of pleiotropic association with both dual origin trends of cortical similarity and trans-diagnostic clusters of more than one disorder or trait.

## Results and discussion

### Genome-wide associations with edges of inter-areal similarity

**Fig. 1:**
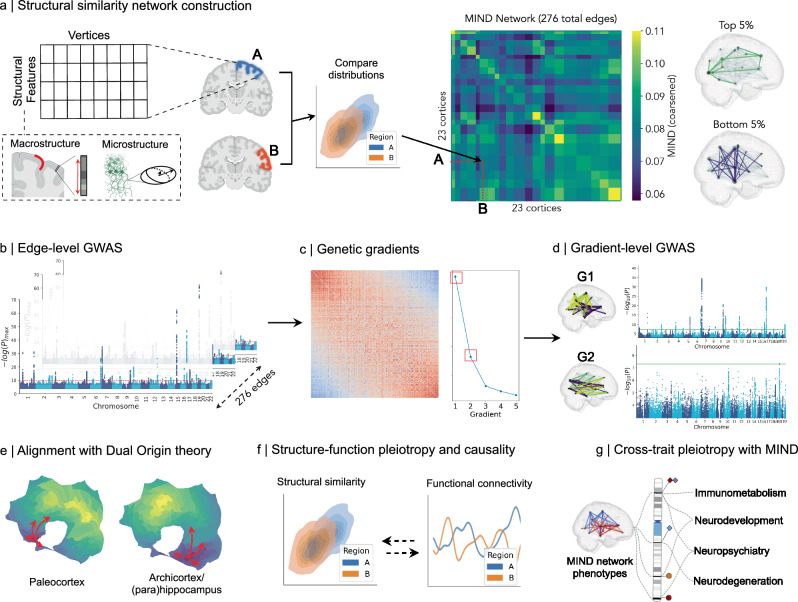
Overview of structural similarity network estimation by MIND and downstream analysis of the dual origins of MIND genetic gradients and their pleiotropic associations with functional and clinical phenotypes. **a** Schematic of the process for transforming an individual’s brain MRI scans into a MIND network comprising 276 edges between 27 cortical areas per hemisphere (see “Methods”). The group-averaged MIND network is shown as a heatmap (colour bar capped at 0.11 to aid visualisation). To the right of the heatmap, and sharing the same colorbar, the top and bottom 5% edges of the MIND network are shown on the brain. **b** We performed genome-wide association analyses for each of the 276 MIND network edges, and (**c**) identified latent genetic gradients underlying the edge-level signal. The Manhattan plots and gradient decompositions in (**b**, **c**) are illustrative. D) We performed two additional GWAS corresponding to MIND network connectivity, summarised along the axes defined by G1 and G2, the primary genetic gradients of MIND. **e** We analysed the spatial variation of the genetically-defined MIND gradients in the context of dual origin theory. **f** Using summary statistics from directly-comparable phenotypes from functional connectivity networks, we tested for both correlative and causal genetic overlap between structural similarity and functional connectivity. **g** Finally, we examined the global and local genetic overlaps between structural similarity and a variety of traits, including immunometabolic, neurodevelopmental, neuropsychiatric and neurodegenerative phenotypes. Created in BioRender. Sebenius, I. (2026) https://BioRender.com/fqdakhd.

MIND estimates similarity between cortical areas by comparing their vertex- or voxel-level distributions of one or more structural MRI features. Previous work has shown that a multivariate MIND network structure is not dominated by a single feature, and including a larger number of complementary features increases the alignment between structural similarity and biological benchmarks such as axonal connectivity and gene expression similarity^25^. On this basis, we aimed to use an integrative measure of structural similarity, representative of multiple, complementary aspects of cortical organisation. From the range of MRI features potentially available in the UKB MRI cohort, we therefore selected four MRI features that were interpretable, heritable, and complementary (see Methods for details): two macro-structural metrics - cortical thickness (CT) and mean curvature (MC); and two micro-structural metrics - mean diffusivity (MD) and neurite density index (NDI). For each participant, these four features were measured at every vertex within each of 360 predefined cortical regions (the “HCP" parcellation^46^). The similarity between each pair of HCP-defined regional multivariate distributions was estimated by the inverse of the Kullback-Leibler divergence between them^25^. The resulting {360 region  × 360 region} networks were coarse-grained by averaging inter-regional similarities for HCP-designated parcels assigned to the same one of 23 higher-order cortical areas. This led to a final set of subject-specific {23 area  × 23 area} structural similarity networks, each comprising 276 edges representing MIND similarity between each possible pair of 23 cortical areas (Fig. 1A).

**Fig. 2:**
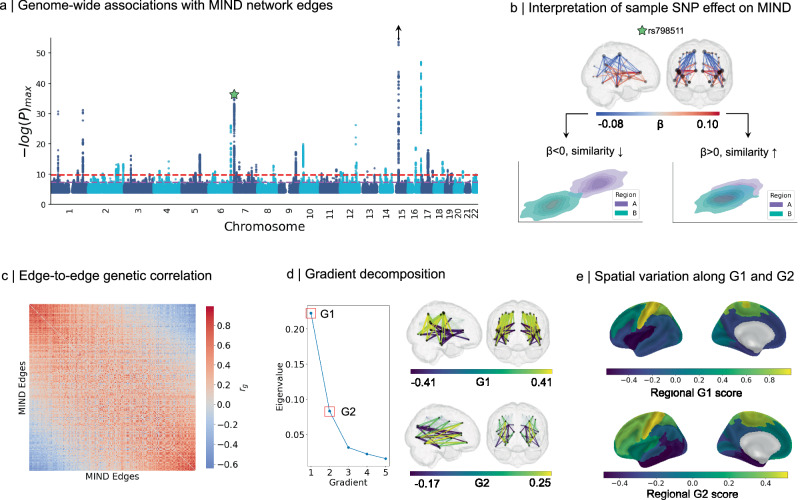
Edge-level genetic associations with structural similarity follow two underlying genetic gradients. **a** Manhattan plot summarising common variant associations (from GWAS regression) with MIND networks across all 276 studied network edges. After clumping, 54 approximately-independent genomic loci were identified at experiment-wide significance as being related to one or more MIND network edges (correcting for the number of variants and edges, *P* < 5*e* − 08/276 = 1.8*e* − 10). The *y*-axis indicates the most significant association of each variant across all edges. The purple line indicates genome-wide significance (*P* < 5*e* − 08), and the red line indicates experiment-wide significance (*P* < 1.8*e* − 10). A black arrow above a locus on chromosome 15 indicates that variants there reach higher significance than shown (up to  − *log*_10_(*P*) = 112).** b** An illustration of the distributed network effects of a leading MIND-associated variant (indicated by a green star in panel **a**) and their interpretation in terms of a graphical representation of similarity. In the brain network plot, only edges achieving genome-wide significance are shown, and the size of each node is proportional to the number of associated edges reaching genome-wide significance. Coronal views show each edge repeated twice - once per intrahemispheric segment of each of the 23 regions (which span cortical areas on both hemispheres). **c** Heatmap showing the genetic correlations between each MIND network edge, as measured by LD Score Regression (LDSC)^47^. The edges are ordered by the principal gradient of this edge-by-edge genetic similarity matrix (described in part D). **d** Eigenvalues of the first five gradients from decomposition of the edge-by-edge genetic correlation matrix in (**c**), with G1 and G2 highlighted. The top and bottom 5% of G1 and G2 loadings are shown on the brain, because decomposition was performed on an edge-by-edge matrix, each gradient is itself a network with one value per edge. **e** To interpret the spatial patterning of G1 and G2, edge-level gradient values were mapped to regional scores via gradient decomposition. Each regional score reflects how similarly a region varies along that gradient; for example, regions with similar G1 scores share similar edge-level G1 profiles. Regional scores are shown as cortical surface heatmaps.

Each of these edges of inter-areal similarity was treated as an independent phenotype in a set of 276 GWAS using a discovery sample of *N*_*max*_=30,524 individuals from the UKB (based on the same primary cohort as in ref. ^20^). MIND edges demonstrated moderate heritability (mean ${h}_{SNP}^{2}=0.15$; $0.06\le {h}_{SNP}^{2}\le 0.26$). At an experiment-wide threshold of *P* < 1.8 × 10^−10^ (i.e., *P* < 5 × 10^−08^/276), 54 independent genomic loci were associated with at least one of the 276 MIND network edges (Fig. 2A). We replicated these results by performing the same GWAS on an additional and independent validation cohort of *N*_*max *_= 18,769 UKB adult participants. To test generalisability of MIND genetic associations, we computed the genetic correlation using LD Score Regression (LDSC)^47^ between each edge in the discovery and validation cohorts (Supplementary Fig. 1). The mean genetic correlation was *r*_*g *_= 1.003 (0.57 ≤ *r*_*g*_≤ 1.8), with 274 of 276 edges (> 99%) significantly positively correlated (*P*_*FDR*_ < 0.05), indicating robust replicability of the GWAS statistics of MRI similarity network phenotypes.

MIND edge-associated genome loci exhibited both highly distributed effects on similarity across the network (e.g., *rs798511* shown in Fig. 2B) and highly specific effects on edges connected to a single cortical area (e.g., *rs492435*, shown in Supplementary Fig. 2A). However, on average, genetic variants that were more significantly associated with MIND at any edge were also associated with a higher number of edges (Supplementary Fig. 2B), suggesting a general tendency for distributed rather than localised effects of genetic variation on MIND network phenotypes.

### Gradients of genetically correlated inter-areal similarity

Given this evidence for distributed effects of localised genetic variation on similarity (or dis-similarity) of multiple pairs of cortical areas, we estimated the genetic correlations between all possible pairs of MIND edges, compiled as a {276 edge  × 276 edge} genetic correlation matrix, *G* (Fig 2C). There were evidently many strong genetic correlations between edges in the network, and we used gradient decomposition^48^ to summarise genetic effects on MIND networks in terms of the first two gradients, G1 and G2 (Fig 2D). Together, they accounted for over 70% of the (co)variance in the inter-areal genetic correlation matrix, *G* (see “Methods” for details), suggesting that they represent major organisational principles rather than oversimplified representations of the genetic architecture of the human cortex. As the gradient decomposition was performed on an edge-by-edge matrix, each of the resulting gradients can also be considered as a network, with each edge weighted by its coefficients on G1 or G2.

To further investigate the genetic architecture of these two principal gradients, we first estimated gradient-level phenotypes by the sum of MIND edges in each individual network weighted by their coefficients on each of G1 and G2, using methods previously described for analysis of whole brain gradients of gene expression^49^. Then we performed two additional GWAS of the gradient-level phenotypes. We found that G1 and G2 were genetically independent of each other, evidenced by a non-significant genetic correlation between them (LDSC; *r*_*g*_ = 0.08, *P* = 0.18). Both MIND gradients were more heritable than MIND edges (on average): ${h}_{G1}^{2}=0.281\pm 0.035$; ${h}_{G2}^{2}=0.192\pm 0.022$ (Supplementary Figs. 3C and 4A). G1 was significantly associated with 31 loci at the genome-wide threshold of *P* < 5 × 10^−8^; G2 was associated with a single locus at this threshold (see Supplementary Fig. 3A). After additionally correcting for two gradient-level tests, G1 and G2 were significantly associated with 26 and 0 loci, respectively. This discrepancy between the number of associated loci for G1 and G2 was consistent with our finding that G1 was roughly ten times *less* polygenic than G2 (*π*_*G*1_ = 0.33%, *π*_*G*2_ = 3.7% using SBayesS; Supplementary Figs. 3C)^50,51^. This estimate of polygenicity for G1 was also substantially lower than the values reported for any of 206 measures of DTI-derived white-matter connectivity (0.8% ≤ *π*_*DTI *_≤ 9.6%, median *π* = 7.5%) from a recent GWAS^7^, suggesting that it is influenced by an unusually concentrated set of genes compared to both G2 and other structural brain connectivity phenotypes. In addition, we observed that G1 was under significantly stronger negative selection (*S*) compared to G2 (*S*_*G*1_ = − 0.58, *S*_*G*2_ = − 0.08 using SBayesS; Supplementary Fig. 3C). The combination of higher heritability (*h*^2^), lower polygenicity (*π*), and more negative *S* suggested that genetic influences on G1 were stronger, more genomically localised and evolutionarily conserved than genetic effects on G2.

We next studied the extent to which MIND gradients G1 and G2 were genetically related to other commonly-studied measures of whole-cortex structure. As shown in Supplementary Fig. 4B, genetic correlations between G1/G2 and whole-brain CT, MD, ICVF, MC, and surface area (SA)^20^ were generally weak-moderate (0.04 < ∣*r*_*g*_∣ < 0.36 for all comparisons), although four of these comparisons were statistically significant (G1-SA *r*_*g*_ = − 0.12; ICVF-G1 *r*_*g*_ = − 0.36; G2-SA *r*_*g*_ = − 0.19; G2-MD *r*_*g*_ = 0.19, all *P*_*FDR*_ < 0.05). Of note, the weak genetic correlations between global surface area and both G1 and G2 indicated that MIND gradients were not simply attributable to genetically determined allometric scaling of cortex. Some degree of overlap with prior structural imaging GWAS is nonetheless expected, and is reflected both in these global correlations and at the level of individual loci — for example, *rs798511* (Fig. 2B) has previously been linked to ventricular size^52^. However, the generally low magnitude of these genetic correlations was not sufficient to explain the genetic architecture of multivariate MIND gradients in terms of the genetics of univariate MRI phenotypes.

### Gradients of genetically correlated cortical similarity are aligned with dual origins

The dual origin theory of cortex emerged from histological studies of phylogenetically primitive mammals and reptiles in the early 20th century^40–43^ which suggested that the complex 6-layer lamination of isocortex (a.k.a. neocortex) evolved as a function of distance from two conserved allocortical areas or primordial anlagen, the paleocortex and the archicortex, which have simpler 3-layer lamination. This theory has been revitalised to explain not only evolutionary trends in cytoarchitectonic differentiation but also the ontogeny of brain structure^43,53^, brain network architecture^54^, and the functionally specialised organisation of dorsal and ventral sensory processing streams in the adult human cortex^55,56^. Dual origin theory is often assumed to have a neurogenetic basis, with recent research suggesting that spatial patterns of genetically-determined variation in human cortical structure are anatomically consistent with the cytoarchitectonic trends predicted from the pre-human evolution of cortex^44,45^. However, the evidence is currently somewhat inconsistent and incomplete, and the genetic mechanisms underlying these expected trends have not been clearly articulated. For instance, the principal genetic gradient of regional cortical thickness did not align, as theoretically predicted, with maps of geodesic distance from either paleocortical or archicortical origins^44^.

**Fig. 3:**
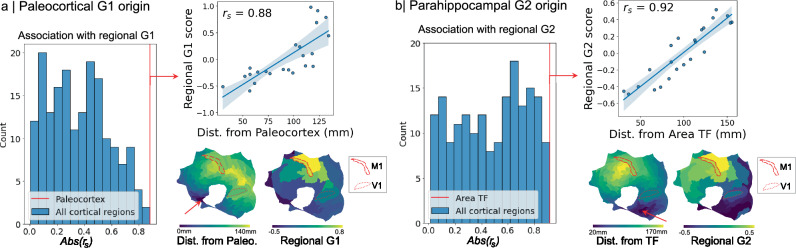
MIND genetics vary along the predicted trends of dual origin theory. **a** The histogram indicates the alignment between the regional G1 scores and cortical maps of geodesic distance from each of 180 cortical regions from the HCP parcellation, plus separately-constructed maps of geodesic distance from the paleocortex and archicortex (the latter of which comprises multiple cortical regions) (*Abs*(*r*_*s*_) = Absolute Spearman’s correlation). The red line indicates the maximum association between regional G1 scores and region-specific distance, which corresponded to the paleocortical distance map. This association between regional G1 and paleocortical distance is shown to the right as a scatterplot (shading indicates 95% CI of the regression estimate). Beneath, the two cortical flatmaps show (left) the distance map from the paleocortex to each of 180 regions (bilaterally-averaged), with the paleocortex indicated by a red arrow, and (right) the regional G1 scores. **b** The histogram is comparable to the one shown in part A, but for regional G2 scores. Distance from the parahippocampal area TF most closely aligned with regional G2 scores; this association between area TF and regional G2 is shown to the right as a scatterplot. The two cortical flatmaps show (left) the distance map from area TF to each of 180 regions (bilaterally-averaged), with area TF indicated by a red arrow.

We tested the hypothesis that the two principal gradients of the MIND genetic correlation matrix, G1 and G2, were anatomically aligned with the two trends of cortical differentiation predicted by dual origin theory. To do this, we mapped the edge-level weights on each gradient to an areal patterning via a second round of gradient decomposition that mapped the two {23 area  × 23 area} matrices of weights of each edge on G1 and G2 to two vectors representing the weight of each cortical area on each of the genetic gradients (Figs. 2E, 3). If dual origin theory is correct, we reasoned, areal weights on the two genetic gradients should be closely linked to geodesic distance from one of the two cortical origins, with more distant regions having more divergent G1 or G2 scores.

The origin of the paleocortical trend is clearly identified as the piriform cortex, a small, discrete region adjacent to the anterior insula. As theoretically predicted, there was a strong correlation (Spearman’s *r*_*s *_= 0.88) between a cortical map of G1 weights and a prior map of geodesic distance from the piriform cortex (Fig. 3A). To test the robustness and specificity of this association, we also calculated the correlations between areal G1 weights and maps of geodesic distance from each of the 180 (bilaterally-averaged) cortical areas designated by the HCP parcellation, which were coarse-grained to the 23 higher order areas for comparison. We found the cortical map of G1 scores was more strongly correlated with distance from the piriform cortex than with distance from any other cortical area (Fig. 3A), confirming that there was a uniquely strong alignment between the principal gradient of genetically determined (dis-)similarity between cortical areas and their distance from the paleocortical origin.

The origin of the archicortical trend in humans is more ambiguously defined, reflecting the more extensive, quasi-circular or limbic swathe of allocortex from the ventral hippocampus through parahippocampal and retrosplenial cortex to the dorsal remnants of hippocampus (indusium griseum and longitudinal striae)^57^. Therefore, to test the hypothesis that G2 was aligned with distance from the archi-cortical origin, we did not specify the origin a priori but calculated the correlations between areal G2 scores and geodesic distance from all 180 cortical regions in the HCP parcellation. We found that G2 scores were most closely aligned with distance from parahippocampal area TF (*r*_*s *_= 0.92; Fig. 3B), an isocortical area adjacent to allocortical areas of the ventral hippocampus, which forms part of the evolutionary origin of the archi-cortical trend. However, areal G2 scores were not correlated with a prior map^56^ of the archicortical trend in adult humans, which indexes distance from the entire limbic swathe of the archicortex (*r*_*s*_ = − 0.03). In short, the second principal gradient of genetically coordinated patterning of cortical similarity, G2, was aligned with the archicortical trend (in contrast to the alignment of G1 with the paleocortical trend), broadly as predicted by dual origin theory. However, some ambiguities remain to be resolved, like the theoretically optimal origin for archicortical trend mapping in human MRI, before the putative identification of G2 with an archicortical trend of MIND similarity is as robust and specific as the identification of G1 MIND with the paleocortical trend of mammalian cortical evolution and expansion.

The dual origin theory was not discovered genetically, but by observing patterns of phenotypic variation of the cortex across species. We therefore reasoned that a similar alignment to dual origin trends would be observed if MIND gradients were defined phenotypically, instead of genetically. To test this, we calculated the phenotypic covariance matrix *P*, representing the between-subject co-variation in MIND similarity for each possible pair of cortical areas, and found that this was strongly coupled with the genetic correlation matrix *G* (*r*_*s*_ = 0.85), as predicted by Cheverud’s conjecture^58^ and previous work on structural MRI genetics^59^ (Supplementary Fig. 5). The first two principal gradients derived from the phenotypic correlation matrix *P*, P1 and P2, were highly similar to G1 (*r*_*s*_ = 0.97) and G2 (*r*_*s*_ = 0.95), and their areal patterning was also correlated with areal G1 scores (*r*_*s*_ = 0.99) and G2 scores (*r*_*s*_ = 0.89), respectively.

As previously noted, G1 and G2 were genetically independent of each other (LDSC *r*_*g *_= 0.08, *P* = 0.18). Spatially, however, the cortical patterning of areal weights on G1 and G2, i.e., the parcellated cortical maps of paleo-cortical and archi-cortical trends, were not fully orthogonal (*r*_*s *_= 0.42, *P* = 0.05). Spatial co-location or convergence of these genetically independent cortical trends was more obvious for isocortical or koniocortical areas of higher laminar complexity at greater geodesic distance from the allocortical origins. For example, G1 and G2 originated from distinct allocortical locations (in piriform and parahippocampal cortex, respectively) but terminated in the same area of koniocortex (somatosensory cortex). Neither gradient was colocated with cortical maps of evolutionary expansion or areal allometric scaling, suggesting that the observed dual origin trends did not simply reflect changes in cortical size across species or individuals (Supplementary Fig. 4D).

Finally, we compared the spatial patterns of MIND G1 and G2 with the genetic principal components of each of several structural MRI features^20^. Areal G1 and G2 scores showed varying degrees of spatial alignment with the genetic PCs of individual MRI features (0.06 < ∣*r*_*s*_∣ < 0.77; Supplementary Fig. 4C). Interestingly, strong spatial alignments (e.g., areal G1-CT PC1, ∣*r*_*s*_∣ = 0.77) did not reflect strong underlying genetic correlations (G1-global CT, *r*_*g*_ = − 0.04; Supplementary Fig. 4B), suggesting that similar spatial patterning did not necessarily imply genetic concordance. Paleocortical distance was more strongly associated with areal G1 than with the first genetic principal component of any individual structural feature (Supplementary Fig. 4D). Similarly, distance from parahippocampal area TF was more strongly correlated with areal G2 than with any of the univariate genetic principal components. In addition, areal G2 showed stronger correlations with brain maps indexing laminar complexity of cortical myeloarchitecture (i.e., the first microstructural profile covariance gradient; ∣*r*_*s*_∣ = 0.89) and cytoarchitecture (i.e., externopyramidization; ∣*r*_*s*_∣ = 0.72) than did individual feature PCs (Supplementary Fig. 4D). Overall, these results indicated that the integrative, multivariate MIND gradients G1 and G2 were more precisely aligned with the anatomical predictions of dual origin theory than the genetic principal components of any of the individual, univariate structural MRI features that were used to estimate MIND.

Overall, these results support our prediction that the two principal gradients of genetically coordinated effects on MIND similarity networks should be anatomically aligned with the paleocortical (especially) and archicortical trends of dual origin theory^44,45,54^. As such, they show how dual origin theory can provide a framework for describing and genetically explaining two major trends of cortical differentiation in the human brain, in addition to its original focus on the evolutionary progression of cortical structure across species. More broadly, these results suggest that the dual origin framework may serve as a unifying interpretation for other cortical trends identified in previous neuroimaging and genetics studies^14,20^ — a possibility that warrants systematic investigation in future work.

### Genetic relationships between structural similarity and functional connectivity

These gradients of genetically coordinated structural similarity, as evident in human MRI data and predicted by dual origin theory, might be expected to have some consequences for the functional organisation of brain networks. MIND similarity can be compared directly to functional connectivity (FC) metrics, such as the correlation between resting-state fMRI time series, since both MIND and FC are relational measures that can be quantified between the same set of cortical areas. There is a large but inconclusive literature on the phenomenon of structure-function coupling of connectivity, typically estimated by the correlation of FC and structural connectivity metrics derived from axonal tractography of diffusion (d)MRI data, which is characterised by low subject-specificity and poor reliability across studies or processing measures^60,61^. Here, we tested the hypothesis that structural similarity (MIND) and functional connectivity (FC) metrics are robustly genetically coupled, and explored the extent to which structure-function coupling is differentially linked to the twin gradients of cortical genetic architecture.

**Fig. 4:**
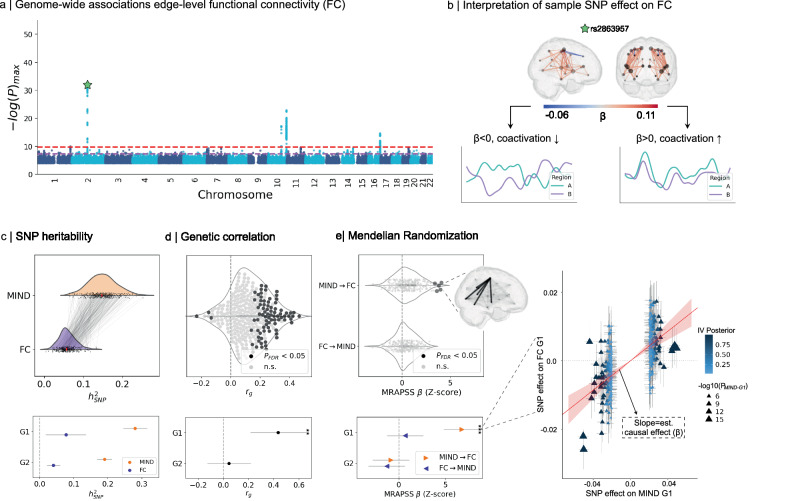
Genetic architecture of functional connectivity and its correlational and causal coupling with MIND similarity in paleocortical and archicortical trends. **a** Manhattan plot summarising common variant associations (from GWAS regression) with functional connectivity (FC) network edges. After clumping, 4 approximately-independent genomic loci were associated at *P* < 1.8e-10 with one or more FC network edges. As in Fig. 2, the *y*-axis indicates the most significant association of each variant across all edges. The purple line indicates genome-wide significance (*P* < 5*e* – 08), and the red line indicates experiment-wide significance (*P* < 1.8*e* – 10). **b** Illustration of the meaning of positive and negative GWAS *β* coefficients for a sample SNP in terms of functional connectivity. The brain network shows all edges positively or negatively associated to the SNP indicated by a green star in panel (**a**); positive weights indicate greater positive FC (co-activation), and negative weights indicate reduced FC and co-activation, as shown schematically. **c** SNP-based heritability estimates for MIND and FC. The top plot shows a histogram of edge-level heritability estimates (HDL)^91^. The bottom plot shows mean SNP-based heritability (95% CI) for MIND, and FC projected into the lower-dimensional space of the MIND-derived G1 and G2 gradients. **d** Genetic correlations between MIND and FC. The top plot shows the distribution of edge-level genetic correlations across all 267 cortical edges; solid points indicate 81 significant correlations after FDR correction (*α* = 0.05). The bottom plot shows gradient-level genetic correlations (95% CIs) between MIND and FC for G1 and G2. **e** Causal relationships between MIND and FC. The top plot shows *Z*-scored causal effects in both directions (MIND  → FC and FC  → MIND) across all 276 edges; solid points are significant after FDR correction (*α* < 0.05). The inset brain network shows edges where MIND had a significant causal effect on FC. The bottom plot shows gradient-level forward and reverse causal estimates (95% CI) for G1 and G2. The right plot illustrates the significant causal effect of MIND G1 on FC1 along the paleocortical trend^62^, shown as a regression with positive slope (shading: 95% CI). In (**c**–**e**), boxplots indicate mean values, quartiles (box bounds), and 1.5  × interquartile range (whiskers).

To do this, we conducted an additional set of 276 GWAS on the same set of 276 edges as defined for the MIND networks, and in a subsample (*N*_*max *_= 30,376) of the same UK Biobank participants; but now with each edge representing the correlation between regional mean fMRI time series recorded with the participant in a resting state (Fig. 4A). These edge-level GWAS for the relational measure of functional connectivity were thus estimated in the same individuals, using an identical cortical parcellation, as previously used for edge-level GWAS of MIND, a relational measure of cortical similarity. This methodological alignment between MIND and FC allowed us to directly compare the genetics of cortical similarity and functional connectivity on a like-for-like, unbiased basis. We also investigated the genetics of inter-individual variation in the aggregate strength of FC between areas weighted by the two MIND gradients, G1 and G2. For each individual participant, we weighted and summed their FC matrix by the MIND-derived gradients G1 and G2, thereby projecting each FC network onto two gradients, FC1 and FC2, in the (directly-comparable) space of the paleo-cortical (G1) and archi-cortical (G2) trends in MIND similarity networks.

At the phenotypic level of edges, MIND was more heritable than FC for 97.8% of edges, with an average SNP-based heritability nearly three times greater than that of FC (${h}_{SNP}^{2}=0.148$ for MIND versus 0.058 for FC; see Fig. 4C). At the phenotypic level of gradients, FC1 was marginally (though not significantly) more heritable than FC2, (Fig. 4C; ${h}_{SNP}^{2}$ = 0.077 ± 0.058 for G1, ${h}_{SNP}^{2}$ = 0.045 ± 0.019 for G2); although both FC1 and FC2 had much lower heritabilities than G1 and G2, respectively (Fig. 4C). The relatively low heritability of functional connectivity phenotypes is in line with prior results^9^ and demonstrates that FC is less strongly influenced by common genetic variation than MIND. In comparison to MIND, which showed substantial differences in both polygenicity (*π*) and negative selection (*S*) between G1 and G2, there were no significant differences between FC1 and FC2 on these parameters (*π*_*FC*1_ = 0.007 [0.004, 0.011], *π*_*FC*2_ = 0.01 [0.005, 0.017]; *S*_*FC*1_ = − 0.52 [−0.96,  −0.8], *S*_*FC*2_ = −1.09 [−1.30,  −0.86]; Supplementary Fig. 6A).

Despite the relatively low heritability of functional connectivity phenotypes, we found solid evidence that they were genetically correlated with structural similarity phenotypes. Specifically, genetic correlations between MIND and FC edges were positive for 86% of network edges, with mean *r*_*g *_= 0.12 (−0.23 ≤ *r*_*g *_≤ 0.46). There were significant positive genetic correlations at 28% of edges (*P*_*FDR*_ < 0.05), in contrast to significant negative correlations at about 1% of edges. Phenotypic correlations across individuals between MIND and FC edges were also generally positive (*r*_*s*_ > 0 for 83% of edges;  −0.06 ≤ *r*_*s *_≤ 0.13) and were moderately associated with the corresponding genetic correlations (*r*_*s*_ = 0.36, *P* < 0.001).

The genetic relationship between MIND and FC was clarified and strengthened through gradient-level comparison. On the paleocortical trend represented by G1, MIND G1 scores and FC1 scores were significantly genetically correlated at approximately the same level as the most strongly genetically correlated edges (HDL *r*_*g*_ = 0.44, *P* = 1.2*e* − 04; LDSC *r*_*g*_ = 0.46, *P* = 6.5*e* − 08). FC1 was not significantly genetically correlated with global measures of brain structure (CT, MC, ICVF, MD, or SA; Supplementary Fig. 6B), suggesting that the genetic overlap between MIND G1 and FC1 reflected a shared network organisation rather than associations with simpler univariate structural metrics. However, on the archicortical trend represented by G2, there was no significant genetic correlation between MIND G2 and FC2 scores (HDL *r*_*g*_ = 0.04, *P* = 0.61; LDSC *r*_*g *_= 0.05, *P* = 0.76; Fig. 4D). This result suggests that the two genetically independent trends of cortical architecture, defined by structural MRI network analysis and aligned with dual origin theory based on cortical histology, have quite distinct relationships to brain functional activity.

We further investigated the potentially causal relationships between structural similarity and functional connectivity using Mendelian randomisation (MR). We fit MR models for each MIND and FC network edge, as well as for the paired gradients G1/FC1 and G2/FC2, in both the forward (MIND  → FC) and reverse (FC  → MIND) directions using MR-APSS, a robust method that corrects for horizontal pleiotropy as well as the presence of overlapping samples^62,63^.

At the level of edges, we found evidence for a significant forward causal relationship (MIND  → FC) at multiple edges (*P*_*FDR*_ = 0.05 across 276 tests; Fig. 4E), but no evidence for significant reverse causal relationships. At the level of gradients, these findings came into sharper focus, showing a strong and specific forward causal relationship between MIND G1 and FC1 (Fig. 4E; *Z*-scored *β*=6.8, *P* = 1.4*e* − 11); but no significant forward causal relationship between MIND G2 and FC2, and no significant reverse causal relationships between FC1 and G1 or FC2 and G2. The causal relationship of MIND G1 on FC1 was significantly replicated when using MIND summary statistics from the (non-overlapping) replication cohort in a strict two-sample setting (IVW *β* = 0.19, *P* = 1.3*e* − 05; Weighted Median *β* = 0.14, *P* = 1.7*e* − 03; Supplementary Fig. 7B), and was robust across a range of commonly-used two-sample MR methods and sensitivity analyses (Supplementary Figs. 6C–D, 7).

Taken together, these results position MIND as a useful bridge between genetics, univariate brain structure, and functional connectivity: MIND being a structurally-derived relational measure that is substantially more heritable than the functionally-derived relational measure of FC, while also being robustly and concordantly correlated with FC genetically. Given the relatively low heritability of the FC phenotypes studied, and the short duration and/or low signal-to-noise of fMRI time series data available at sufficient scale to support GWAS, the observed genetic overlaps between MIND and FC likely represent the lower bound of their true genetic relationship.

More specifically, these results suggest that the strong genetic correlation between functional connectivity and structural similarity in the paleocortical trend represented by G1 is the outcome of genetically driven effects on structural similarity causally constraining functional connectivity. This evidence aligns with prior work suggesting that brain morphology constrains brain function, but not vice versa^45,64^. More generally, the concordant correlational and causal relationships between MIND and FC, at least in the paleo-cortical gradient, are consistent with a *homophilic* interpretation, whereby genetically-coordinated structural similarity is associated with (and/or causes) functional connectivity and is predictive of anatomical connectivity^26^. Brain networks have previously been shown to demonstrate homophilic properties across diverse experimental scales and modalities (from neuronal cultures to human MRI scans) and across a wide range of similarity metrics (including topological assortativity, genomic co-expression, and cytoarchitectonic profile covariation)^65–68^. Here, the alignment between MIND to FC provides genetic and causal support for these diverse observations and simulations of homophily as an important organising principle of brain networks.

### Pleiotropic associations between dual origin gradients and biomedical traits

Structural and functional brain network organisation is the outcome of neurodevelopmental processes and can be atypical in neurodevelopmental or neurodegenerative brain disorders^1,69,70^. Recent GWAS results have provided some evidence for genetic correlations between brain network phenotypes and neuropsychiatric disorders, as well as pleiotropic associations between structural MRI phenotypes and a wide range of other biomedical traits^17,59,71–74^. As such, we expected to observe pleiotropic associations between MIND gradients G1 and G2 (and their related functional connectivity gradients, FC1 and FC2) and a range of biomedical traits. In light of prior work theorising that the paleocortical and archicortical trends of dual origin theory are differentially implicated in the pathogenesis of brain disorders^75^, we anticipated that G1 and G2 might be genetically linked to distinct clinical diagnostic categories or health-related traits. We therefore estimated the genetic correlations between the dual origin gradients of structural similarity and functional connectivity and 6 neuropsychiatric disorders and 3 biomedical traits.

The paleocortical trend of structural similarity represented by G1 was globally genetically correlated with schizophrenia (SCZ; *r*_*g*_ = 0.08, *P* = 0.03 corrected for two gradient-level tests). Follow up analysis provided tentative evidence for a forward (but not reverse) causal effect of G1 on schizophrenia (G1 → SCZ, MR-APSS *β *= 0.037, *P* = 0.04 uncorrected; SCZ → G1, MR-APSS *β *= − 0.004, *P* = 0.90 uncorrected; Supplementary Table 1), consistent with the possibility that paleocortical architecture may contribute to schizophrenia risk. The archicortical trend represented by G2 was globally genetically correlated with multiple disorders or traits, including SCZ (*r*_*g*_ = − 0.09, *P* = 0.02), major depressive disorder (MDD; *r*_*g*_ = − 0.15, *P* = 6.3*e* − 05), attention deficit hyperactivity disorder (ADHD; *r*_*g*_ = − 0.11, *P* = 0.02), C-reactive protein concentration in serum (CRP; *r*_*g*_ = − 0.11, *P* = 0.004), and body mass index (BMI; *r*_*g*_ = − 0.13, *P* = 1*e* − 04). Subsequent MR analysis demonstrated no forward causal effect of G2 on any disorders or traits (Supplementary Table 1). However, there was strong evidence for a reverse causal effect of BMI on G2 (MR-APSS *β *= − 0.15, *P* = 8.0*e* − 04 uncorrected) and suggestive evidence for a reverse causal effect of CRP on G2 (MR-APSS *β *= − 0.07, *P* = 0.045 uncorrected), indicating that immuno-metabolic health may have downstream effects on archicortically aligned trends of cortical architecture. These results provide preliminary support for the for the prior prediction arising from dual origin theory^43^ that the two trends of cortical architectonic differentiation may be differentially implicated in the pathogenesis of neuropsychiatric disorders, though replication of the putative causal links between them represents an important direction for future work.

**Fig. 5:**
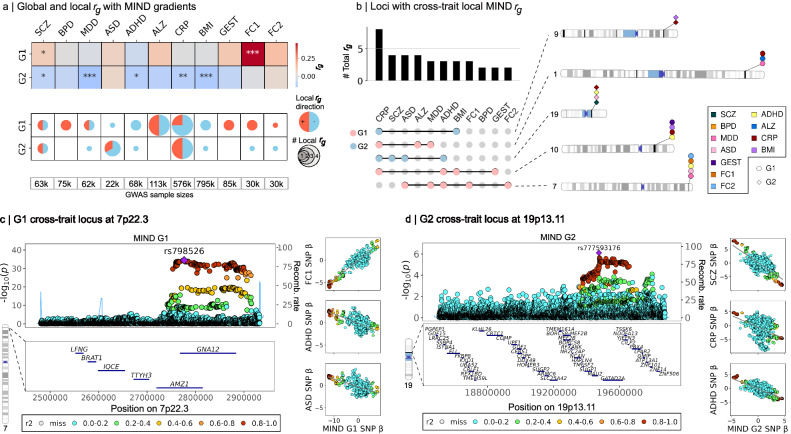
Pleiotropic associations between MIND genetic gradients, neuropsychiatric disorders and biomedical traits. **a** Magnitude (and significance) of global genetic correlations (top row) and number and sign of significant local genetic correlations (middle row) between MIND genetic gradients, G1 and G2, and each of 6 neuropsychiatric disorders (SCZ, schizophrenia; BPD; bipolar disorder; MDD, major depressive disorder; ASD, autism spectrum disorder; ADHD, attention-deficit hyperactivity disorder; or ALZ, Alzheimer’s disease), each of 3 biomedical traits (CRP, C-reactive protein; BMI, body mass index; GEST, gestational age at birth), and both functional connectivity gradients, FC1 and FC2 (11 columns in total). The bottom row reports the size of all comparison GWAS, with estimated effective sample size reported for case-control traits and total sample size for quantitative traits (see Supplementary Table 2). Global genetic correlations were corrected for the number of gradients (2 tests), and local genetic correlations were corrected for both the number of independent genomic regions and gradients (2, 262 × 2 = 4, 524 tests). ***P < 0.001, **0.01 < *P* < 0.001, *0.01 < *P* < 0.05. **b** Visualisations of the five cross-trait loci where ≥ 2 outcomes were significantly locally genetically associated with MIND G1 or G2. The barplot indicates the total number of significant local *r*_*g*_s associated with each phenotype and either G1 or G2, of which the five cross-trait loci are highlighted below and in the chromosome maps to the right. **c** Visualisation of the shared local genetic effects between three comparison phenotypes (FC along G1, ADHD, and ASD) sharing a significant genetic correlation with MIND G1 at the locus spanning 2.5–2.9 mb on chromosome 7p22.3. The locus plot shows the genes within the region and the variant-level association with MIND G1 (the plotted log(P) values were uncorrected from the original GWAS regression). The stacked scatter plots to the right illustrate the local genetic correlation by showing the (*Z*-scored) regression *β* values of the same regional variants for MIND and each of FC1, ADHD, and ASD. **d** The same plots as in part (**c**), but for MIND G2 at a locus within 19p13.11 where it is locally genetically correlated with SCZ, CRP levels, ADHD, and (not shown) ASD.

It has been shown that global genetic correlations only incompletely capture the shared genetic effects on brain structure and risk of psychiatric disorders^74^, due to the admixture of local correlations in discordant directions (both negative and positive), or noise introduced by large and unrelated portions of the genome. We therefore estimated a comprehensive set of local genetic correlations between dual origin gradients, clinical disorders, and biomedical traits (Fig. 5A–D)^76^. We observed a higher number of significant local genetic correlations as compared to global genetic correlations (86% of MIND-trait pairs had ≥ 1 significant local *r*_*g*_ versus 32% with significant global *r*_*g*_), with many local correlations in discordant directions (Fig. 5A). Although MIND gradient G1 had fewer significant global genetic correlations with diagnostic and biomedical traits than G2, it had more significant local genetic correlations (indeed, MIND G1 was significantly locally correlated with every studied phenotype). For example, by global genetic correlation analysis, CRP, ADHD, and MDD were only significantly linked to G2, not G1; but all these traits had more significant local genetic correlations with G1 than with G2. Local genetic correlations specifically linked G1 (and not G2) to Alzheimer’s disease (four loci), bipolar disorder (three loci), and gestational age at birth (three loci) - associations that were not apparent at the global level (Fig. 5A).

Of the 24 genomic loci locally correlated with one or both of the dual origin gradients and one or more clinical diagnoses or biomedical traits, five loci were associated trans-diagnostically with multiple neuropsychiatric disorders (Fig. 5B). At one of these loci (within 7p22.3, shown in Fig. 5C), G1 was not only associated with multiple disorders (MDD, ADHD, and ASD) but was also associated strongly and positively with functional connectivity (FC1 local *r*_*g*_ ~ 1, *P* < 1.0*e* – 10; FC2 local *r*_*g*_ = 0.82, *P* = 3.2*e* − 08). Colocalisation analysis^77^ between G1 and FC1 suggested a high probability (0.928) of a shared causal variant, with the 95% credible set containing multiple eQTLs of *GNA12* and *AMZ1* such as *rs798526*, the SNP most strongly associated with G1, as highlighted in the gradient-level Manhattan plot in Supplementary Fig. 3A. Visual inspection showed that significant variants at this locus cleanly straddled the length of *GNA12* (Fig 5C). *GNA12*, encoding GCPR *α*-subunit 12, has been causally linked to several potentially relevant neurobiological processes, including transmembrane signalling and regulating neuronal migration in the developing mouse cortex^78^. Building on these findings, we suggest that this locus represents a specific and plausible set of genes pleiotropically associated with paleocortical trends in structural similarity (G1) and directly-comparable functional connectivity, as well as trans-diagnostic risk of several neuropsychiatric disorders.

Multiple pleiotropic associations were also observed for G2. For example, as shown in Fig. 5D, G2 was locally correlated with both a systemic inflammatory marker (CRP) and three neuropsychiatric disorders (ASD, ADHD, and SCZ) at one locus on 19p13.11, including *GATAD2*, which is a well-established schizophrenia risk gene linked to neurodevelopmental abnormalities^79,80^. This locus represents a putatively distinct, neuroinflammatory mechanism for pleiotropic association of archi-cortical structural similarity and trans-diagnostic risk of neurodevelopmental disorders.

### Conclusion

Here, we investigated the genetic architecture of human cortical networks derived from structural MRI measures of the similarity between cortical areas. We concentrated our analysis of these data on predictions or questions arising from the dual origin theory of cortex, which originated in histological studies of non-human species published about 100 years ago, but which had not yet been widely translated to (ante-mortem) genetic studies of human cortical structure, function, and disorder.

We found compelling evidence that the paleocortical trend predicted by dual origin theory conforms to the first principal axis of genetically coordinated variation in the human cortical architectome, gradient G1. There was also strong evidence that the second principal axis, gradient G2, was aligned with the theoretically predicted archi-cortical trend. These results suggest that the twin cortical trends of dual origin theory, originally defined to describe phylogenetic variation of the cortex across mammalian species, can also provide a scaffolding for analysis of genetically-mediated variation in structural MRI similarity networks measured within and between individual human brains. This bridge between dual origin theory and human network neuroscience has allowed us to test some of the broader predictions of dual origin theory for human brain function and health. We have shown that these two genetically independent but anatomically converging gradients of cortical similarity are distinctly related to the genetic architecture of brain functional networks and pleiotropically associated with trans-diagnostic clusters of neuropsychiatric disorders and/or biomedical traits. These results generate many further questions about the detailed mechanisms of human cortical evolution and development of dual origin trends of inter-areal similarity, and their potential implications for a more neurogenetically grounded analysis of brain functional networks and pathogenic pathways to neuropsychiatric disorders. The GWAS summary statistics of MIND similarity network phenotypes (edges and gradients G1 and G2) and fMRI functional connectivity (edges and gradients FC1 and FC2) are made openly accessible to the research community to support further investigations into the origins and trends of the human cortical architectome.

## Methods

### Dataset

From the roughly 500,000 participants in the UK Biobank, our primary analysis was conducted using data from a total of 40,715 participants with magnetic resonance imaging (MRI) data available in 2023, of which 34,988 had the full set of MRI sequences and sufficient scan quality necessary to construct MIND networks. This study used the UKB genetic data that was previously quality-controlled and imputed before access as described by ref. ^81^, and used largely the same set of participants and genetic inclusion criteria as described in ref. ^20^. Specifically, participants were included based on the following genetic criteria: (1) self-identification as ethnically white, (2) within  ± 5 standard deviations of the mean along the first two genetic principal components, (3) matched genetic and reported sex, and (4) no excessive heterozygosity as reported by UKB. We included all SNPs with minor allele frequency greater than 0.1%, leading to the analysis of a maximum of 13,587,992 SNPs and 31,544 participants passing genetic inclusion criteria for the primary GWAS.

In 2024, the requisite MRI data for MIND network construction became available for an additional 21,447 UKB participants. This independent cohort formed our replication GWAS dataset. We applied the same genetic inclusion criteria to this validation cohort as for the original discovery cohort, leading to a maximum of 18,769 participants available for the replication GWAS.

### Magnetic resonance imaging (MRI) data

We used T1-weighted, diffusion-weighted, and resting-state functional MRI scans in the GWASs of UKB data. We additionally accessed T2-FLAIR images if available to facilitate surface reconstruction. The full imaging protocol can be found at: https://www.fmrib.ox.ac.uk/ukbiobank/protocol/V4_23092014.pdfand https://biobank.ctsu.ox.ac.uk/crystal/crystal/docs/brain_mri.pdf. In brief, the acquisition parameters for each type of data were the following, as described in refs. ^20,82,83^:

- **T1-weighted structural imaging (T1-w)**: 1.0 mm isotropic resolution, TR = 2000 ms, TE = 2.01 ms, TI = 880 ms, flip angle = 8 degrees.
- **T2-weighted fluid-attenuated inversion recovery (T2-FLAIR)**: 1.0  × 1.0  × 1.1 mm resolution, TE = 395.0 ms, TR = 5000 ms, TI = 1800 ms.
- **Diffusion-weighted imaging (DWI)**: 2.0 mm isotropic resolution, MB = 3, R = 1, TE = 92 ms, TR = 3600 ms, PF = 6/8, fat saturation, *b* = 0 s/mm^2^ (5x + 3x phase-encoding reversed), *b* = 1000 s/mm^2^ (50x), *b *= 2000 s/mm^2^ (50x), 105 + 6 time-points (PA-AP).
- **Resting-state fMRI (rsfMRI)**: 2.4 mm isotropic resolution, flip angle = 52 degrees, fat saturation, TE = 39 ms, TR = 735 ms, scan duration = 6 min.

#### Structural MRI feature selection

Structural similarity necessarily depends on the measurements of brain structure on which it is based. We previously showed that multivariate networks are both more stable and more aligned with benchmarks of biological validity, and that multivariate MIND network architecture is not disproportionately influenced by a single feature^25^. On this basis, we estimated similarity based on a diverse set of interpretable macro- and micro-structural MRI features, and aimed to select these features in a principled manner. Here, we estimated similarity based on a diverse set of interpretable macro- and micro-structural MRI features. We considered a total possible set of 13 structural MRI phenotypes which have previously been genetically analysed in a recent GWAS study^20^: these included expansion-related phenotypes (e.g., surface area; SA, volume; Vol), cortical thickness (CT), curvature phenotypes (mean curvature; MC, Gaussian curvature; GC), standard diffusion-based phenotypes (mean diffusivity; MD, fractional anisotropy; FA) and neurite orientation dispersion and density imaging (NODDI) features from diffusion-weighted imaging (orientation dispersion index; ODI, isotropic volume fraction; ISOVF, and neurite density index; NDI, also known as intracellular-volume fraction; ICVF). From among these 13 phenotypes, we selected a final set of four MRI features as the basis for MIND similarity network estimation, each of which satisfied the following three criteria:

- **Local (vertex-level) interpretability**. MIND calculates structural similarity by comparing regional distributions of structural features measured at the level of individual vertices on the cortical surface. Therefore, we only considered features that were well-defined at the level of the individual vertex. For this reason, we excluded all expansion-based phenotypes, including surface area and volume, which are only well-defined by aggregating (summing) information across vertices. Measured at the vertex level, these measurements are a direct function of the size and number of vertices used to reconstruct the surface mesh. Cortical thickness, curvature-based phenotypes, and diffusion-based phenotypes passed this criterion.
- **Genetic complementarity**. We aimed to integrate features capturing diverse genetic signals while avoiding the inclusion of multiple features that capture redundant signal. Reference ^20^ calculated the genetic correlations between each of the 13 globally-measured phenotypes and identified five genetically-defined clusters of structural features, representing: (1) expansion, (2) curvature, (3) thickness, (4) neurite density and orientation, and (5) water diffusion. To minimise redundancy while maximising signal diversity, we included one feature from each cluster (except the expansion-related cluster excluded by the interpretability criterion).
- **SNP-based heritability**. CT was the only representative of the thickness cluster and had high heritability (*h*^2^ = 0.3). For each of the other three clusters, we selected a representative feature primarily by maximising SNP-based heritability. For this reason, from the cluster of curvature phenotypes (MC and GC), we selected MC (*h*^2 ^= 0.33 vs *h*^2 ^= 0.19 for GC), and from the cluster of neurite density and orientation phenotypes (NDI, FA, and ODI), we selected NDI (*h*^2 ^= 0.17 vs *h*^2 ^= 0.11 for FA and *h*^2 ^= 0.07 for ODI). The only exception to this preference for the highest heritability feature was for the water diffusion cluster (MD and ISOVF), where we selected MD (*h*^2 ^= 0.15) over ISOVF (*h*^2 ^= 0.24) as MD is more widely used in the neuroimaging community.

We settled on two macro-structural MRI phenotypes (mean curvature, MC; cortical thickness, CT) and two micro-structural phenotypes (mean diffusivity, MD; neurite density index, NDI). MC and CT were automatically defined at the vertex-level via default FreeSurfer outputs. MD and NDI were mapped from volumetric space (voxel resolution) to the vertex-level resolution of the cortical mesh via a surface-based registration in line with Human Connectome Project protocols, as described in ref. ^20^. The ${h}_{SNP}^{2}$ of the final four selected features are reported in Supplementary Fig. 4A.

#### MIND network construction

For each subject, the four MRI features were measured at every vertex on the subject-specific cortical surface mesh reconstruction, and aggregated over all vertices in each parcel of the widely-used 360-region HCP parcellation^46^, such that every region was defined by a four-dimensional distribution of the vertices contained within it. The similarity between each pair of these multivariate distributions was then estimated by morphometric inverse divergence (MIND)^25^, which is the inverse of the Kullback-Leibler divergence between areal distributions, for all possible pairs of areas, resulting in a single-subject {360 region x 360 region} structural similarity network. However, each of these networks contained 64,620 edges, which was too many to be tractable for edge-level GWAS analysis. We therefore coarse-grained the {360 region x 360 region} networks to {23 area x 23 area} networks comprising 276 edges for GWAS analysis, as illustrated in Figure 1. To perform this coarse-graining step, we leveraged the fact that the original HCP parcellation was derived in a hierarchical manner such that each of the 360 regions was assigned to one of 22 higher-order cortical systems. Given the known cytoarchitectonic difference between primary motor cortex and primary somatosensory cortex^84^, we additionally split the higher-order sensorimotor system into its component motor and somatosensory cortical areas, leading to a total of 23 systems to which each of the 360 parcellated regions were assigned. Similarities within and between each of the 23 cortical systems were averaged for each participant, leading to the construction of subject-specific {23 area x 23 area} MIND networks.

#### Functional connectivity estimation

Generating FC matrices from raw fMRI data involved three main steps: fMRI preprocessing, fMRI parcellation, and band-pass filtering. The first processing step was previously performed by the UKB and is described here (https://biobank.ctsu.ox.ac.uk/crystal/crystal/docs/brain_mri.pdf). Briefly, it includes motion correction using MCFLIRT^85^; grand-mean intensity normalisation of the entire 4D dataset by a single multiplicative factor; highpass temporal filtering (Gaussian-weighted least-squares straight line fitting, with sigma = 50.0s); EPI unwarping; GDC unwarping; and structured artefact removal. The HCP parcellation was transformed from the fsaverage standardised space to native space using surface-based non-linear registration. fMRI was linearly co-registered to the T1 image using FLIRT^86^. The resulting inverse transformation was used to map the T1-based parcellation into the fMRI space for the extraction of the average time series of each parcel. Finally, we focused on the physiologically relevant frequency range by using wavelet filtering that retained the BOLD oscillations in the frequency range 0.01–0.10 Hz (wavelet scales 2, 3 and 4).

### Genome-wide association analysis

For the primary GWAS on MIND network edges, in order to mitigate the effect of technical outliers, subjects were additionally excluded if they had values  ± 5 standard deviations from the mean across any of the 276 MIND network edges, leading to a maximum of 30,524 participants. The same imaging inclusion criteria was applied to the replication cohort leading to a maximum of 18,769 subjects in the validation cohort.

For the subsequent gradient-level GWAS, subjects were also removed if they were  ± 5 standard deviations from the mean subject-specific values of G1 and G2, leading to a maximum of 30,627 subjects in the discovery cohort. The same procedure for outlier control was applied to the corresponding GWAS of edges and gradients in the FC network, leading to a maximum of 30,376 and 29,715 participants, respectively, for these two phenotypic levels of GWAS.

In total, we conducted a total of 276 × 2 (MIND and FC edges) + 2 × 2 (MIND and FC gradients) = 556 genome-wide association studies (in the discovery cohort). In all GWAS, as in ref. ^20^, we standardised each phenotype to have a mean of zero and standard deviation of 1. We conducted all GWAS using FastGWA^87^ and as previously described in ref. ^20^ we incorporated the following covariates: age (years), age^2^, sex, age × sex, age^2^ × sex, the first 40 genetic PCs as provided by the UKB, both mean and maximum framewise displacement (FD) corresponding to motion in the rs-fMRI scan, Euler Index, dummy variables encoding imaging centre, and a dummy variable indicating whether T2 images were available for each subject, which may affect cortical surface reconstruction. After running edge-level GWAS, independent loci were identified via two-stage clumping using FUMA^88^ with *P* < 0.05 at a threshold of *α* = 1.8*e* − 10 and default parameters of *r*^2 ^= 0.6 and *r*^2^=  0.1. The resulting loci were then merged if they fell within 250 kb of each other, thus reducing the number of loci from 69 to the final number of 54 reported in the main text. The same process was used to find independent loci for gradient-level GWAS, but with a threshold of *α* = 5*e* − 08.

### Genetic correlations and causal analysis

To calculate the pairwise genetic correlations between MIND network edges, we used LD Score Regression^47^, with HapMap3 SNPs^89^ and LD scores pre-computed from the 1KG European reference panel^90^, due to its widespread use and fast runtime, which enabled 276*(276-1)=75,900 total genetic correlations needed to construct the pairwise genetic correlation matrix. Genetic correlations between MIND and FC were computed primarily using High-Definition Likelihood (HDL), and their precomputed reference panel of 1,029,876 quality-controlled UK Biobank imputed HapMap3 SNPs^91^. This is more computationally expensive but has been shown to be a more powerful approach that yields comparatively more precise estimates of genetic correlations, especially when SNP-heritabilities are low, as is the case with edge-level functional connectivity (Fig. 4C). Genetic correlations were compared with those derived from LD Score Regression and were highly consistent (*r* = 0.65; paired *t* test *P* > 0.05). SNP-based heritabilities for all MIND and FC phenotypes were additionally computed using HDL^91^. Local genetic correlations between MIND and FC were computed using SUPERGNOVA^76^. Quasi-independent genomic regions were estimated and provided by the original SUPERGNOVA software. Colocalisation between MIND and FC was performed with coloc with default priors of *p*_1_ = 1*e* − 04, *p*_2_ = 1*e* − 04, *p*_12_ = 1*e* − 05 ^92,93^.

Mendelian randomisation was conducted primarily using MR-APSS, a method which accounts for sample structure, including sample overlap, and increases power through the inclusion of more genetic instruments without increased false positive rate^62^. This method was particularly suitable for our analysis, given a) sample overlap in the GWAS performed for FC and MIND, and b) few genetic instruments available at the standard significance threshold (*P* < 5*e* − 08) for many edges. Here we used an instrument-level cutoff of *P* < 5*e* − 04, which falls within the recommended range^62^. In a recent benchmarking study of 16 MR methods, MR-APSS was the top-performing method in terms of statistical power, type 1 error, and robustness to genetic instrument selection^63^ across all simulation scenarios. Nevertheless, we additionally performed several replication and sensitivity analyses of our main MIND-FC MR analysis including a) replicating the effect of G1 on FC1 using four standard two-sample MR methods (Inverse-Variance Weighted MR, Simple Median, Weighted Median, and MR-Egger) using MIND G1 (exposure) summary statistics derived from both the discovery and (fully non-overlapping) validation cohorts (Supplementary Fig. 7), b) testing robustness to MR-APSS model assumptions (Supplementary Fig. 6C) and c) replicating the original MR-APSS results using a threshold of *P* < 5*e* − 05 (see Supplementary Fig. 6D). Finally, we again used MR-APSS with an instrument-level cutoff of *P* < 5*e* − 04 for our analysis of causality between MIND G1 and G2 and the 9 biological traits analysed for which we observed significant genetic correlations (results of sensitivity analyses of robustness to varying instrument thresholds are reported in Table 1).

To estimate the genetic parameters of polygenicity (*π*) and negative selection (*S*), we used SBayesS software from the GCTB toolkit^50,51^ with the same parameters as ref. ^7^, including using the default ‘Sparse matrix (including MHC regions)’ as the LD matrix, and the flags –chain-length 10000 –thin 10 –burn-in 2000.

### Genetic gradient estimation

After the {276 edge x 276 edge} genetic correlation matrix *G* was compiled, we identified the first five genetic gradients using gradient decomposition as implemented by the BrainSpace package^48^, using the code parameters GradientMaps(kernel = 'normalized_angle', approach = 'dm'), and fit without any thresholding. The eigenvalues returned by diffusion mapping cannot be directly used to calculate the variance explained in the original data; using the approximation described by ref. ^49^, the first two gradients (G1 and G2) explained 53.7% and 17.4% of variance in *G*. To ensure that these gradients were robust to the matrix decomposition methodology, we repeated the process using principal component analysis (PCA). Replicating the results from the gradient decomposition, the first two principal components explained 54.4% and 17.7% of the variance in the matrix, respectively, and were almost perfectly correlated with G1 and G2 (*r*_*s*_ > 0.99 in both cases).

To map the network-level gradients to regional scores, we performed a second gradient decomposition of each of G1 and G2 using the same method. These regional scores were then compared to distance maps from the paleocortex, the archicortex, or any of the 180 (bilaterally-averaged) regions from the HCP parcellation. Each distance map was originally defined as the distance to each of the 180 bilaterally-averaged regions in the HCP parcellation, and was then coarse-grained via averaging within each of the 23 higher-order regions to facilitate a direct comparison to the regional gradient scores. For cortical areas from the HCP parcellation, distance matrices were calculated for left and right hemispheres independently, then averaged, using Connectome Workbench^94^. The same method of spatial association was used to compare regional G1 and G2 scores with the genetic principal components and other brain maps analysed in Supplementary Fig. 4.

### Statistical hypothesis testing

For GWAS-level tests, we used *P* < 5*e* − 08 (standard genome-wide significance). For edge-level tests, we defined experiment-wide significance at *P* < 1.8*e* − 10 (5*e* − 08/276 edges). For gradient-level tests, we defined experiment-wide significance at *P* < 2.5*e* − 08 (5*e* − 08/2 gradients). These Bonferroni corrections follow standard practice for neuroimaging-genetics studies^20^. All other tests used false discovery rate (FDR) correction at 5%, unless otherwise noted, and were corrected for the number of tests specific to each analysis unless otherwise noted, and were corrected for an analysis-specific number of tests. These FDR tests are appropriate for correlated tests where Bonferroni would be overly conservative. *P*-values from follow-up Mendelian Randomisation analyses were not corrected for multiple comparisons (Supplementary Table 1).

### Reporting summary

Further information on research design is available in the Nature Portfolio Reporting Summary linked to this article.

## Supplementary information

Supplementary Information

Reporting Summary

Transparent Peer Review file

## Source data

Source Data

## Data Availability

All summary statistics generated in this study are publicly available at 10.17863/CAM.128272. These include summary statistics for the 276 network edges and 2 genetic gradients for both MIND and FC phenotypes. Summary statistics for schizophrenia (SCZ), major depressive disorder (MDD), attention-deficit hyperactivity disorder (ADHD), autism spectrum disorder (ASD), bipolar disorder (BPD), and Alzheimer’s disease (ALZ) can be downloaded from https://pgc.unc.edu/for-researchers/download-results/^95–100^. Summary statistics for C-reactive protein concentration (CRP), Body Mass Index (BMI) and gestational duration (GEST) can be downloaded, respectively, from https://ftp.ebi.ac.uk/pub/databases/gwas/summary_statistics/GCST90029001-GCST90030000/GCST90029070/, https://portals.broadinstitute.org/collaboration/giant/index.php/GIANT_consortium_data_files#2018_GIANT_and_UK_BioBank_Meta-analysis, and https://egg-consortium.org/gestational-duration-2019.html^101–104^. Further information for each of the summary statistics is provided in Supplementary Table 2. Paleocortical and archicortical distance maps were downloaded from ref. ^56^. The maps of the first genetic principal components of surface area, cortical thickness, mean diffusivity, mean curvature, and intracellular volume fraction were accessed from ref. ^20^. The first principal component of the T1w/T2w microstructural profile covariance matrix was downloaded from ref. ^105^. Brain maps of allometric scaling, evolutionary expansion, and externopyramidisation were downloaded from ref. ^106^. Source data are provided in this paper.

## Code availability

Code to calculate MIND networks can be accessed at 10.5281/zenodo.7974716. FastGWA was conducted with GCTA software v.1.94.1^107^. Colocalisation was performed using coloc v5.2.3 and R v4.3.1. Genal was used for for MR sensitivity analysis^108^. Both plink v1.9 and 2.0 were used in this study^109^. Gradient decomposition was performed using the BrainSpace Python package v0.1.10^48^. Data analysis and visualisation was conducted using Python 3.6, 3.9, and 3.11. Locus plots were created using https://github.com/jrs95/geni.plots. Brain network plots were created using NetPlotBrain v0.3.0^110^. UpSet plots were created using the code from ref. ^111^. Phenogram chromosome maps were created using the software at https://visualization.ritchielab.org/phenograms/plot.
