# Supplementary material for: The genetic architecture of cortical similarity networks
Source: Nat Commun. 2026 Jun 20;17:7801. doi: 10.1038/s41467-026-73714-9 (PMC13438111; doi:10.1038/s41467-026-73714-9)
Supplement: Supplementary file 1 [file 41467_2026_73714_MOESM1_ESM.pdf]

Supplementary Information

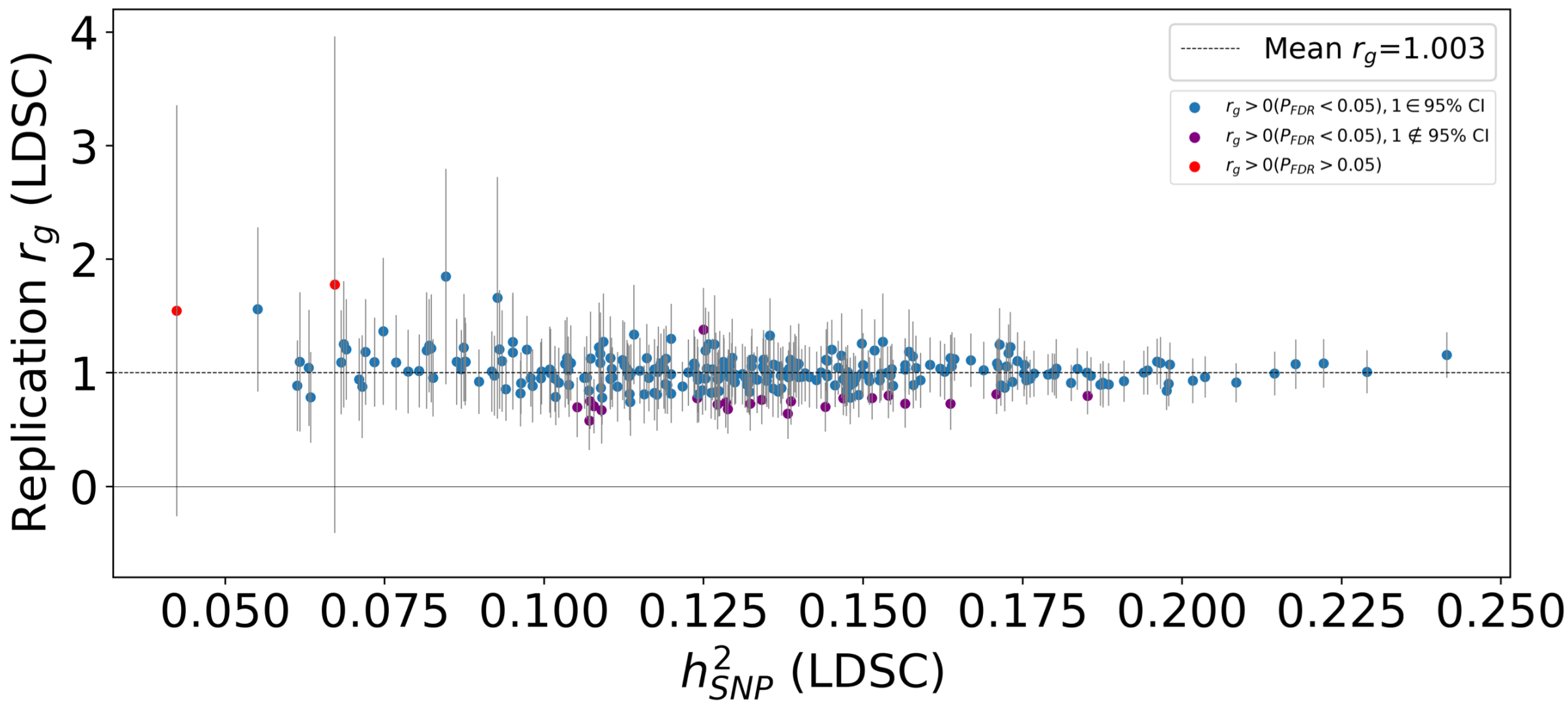

Supplementary Fig. 1 Replication of primary edge-level MIND GWAS. Scatterplot indicating the (LDSC) genetic correlation between the primary discovery GWAS, performed in $N = 30,524$ subjects from the UK Biobank, and the replication GWAS from an additional $N = 18,769$ from the UK biobank. The split between discovery and validation datasets naturally occurred due to data availability – data from the additional replication subjects became available in 2024, after the original study began in 2023. Each point in the scatterplot indicates one of the 276 total MIND network edges, with the x-axis representing its SNP-based heritability (LDSC-derived) and the y-axis showing the estimated genetic correlation between discovery and validation datasets, with 95% confidence intervals shown. The mean genetic correlation is shown as a dashed line (at $r_g = 1.003$). All but 274 of 276 edges had significantly positive genetic correlations across replication and discovery datasets, with two edges not reaching significance, demonstrating low heritability, shown in red. 254 of 276 edges (92%) had genetic correlations with confidence intervals overlapping with $r_g = 1$; the 22 edges where this was not the case are indicated in purple.

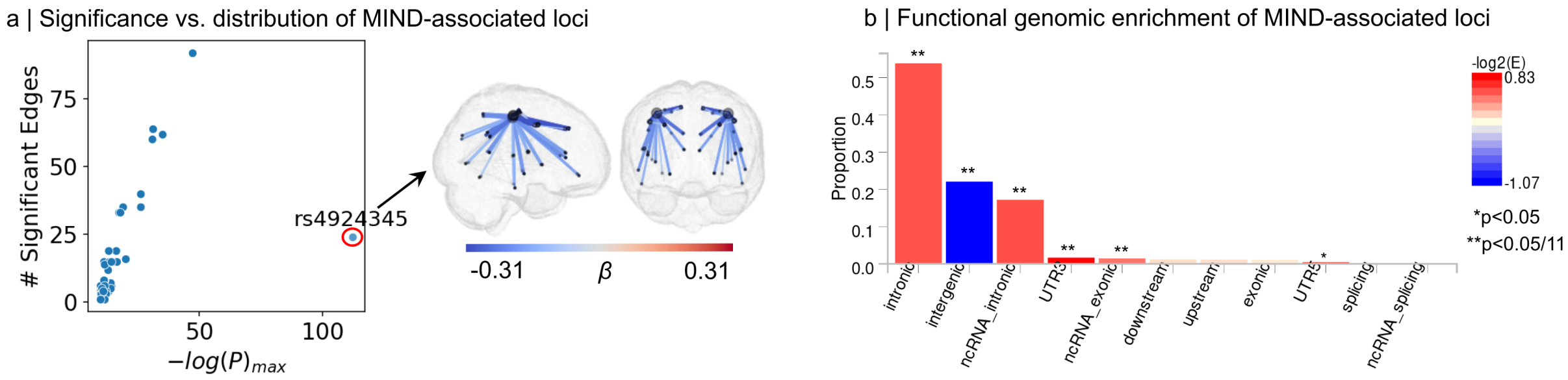

Supplementary Fig. 2 Distribution and enrichment of loci associated with MIND network edges. A) The left plot shows a scatterplot of the relationship between the maximum significance achieved by a given variant across all MIND network edges, and the number of edges at which it achieves significance. Each point represents the index variants for each of the 54 loci associated with at least one MIND network edge at an experiment-wide threshold of $P < 1.8e-10$. On average, the relationship between the maximum significance achieved and number of significance was highly positive, pointing to a strong general tendency for distributed network effects of MIND-associated loci. The one major outlier to this pattern was *rs4924345*; Occurring at chr15:39,639,898 (shown on the Manhattan plot in Fig. 2), this locus achieved the highest significance with MIND, but was associated with comparably few network edges. The correlation of maximum $-log_{10}(P)$ with the number of significantly-associated edges was $r_s = 0.81$. The brain plot on the right shows which edges were significantly associated with *rs4924345* at $P < 5e-08$; as shown, they are exclusively focused on the somatosensory cortex. This result is consistent with previous findings showing associations with this variant and central sulcus morphology across multiple imaging-genetics datasets (van der Meer et al, 2021; Sun et al, 2022). B) Functional genomic enrichment plots computed by FUMA using ANNOVAR for 'min-P' values of genetic associations with MIND network edge connectivity across all 276 network edges. (Wang et al, 2010; Watanabe et al, 2017).

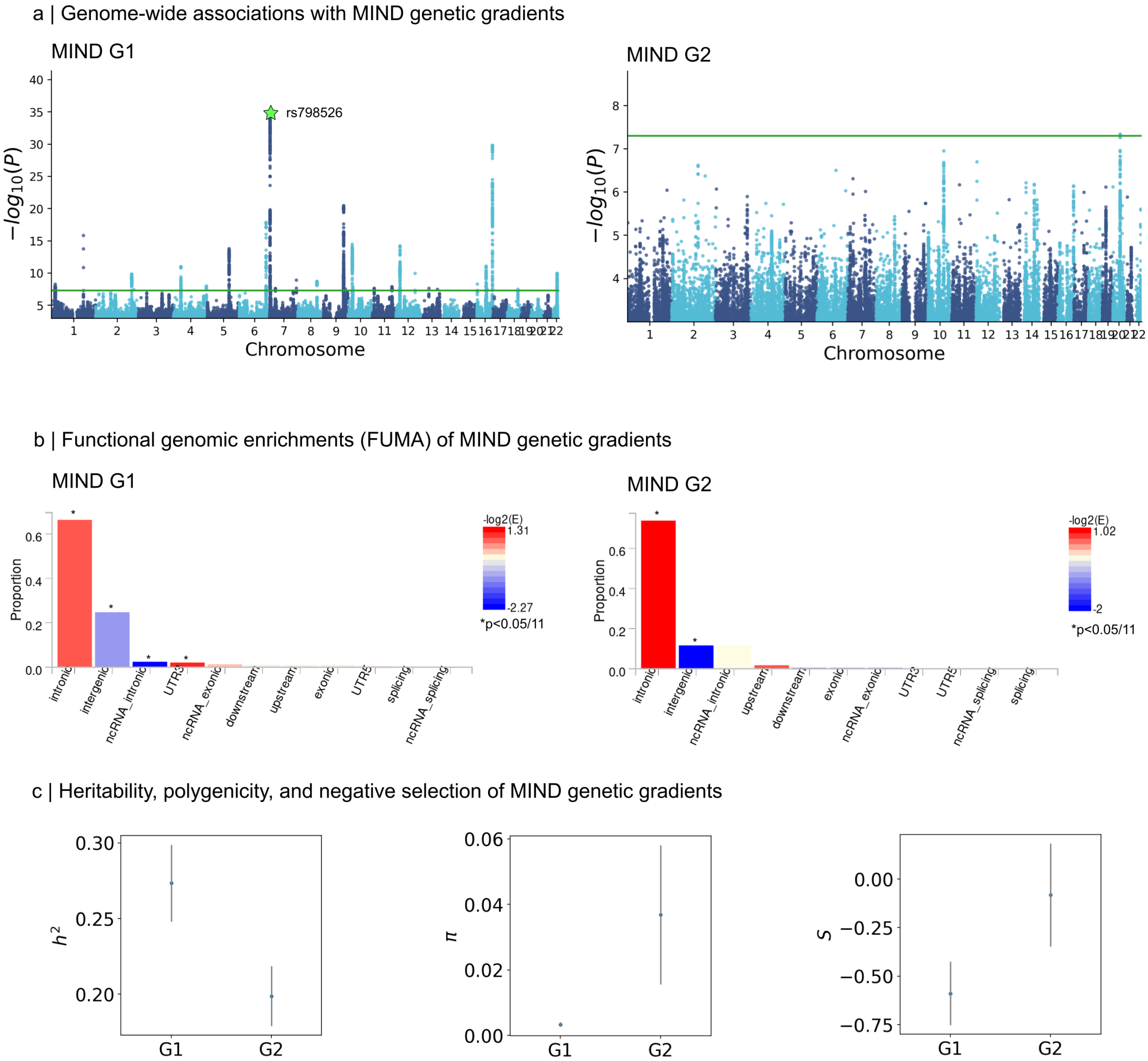

Supplementary Fig. 3 Gradient-level MIND genetic associations and enrichments. A) Manhattan plots showing the genetic associations with MIND, summarized via dot product with the edge-level weights of G1 and G2. The green line represents genome-wide significance of $P < 5e-08$. In panel A, the green star highlights the top G1-associated variant, *rs798526*. B) Functional genomic enrichment plots computed by FUMA using ANNOVAR for summary statistics of MIND G1 and MIND G2 (Wang et al, 2010; Watanabe et al, 2017). C) Estimates of SNP-based heritability, polygenicity parameter π, and negative selection parameter S of the two principal MIND genetic gradients G1 and G2, calculated using SBayesS (Zeng et al, 2018). Shaded lines indicated 95% confidence intervals.

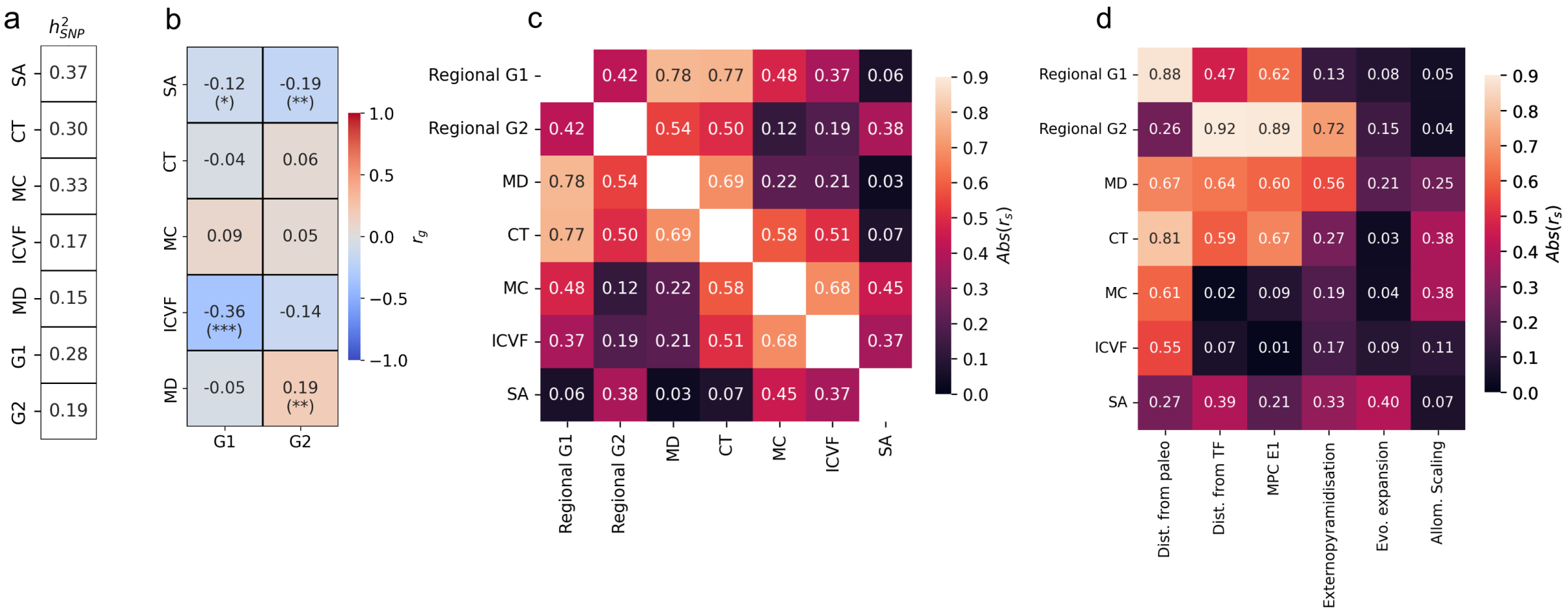

Supplementary Fig. 4 Genetic and spatial relationships between MIND gradients and univariate global measures of brain structure. A) The SNP heritabilities of MIND G1/G2 and global measures of brain structure. MIND phenotype heritabilities were calculated using LDSC, and all other heritabilities were as reported in Warrier et al (2023). SA: surface area, ICVF: intracellular volume fraction, CT: cortical thickness, MC: mean curvature, MD: mean diffusivity. B) The genetic correlation (LDSC) between MIND G1/G2 and global measures of brain structure, using publicly-available summary statistics from Warrier et al (2023). P-values were FDR corrected: $^{*} < 0.01 < P < 0.05$, $^{**}0.001 < P < 0.01$, $^{***}P < 0.001$. C) The spatial relationships (absolute Spearman correlation) between MIND G1 and G2 regional scores and the first principal components of regional genetic similarity matrices SA, CT, MC, ICVF, and MD, from Warrier et al (2023). D) The spatial relationships (absolute Spearman correlation) of MIND G1 and G2 regional scores, and the principal genetic components from part (C), with six cortical maps indexing complementary aspects of dual origin theory, brain evolution, microstructure, and areal scaling. These include (1) the distance from paleocortex and (2) distance from area TF (the two dual-origin geodesic distance maps discussed in Fig. 3), (3) the principal gradient of the T1w/T2w microstructural profile covariance network (MPC E1), reflecting laminar myeloarchitectural complexity (Paquola et al, 2020), (4) cortical externopyramidisation, a measure of laminar cytoarchitectural differentiation (Amunts et al, 2013; Sydnor et al, 2021), (5) evolutionary expansion (Sydnor et al, 2021), and (6) allometric scaling (Sydnor et al, 2021).

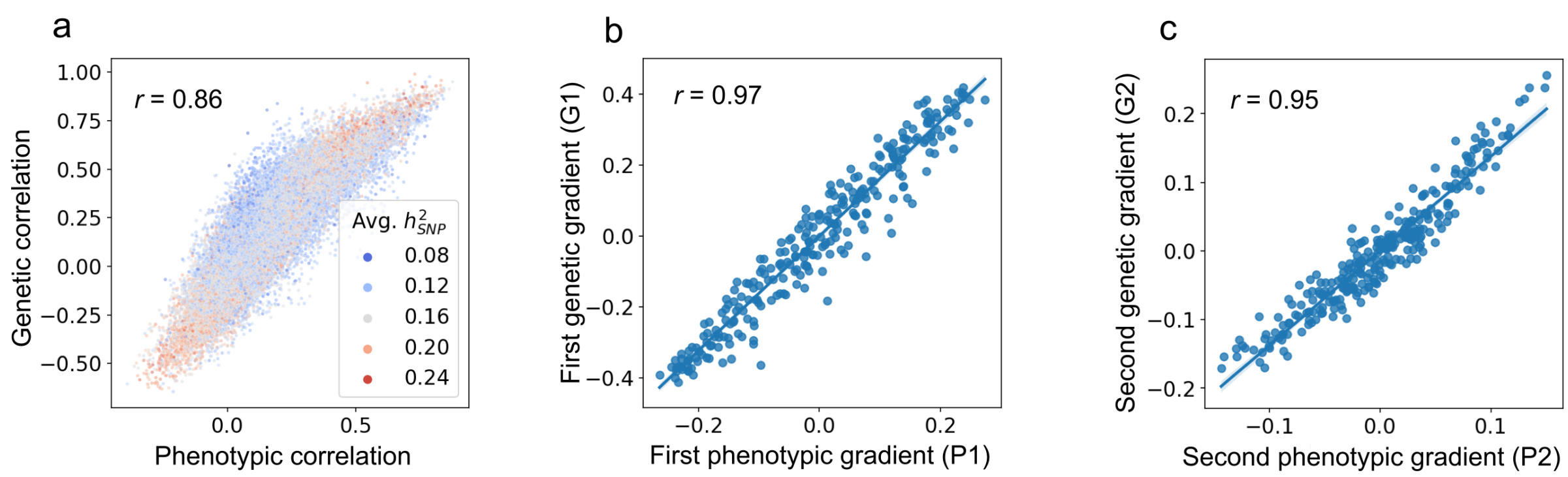

Supplementary Fig. 5 Genetic vs. phenotypic MIND network edge relationships. A) The association between phenotypic and genetic correlation between MIND network edges. Each point in the scatterplot represents a pair of edges. B-C) The first two phenotypic gradients (P1 and P2) defined by gradient decomposition of the edge-by-edge phenotypic correlation matrix (P) were highly similar to the first two genetic gradients (G1 and G2) derived from the edge-by-edge genetic correlation matrix (G).

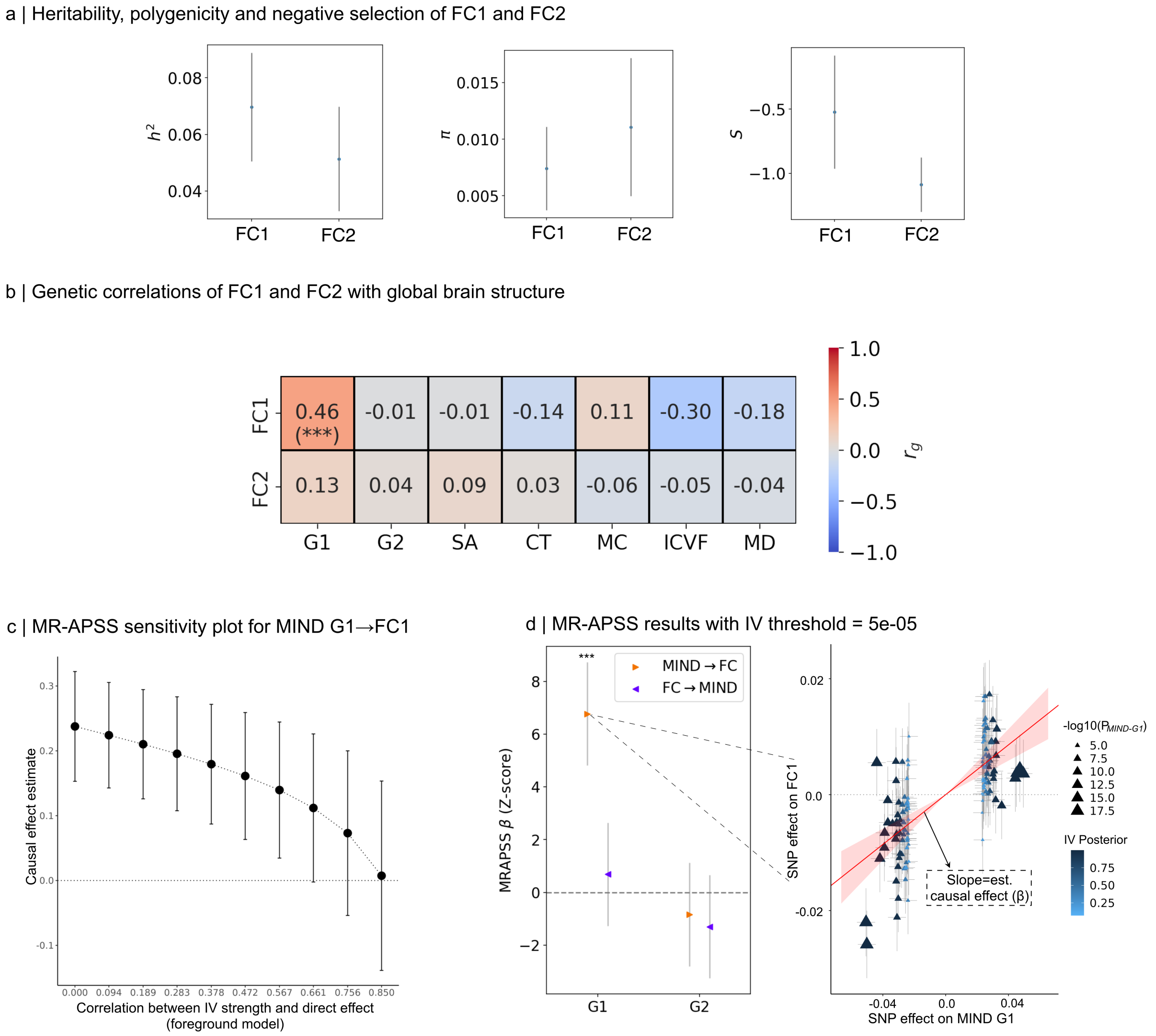

Supplementary Fig. 6 Genetic relationships between FC, global brain structure, and MIND gradients. A) Estimates of SNP-based heritability, polygenicity parameter π, and negative selection parameter S of fMRI-derived functional connectivity (FC) summarised along the two principal MIND genetic gradients (labeled FC1 and FC2). All estimates were calculated using SBayesS (Zeng et al, 2018). Shaded lines indicated 95% confidence intervals. B) The genetic correlation (LDSC) between FC1 and FC2 with MIND G1, MIND G2, and five univariate global measures of brain structure, using publicly-available summary statistics from Warrier et al (2023). SA: surface area, ICVF: intracellular volume fraction, CT: cortical thickness, MC: mean curvature, MD: mean diffusivity. P-values were FDR corrected: $* < 0.01 < P < 0.05$, $** 0.001 < P < 0.01$, $*** P < 0.001$. C) Sensitivity plot for the result shown in Figure 4E showing a significant causal estimate of MIND G1 on FC1. This plot tests the robustness of the result to model assumptions about the relationship between instrumental variable (IV) strength and the direct causal effect in the foreground model, which is assumed to be zero (Hu et al, 2022). The plot shows here that the positive causal effect of MIND on FC along G1 result remains significant as long as this value remains below 0.661. To put this number in context, the result remains significant for over twice as large a parameter space as the causal evidence for body mass index (BMI) on Type II diabetes, a widely-accepted causal relationship which was previously shown to be robust for values below 0.247 (Hu et al, 2022). D) A replication of the gradient-level MR-APSS results shown in Figure 4E, but with an IV threshold of $5e-05$ instead of $5e-04$. The left plot shows estimated (forward and reverse) causal relationships between MIND and FC (with 95% CI) separately for both G1 and G2. The right plot shows the highly significant causal effect of G1 on FC1, which is shown by the significantly positive slope of the regression plot (Z-scored β=6.2, $P = 6.0$e-10).

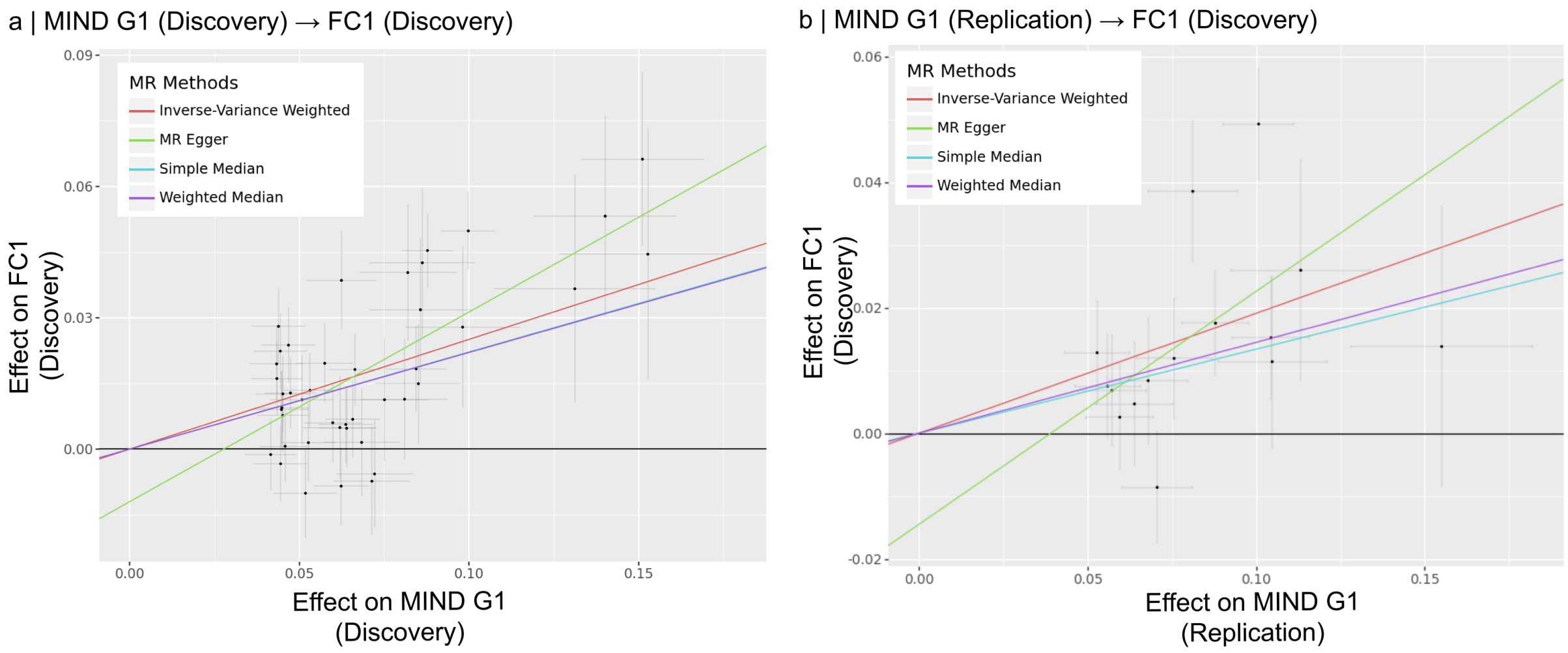

Supplementary Fig. 7 Out-of-sample replication of the causal effect of MIND on FC along G1. Results from four standard MR methods for estimating the causal effect of MIND on FC along G1, computed using MIND G1 summary statistics from the A) discovery cohort ($N_{max} = 30,627$) and B) replication cohort ($N_{max} = 18,743$). All results used the summary statistics for FC along G1 (FC1) from the discovery cohort. Thus, part B is a replication of these results using fully non-overlapping samples. Genetic instruments in both cases were selected using the standard strict threshold of $P < 5e-08$. A) In this within-sample analysis, all four methods exhibited significant positive causal effects, with exact values as follows: IVW; $\beta = 0.25, P = 7.5e-15$, MR Egger; $\beta = 0.43, P = 1.3e-04$, Simple Median; $\beta = 0.22, P = 8.2e-08$, Weighted Median; $\beta = 0.22, P = 1.6e-07$. The Egger intercept was not significantly different from zero (-0.01, $P = 0.07$). B) Replicating these results in a strict two-sample framework with no subject overlap, all four methods again showed significant positive causal effects, with exact values as follows: IVW; $\beta = 0.19, P = 1.3e-05$, MR Egger; $\beta = 0.37, P = 4.4e-02$, Simple Median; $\beta = 0.13, P = 7.9e-03$, Weighted Median; $\beta = 0.14, P = 1.7e-03$. The Egger intercept was again not significantly different from zero (-0.01, $P = 0.28$).

		IV threshold = 5e-04		IV threshold = 5e-05		
Exposure	Outcome	β	P-val	β	P-val	P<0.05 across thresholds
G1	SCZ	0.042	2.0594e-02	0.037	4.3817e-02	✓
G2	SCZ	-0.13	1.6887e-01	-0.149	2.8469e-01	X
G2	MDD	0.02	4.1479e-01	0.0489	3.7179e-01	X
G2	ADHD	-0.1054	4.6227e-02	-0.146	1.4359e-01	X
G2	CRP	-0.1099	2.4593e-01	-0.135	3.4173e-01	X
G2	BMI	-0.0284	7.0446e-01	0.056	3.6625e-01	X
SCZ	G1	-0.004	9.0280e-01	-0.0018	9.6114e-01	X
SCZ	G2	-0.067	9.3939e-02	-0.078	8.4111e-02	X
MDD	G2	-0.259	3.1982e-02	-0.175	2.9114e-01	X
ADHD	G2	-0.178	1.2060e-01	-0.210	1.6264e-01	X
CRP	G2	-0.081	2.6970e-02	-0.076	4.4732e-02	✓
BMI	G2	-0.145	5.7496e-04	-0.151	7.9968e-04	✓

Supplementary Table 1 Mendelian Randomization between MIND gradients and biological outcomes. Mendelian Randomization results for the six significant genetic correlations observed between G1 or G2 and the 9 biological outcomes considered, as indicated by elements of the r_g matrix in Figure 5A (and excluding FC phenotypes). Estimates are derived using MR-APSS using different P-value thresholds for instrumental variable (IV) selection. Reported P-values were computed by MR-APSS at the indicated IV threshold and were uncorrected across IV thresholds or comparisons.

	Reference	Website	File	N_{tot}	N_{ca}	N_{co}	N_{eff}
MDD	Wray et al (2018)	https://pgc.unc.edu/	daner_pgc_mdd_meta_w2_no23andMe_rmUKBB.gz	142646	45396	97250	61898
SCZ	Trubetskoy et al (2022)	https://pgc.unc.edu/	PGC3_SCZ_wave3.european.autosome.public.v3.tsv.gz	130644	53386	77258	58749
ADHD	Demontis et al (2023)	https://pgc.unc.edu/	ADHD2022_iPSYCH_deCODE_PGC.meta.gz	292548	38691	275986	67867
ASD	Grove et al (2019)	https://pgc.unc.edu/	iPSYCH-PGC_ASD_Nov2017.gz	46351	18,382	27,969	22184
BPD	Mullins et al (2021)	https://pgc.unc.edu/	daner_bip_pgc3_nm_noukbiobank.gz	371549	41917	371549	75334
ALZ	Wightman et al (2021)	https://pgc.unc.edu/	PGCALZ2ExcludingUKBand23andME_METAL_InverseVariance_MetaAnalysis.txt	398058	-	-	113888
CRP	Said et al (2022)	https://ftp.ebi.ac.uk/pub/databases/gwas/summary_statistics/	GCST90029070_buildGRCh37.tsv.gz	575,531	-	-	-
BMI	Yengo et al (2018); Locke et al (2015)	https://portals.broadinstitute.org/collaboration/giant/index.php/GIANT_consortium_data_files#2018_GIANT_and_UK_BioBank_Meta-analysis	Meta-analysis_Locke_et_al+UKBiobank_2018_UPDATED.txt	795640	-	-	-
GEST	Liu et al (2019)	https://egg-consortium.org/	Fetal_gest_duration_NComms2019.txt.gz	84,689	-	-	-

Supplementary Table 2 External summary statistics accessed and used in this study. ALZ: Alzheimer's, GEST: gestational age at birth, SCZ: schizophrenia, MDD; major depressive disorder, BMI; body mass index, CRP; C-reactive protein levels, BPD; bipolar disorder, ADHD; attention-deficit hyperactivity disorder, ASD; autism spectrum disorder, N_{Ca}; number of cases, N_{Co}; number of controls, N_{Eff}; Effective sample size, N_{Tot}; Total sample size. For MDD, SCZ, ADHD, and ASD, N_{eff} was approximated as $2/(1/N_{Ca} + 1/N_{co})$ as described by Winkler et al (2014), with the maximum N_{Ca} and N_{Co} values over all SNPs used in the calculation. For these traits, N_{Tot} was calculated as these two maximum values summed together. For ALZ, N_{Ca} and N_{Co} were not provided in the summary stats files, so the precomputed maximum N_{eff} and N_{Tot} were used instead. For continuous traits, calculating N_{eff} was not applicable.
