# Supplementary material for: The genetic architecture of cortical similarity networks
Source: Nat Commun. 2026 Jun 20;17:7801. doi: 10.1038/s41467-026-73714-9 (PMC13438111; doi:10.1038/s41467-026-73714-9)
Supplement: Supplementary file 2 [file 41467_2026_73714_MOESM2_ESM.pdf]

nature portfolio

Corresponding author(s): Isaac Sebenius
Last updated by author(s): 3/18/2026

Reporting Summary

Nature Portfolio wishes to improve the reproducibility of the work that we publish. This form provides structure for consistency and transparency in reporting. For further information on Nature Portfolio policies, see our Editorial Policies and the Editorial Policy Checklist.

Statistics

For all statistical analyses, confirm that the following items are present in the figure legend, table legend, main text, or Methods section.

n/a	Confirmed	
☐	☒	The exact sample size (n) for each experimental group/condition, given as a discrete number and unit of measurement
☐	☒	A statement on whether measurements were taken from distinct samples or whether the same sample was measured repeatedly
☐	☒	The statistical test(s) used AND whether they are one- or two-sided<br>*Only common tests should be described solely by name; describe more complex techniques in the Methods section.*
☐	☒	A description of all covariates tested
☐	☒	A description of any assumptions or corrections, such as tests of normality and adjustment for multiple comparisons
☐	☒	A full description of the statistical parameters including central tendency (e.g. means) or other basic estimates (e.g. regression coefficient) AND variation (e.g. standard deviation) or associated estimates of uncertainty (e.g. confidence intervals)
☐	☒	For null hypothesis testing, the test statistic (e.g. F, t, r) with confidence intervals, effect sizes, degrees of freedom and P value noted<br>*Give P values as exact values whenever suitable.*
☒	☐	For Bayesian analysis, information on the choice of priors and Markov chain Monte Carlo settings
☒	☐	For hierarchical and complex designs, identification of the appropriate level for tests and full reporting of outcomes
☐	☒	Estimates of effect sizes (e.g. Cohen's d, Pearson's r), indicating how they were calculated

Our web collection on statistics for biologists contains articles on many of the points above.

Software and code

Policy information about availability of computer code

Data collection	N/A; no data was collected for this study and only public datasets were used.
Data analysis	Code to calculate MIND networks can be accessed at https://doi.org/10.5281/zenodo.7974716. FastGWA was conducted with GCTA software v.1.94.1 (Yang et al, 2011). Colocalisation was performed using coloc v5.2.3 and R v4.3.1. Genal was used for for MR sensitivity analysis (Rivier et al, 2024). Both plink v1.9 and 2.0 were used in this study (Purcell et al, 2007). Gradient decomposition was performed using BrainSpace python package v0.1.10 (Vos de Wael et al, 2020). Data analysis and visualization was conducted using Python 3.6, 3.9, and 3.11. Locus plots were created using https://github.com/jrs95/geni.plots. UpSet plots were created using the code from Lex et al (2014). Phenogram chromosome maps were created using the software at https://visualization.ritchielab.org/phenograms/plot. Brain network plots were created using NetPlotBrain v0.3.0 (Fanton and Thompson, 2023).

For manuscripts utilizing custom algorithms or software that are central to the research but not yet described in published literature, software must be made available to editors and reviewers. We strongly encourage code deposition in a community repository (e.g. GitHub). See the Nature Portfolio guidelines for submitting code & software for further information.

Data

Policy information about availability of data

All manuscripts must include a data availability statement. This statement should provide the following information, where applicable:

- Accession codes, unique identifiers, or web links for publicly available datasets
- A description of any restrictions on data availability
- For clinical datasets or third party data, please ensure that the statement adheres to our policy

We used data from 62,162 participants from the UK biobank in this study; access to this resource can be applied to at https://www.ukbiobank.ac.uk/enable-your-research/apply-for-access. All summary statistics generated in this study are publicly available at https://doi.org/10.17863/CAM.128272. These include summary statistics for the 276 network edges and 2 genetic gradients for both MIND and FC phenotypes. Summary statistics for schizophrenia (SCZ), major depressive disorder (MDD), attention-deficit hyperactivity disorder (ADHD), autism spectrum disorder (ASD), bipolar disorder (BPD), and Alzheimer's disease (ALZ) can be downloaded from https://pgc.unc.edu/for-researchers/download-results/ (Trubetskoy et al, 2022; Wray et al, 2018; Demontis et al, 2023; Grove et al, 2019; Mullins et al, 2021; Wightman et al, 2021). Summary statistics for C-reactive protein concentration (CRP), Body Mass Index (BMI) and gestational duration (GEST) can be downloaded, respectively, from https://ftp.ebi.ac.uk/pub/databases/gwas/summary statistics/GCST90029001-GCST90030000/GCST90029070/, https://portals.broadinstitute.org/collaboration/giant/index.php/GIANT_consortium_data_files#2018_GIANT_and_UK_BioBank_Meta-analysis, and https://egg-consortium.org/gestational-duration-2019.html (Liu et al, 2019; Yengo et al, 2018; Locke et al, 2015; Said et al, 2022). Further information for each of the summary statistics is provided in Supplementary Table A1. Paleocortical and archicortical distance maps were downloaded from (Meng et al, 2022).

Research involving human participants, their data, or biological material

Policy information about studies with human participants or human data. See also policy information about sex, gender (identity/presentation), and sexual orientation and race, ethnicity and racism.

Reporting on sex and gender	We use sex in our manuscript to refer to a biological attribute, as reported by the UKB. We use sex as a covariate analysis in our GWAS in line with standard practice in the imaging-genetics literature (e.g. Warrier et al., 2023).
Reporting on race, ethnicity, or other socially relevant groupings	We do not directly study race, ethnicity, or other socially relevant groupings in our analysis. We use genetic principal components, which relate to ancestry, in our GWAS analysis to mitigate the effects of population stratification, as is standard practice in GWAS (e.g. Warrier et al., 2023).
Population characteristics	The UK Biobank contains data for a representative population sample of adults in the UK (>30 years of age).
Recruitment	N/A
Ethics oversight	Ethical procedures for the UKB are controlled by the Ethics and Guidance Council (http://www.ukbiobank.ac.uk/ethics), and the study was conducted in accordance with the UKB Ethics and Governance Framework document (https://www.ukbiobank.ac.uk/media/0xsbmfmw/egf.pdf)

Note that full information on the approval of the study protocol must also be provided in the manuscript.

Field-specific reporting

Please select the one below that is the best fit for your research. If you are not sure, read the appropriate sections before making your selection.

[x] Life sciences [ ] Behavioural & social sciences [ ] Ecological, evolutionary & environmental sciences

For a reference copy of the document with all sections, see nature.com/documents/nr-reporting-summary-flat.pdf

Life sciences study design

All studies must disclose on these points even when the disclosure is negative.

Sample size	From the roughly 500,000 participants in the UK Biobank, our primary analysis was conducted using data from a total of 40,715 participants with magnetic resonance imaging (MRI) data available in 2023, of which 34,988 had the full set of MRI sequences and sufficient scan quality necessary to construct MIND networks. In 2024, the requisite MRI data for MIND network construction became available for an additional 21,447 UKB participants (used as a replication dataset), bringing the total sample size to 40,715 + 21,447 = 62,162.
Data exclusions	Of the discovery dataset, 34,988 had the full set of MRI sequences and sufficient scan quality necessary to construct MIND networks. This study used the UKB genetic data that was previously quality-controlled and imputed before access as described by Bycroft et al (2018), and used largely the same set of participants and genetic inclusion criteria as described in Warrier et al (2023). Specifically, participants were included based on the following genetic criteria: 1) self-identification as ethnically white, 2) within ± 5 standard deviations of the mean along the first two genetic principal components, 3) matched genetic and reported sex, and 4) no excessive heterozygosity as reported by UKB. We included all SNPs with minor allele frequency greater than 0.1%, leading to the analysis of a maximum of 13,587,992 SNPs and 31,544 participants passing genetic inclusion criteria for the primary GWAS. For the primary GWAS on MIND network edges, in order to mitigate the effect of technical outliers, subjects were additionally excluded if they had values ± 5 standard deviations from the mean across any of the 276 MIND network edges. For the subsequent gradient-level GWAS, subjects were also removed if they were ± 5 standard deviations from the mean subject-specific values of G1 and G2.

Replication	In 2024, the requisite MRI data for MIND network construction became available for an additional 21,447 UKB participants. We applied the same genetic inclusion criteria to this validation cohort as for the original discovery cohort, leading to a maximum of 18,769 participants available for the replication GWAS.
Randomization	N/A
Blinding	N/A

Reporting for specific materials, systems and methods

We require information from authors about some types of materials, experimental systems and methods used in many studies. Here, indicate whether each material, system or method listed is relevant to your study. If you are not sure if a list item applies to your research, read the appropriate section before selecting a response.

Materials & experimental systems

n/a	Involved in the study	
☒	☐	Antibodies
☒	☐	Eukaryotic cell lines
☒	☐	Palaeontology and archaeology
☒	☐	Animals and other organisms
☒	☐	Clinical data
☒	☐	Dual use research of concern
☒	☐	Plants

Methods

n/a	Involved in the study	
☒	☐	ChIP-seq
☒	☐	Flow cytometry
☐	☒	MRI-based neuroimaging

Plants

Seed stocks	N/A
Novel plant genotypes	N/A
Authentication	N/A

Magnetic resonance imaging

Experimental design

Design type	We used T1-weighted, diffusion-weighted, and resting-state functional MRI scans in the GWASs of UKB data.
Design specifications	Only resting-state conditions were accessed for functional MRI data; N/A for structural scans.
Behavioral performance measures	N/A

Acquisition

Imaging type(s)	We used T1-weighted, diffusion-weighted, and resting-state functional MRI scans in the GWASs of UKB data.
Field strength	3T for all imaging modalities
Sequence & imaging parameters	The full imaging protocol can be found at: https://www.fmrib.ox.ac.uk/ukbiobank/protocol/V4 23092014.pdf and https://biobank.ctsu.ox.ac.uk/crystal/crystal/docs/brain mri.pdf. In brief, the acquisition parameters for each type of data were the following, as described in Alfaro-Almagro et al (2018); Zhao et al (2022); Warrier et al (2023):<br>• T1-weighted structural imaging (T1-w): 1.0 mm isotropic resolution, TR = 2,000 ms, TE = 2.01 ms, TI = 880 ms, flip angle = 8 degrees.<br>• T2-weighted fluid-attenuated inversion recovery (T2-FLAIR): 1.0 × 1.0 × 1.1 mm resolution, TE = 395.0 ms, TR = 5,000 ms, TI = 1,800 ms.<br>• Diffusion-weighted imaging (DWI): 2.0 mm isotropic resolution, MB = 3, R = 1, TE = 92 ms, TR = 3,600 ms, PF = 6/8, fat saturation, b = 0 s/mm² (5x + 3x phase-encoding reversed), b = 1,000

s/mm² (50x), b = 2,000 s/mm² (50x), 105 + 6 time-points (PA–AP).
• Resting-state fMRI (rsfMRI): 2.4 mm isotropic resolution, flip angle = 52 degrees, fat saturation, TE = 39 ms, TR = 735 ms, scan duration = 6 min.

Area of acquisition: Whole brain

Diffusion MRI: [x] Used [ ] Not used

Parameters: Diffusion-weighted imaging (DWI): 2.0 mm isotropic resolution, MB = 3, R = 1, TE = 92 ms, TR = 3,600 ms, PF = 6/8, fat saturation, b = 0 s/mm² (5x + 3x phase-encoding reversed), b = 1,000 s/mm² (50x), b = 2,000 s/mm² (50x), 105 + 6 time-points (PA–AP). Full details provided in https://www.fmrib.ox.ac.uk/ukbiobank/protocol/V4 23092014.pdf and https://biobank.ctsu.ox.ac.uk/crystal/crystal/docs/brain mri.pdf.

Preprocessing

Preprocessing software: Structural MRI and diffusion MRI were processed identically as described in Warrier et al., (2023) using FreeSurfer preprocessing. For DWI data, mapping from volumetric space (voxel resolution) to the vertex-level resolution of the cortical mesh via surface-based registration was performed in line with Human Connectome Project protocols, as described in Warrier et al. (2023).

Normalization: Processing for the UKB MRI data is described in https://biobank.ctsu.ox.ac.uk/crystal/crystal/docs/brain mri.pdf. Normalization of the fMRI data is described in more detail in the following two answers. All features used for GWAS were standardized, and subjects with features with +/- 5 standard deviations away from the mean were excluded.

Normalization template: The HCP parcellation was transformed from fsaverage standardised space to native space using surface-based non-linear registration. fMRI was linearly co-registered to the T1 image using FLIRT (Jenkinson et al, 2012). The resulting inverse transformation was used to map the T1-based parcellation into the fMRI space for the extraction of the average time series of each parcel.

Noise and artifact removal: Briefly, the UKB protocol for fMRI processing includes motion correction using MCFLIRT (Jenkinson et al, 2002); grand-mean intensity normalisation of the entire 4D dataset by a single multiplicative factor; highpass temporal filtering (Gaussian-weighted least-squares straight line fitting, with sigma=50.0s); EPI unwarping; GDC unwarping; and structured artefact removal. We focused on the physiologically relevant frequency range by using wavelet filtering that retained the BOLD oscillations in the frequency range 0.01–0.10 Hz (wavelet scales 2, 3 and 4).

Volume censoring: UKB performed volume censoring before our accessing of the data.

Statistical modeling & inference

Model type and settings: N/A

Effect(s) tested: N/A

Specify type of analysis: [ ] Whole brain [ ] ROI-based [x] Both

Anatomical location(s): The 360-region Glasser parcellation was used, which we coarse-grained into a set of 23 higher-order cortices.

Statistic type for inference (See Eklund et al. 2016): N/A

Correction: We performed FDR-BH corrections as well as Bonferroni corrections, as indicated in each instance.

Models & analysis

n/a	Involved in the study
[ ]	[x] Functional and/or effective connectivity
[ ]	[x] Graph analysis
[ ]	[x] Multivariate modeling or predictive analysis

Functional and/or effective connectivity: MIND networks were used to define brain connectivity for structural similarity networks (Sebenius et. al, 2023); Pearson correlation was used to define functional connectivity from fMRI data.

Graph analysis: We focused on edge-strength between each of the 23 cortical areas as our primary graph measure.

Multivariate modeling and predictive analysis: We identified genetic gradients of MIND network edges using gradient decomposition as implemented by the BrainSpace package (Vos de Wael et al, 2020), using the code parameters GradientMaps (kernel='normalized angle', approach='dm'), and fit without any thresholding.
