# Supplementary material for: The genetic architecture of cortical similarity networks
Source: Nat Commun. 2026 Jun 20;17:7801. doi: 10.1038/s41467-026-73714-9 (PMC13438111; doi:10.1038/s41467-026-73714-9)
Supplement: Supplementary file 3 [file 41467_2026_73714_MOESM3_ESM.pdf]

nature portfolio

Peer Review File

The genetic architecture of cortical similarity networks

Corresponding Author: Mr Isaac Sebenius

This file contains all reviewer reports in order by version, followed by all author rebuttals in order by version.

Version 0:

Reviewer comments:

Reviewer #1

(Remarks to the Author)
In this paper, Sebenius et al. reported their GWAS on a novel imaging phenotype, called Morphometric INverse Divergence (MIND), using UKBiobank imaging cohort. MIND is based on the correlations of structural features on cortical surface, claiming to capture more functionally relevant connectivities than other imaging phenotypes and a more stable connectivity measure than the resting state functional connectivities. The authors found several significant loci associated with MIND and performed series of post-GWAS inferences, including genetic correlations and Mendelian Randomization. They found that MIND can be summarized into a lower two-dimensional representation, G1 and G2, which coincidentally align with the purported trends of cortical differentiation. Based on those findings, the authors argued that their MIND imaging phenotype is a functionally relevant, genetically heritable, biologically informative, and clinically relevant feature. In general, I found this paper has done the due diligence as an imaging GWAS paper, extensively explore the relevant genetic signals from the imaging phenotype. However, this paper focused too much on the discussion about the validity of MIND and their relationship to resting state functional connectivity, while missing the comparisons with other imaging based GWAS. For instance, one loci they specifically mentioned, rs798511, has been reported to be GWAS significant with ventricle volumes. Yet the authors did not discuss why their findings provide more insight than other imaging based GWAS with the same UKBiobank data. Therefore, while I appreciate the efforts in devising a novel imaging phenotypes, I have the following comments/suggestions that hope to improve their paper accordingly.

Major:
1. Lack of comparisons with other structural imaging GWAS.
MIND is based on the structural features (T1 and diffusion), so it is inherently correlated with T1 and diffusion based metrics, such as cortical surface area, thickness, and diffusivity. Those metrics have been well studied in the context of GWAS, yet the actual comparisons are scarce. Maybe all the signals of G1 is about allometric scaling of human brain so that GWAS on total surface area is already good enough? How about GWAS results reported by Chen et al. on genetically derived parcellations of cortical surface area/thickness? They also found developmentally relevant gradients that are very similar to the two gradients reported here. Why MIND is more relevant than theirs? In this context, can the authors explain why the reported SNP heritability of MIND is smaller than other structural imaging features reported?

2. Functional relevancy of MIND.
The authors argued that MIND is a more useful metric than functional connectivity because it has higher heritability. This can be comparing apple to orange, as the functional connectivity is mapped onto the MIND regions and constrained by its signal to noise ratios (resting state in UKB is a short 7 minutes scan, notoriously noisy). Furthermore, the Mendelian Randomization seems suggest MIND G1 is irrelevant to psychiatric disorders, CRP, and BMI, despite its strong negative selection signal. G2, on the other hand, is reversely caused by BMI, which is also a known risk factor for other reported traits (MDD and CRP). This might mean the signals from the MIND were actually a down-stream epiphenomenon of certain disease process, such as obesity.

Minor:
1. The multiple comparison correction is not consistently applied across study. They have used GWAS significance level, experiment-wide significance level, FDR correction, and some form of Bonferroni correction with limited number of tests.

2. Why a strict two-sample MR is using IVW instead of pleiotropy robust approach? (line 273)

Reviewer #2

(Remarks to the Author)
This article by Sebenius et al. consists of a genetic analysis of a brain MRI phenotype called Morphometric INverse Divergence (MIND), which the authors introduced in a previous paper. The genetic analyses are standard (GWAS, LDSC, colocalization, MR) and methodologically sound. My main concern/question is about the impact and interest of these GWAS results, considering that the MIND phenotypes can be difficult to interpret and seem quite complex to generate, and likely sensitive to the input data (e.g., choice of metrics included). The MIND metrics integrate multimodal vertex-wise data from T1 and diffusion (cortical thickness, curvature, mean diffusivity, and neurite density), which is aggregated across regions of interest to build a 360x360 network, which is then reduced to a 23x23 network, which is then summarised by a 2D representation (factors G1 and G2). The interpretation of G1 and G2 as dimensions of paleocortical dual origin is interesting, but not really supported by the genetic analyses presented here. Even if true, it still feels like a simplistic representation of complex data, which will likely fail to capture the complexity of the brain. The authors identify genetic overlap between G1 and G2 and psychiatric dimensions (some of which might be explained by BMI? [minor]), but this is not necessarily groundbreaking or novel (rG/MR between brain structure and psychiatric domains has been reported before). I wonder if you would pick up the same signal using the first 2 PCs from a PCA performed on all brain features? In short, I was not convinced of the added value of studying MIND phenotypes, due to their complexity and lack of interpretability. Maybe a comparison of results against other approaches that can reduce the dimension (PCA, ICA, network-based approaches…) could help make a stronger case for the use of MIND in genetics.

Version 1:

Reviewer comments:

Reviewer #1

(Remarks to the Author)
The revised manuscript has addressed many of my concerns and showcased the unique aspects of the MIND they derived from multi-modal imaging. There are two remaining issues I hope the authors can improve upon:
1). Although the authors has used MR to test the potential causal relationships between their derived metrics and psychiatric disorders, it is by no means to be actually causal. This is especially true since the significance test results are marginal at best. I recommend to tone down the causal language and discuss the limitations.
2). It is good to see there are more comparisons to traditional uni-modal metrics to show MIND cannot be fully explained by one single modality or allometric scaling in the polygenic context. Yet it is still not addressing them in the loci level. This is in the same vein with the comparison to genetic map by Chen et al., whereas method differences cannot fully account for the uniqueness of the end results. Please put the discussion in context.

Reviewer #2

(Remarks to the Author)
Thank you for your revisions. I think some of them help make a better case about the MIND phenotypes and the findings.
I don't have more comments that would really improve the manuscript without wasting everyone's time in another round of review.

Open Access This Peer Review File is licensed under a Creative Commons Attribution 4.0 International License, which permits use, sharing, adaptation, distribution and reproduction in any medium or format, as long as you give appropriate credit to the original author(s) and the source, provide a link to the Creative Commons license, and indicate if changes were made.
In cases where reviewers are anonymous, credit should be given to 'Anonymous Referee' and the source.
The images or other third party material in this Peer Review File are included in the article's Creative Commons license, unless indicated otherwise in a credit line to the material. If material is not included in the article's Creative Commons license and your intended use is not permitted by statutory regulation or exceeds the permitted use, you will need to obtain permission directly from the copyright holder.
To view a copy of this license, visit https://creativecommons.org/licenses/by/4.0/

Dear Reviewers,

Thank you very much for your constructive comments on our manuscript, “The genetic architecture of cortical similarity networks.” Prompted by your suggestions, we have now included additional analyses, clarifications, and discussions into the work. In the following letter, we describe in detail how we have addressed each question raised. The remainder of the response is structured as follows:

• In black font, we restate each reviewer comment.

• In purple font, we offer a brief response and provide a description of the changes made to the manuscript to address the comment.

• In red font, we include the verbatim changes to the manuscript itself. Where applicable, **bold-faced** red font is used to highlight changes within an existing passage.

Given the overlapping themes emerging from different comments and reviewers, at times we repeat certain responses for ease of reading and to ensure each comment is addressed without the need for cross-referencing.

We sincerely hope that we have satisfactorily addressed the critical issues you have raised and, in doing so, we have been grateful for this opportunity to improve the interpretability and robustness of our results.

Yours sincerely,
Isaac Sebenius on behalf of the authors

Reviewer #1:

1.1: In this paper, Sebenius et al. reported their GWAS on a novel imaging phenotype, called Morphometric INverse Divergence (MIND), using UKBiobank imaging cohort. MIND is based on the correlations of structural features on cortical surface, claiming to capture more functionally relevant connectivities than other imaging phenotypes and a more stable connectivity measure than the resting state functional connectivities. The authors found several significant loci associated with MIND and performed series of post-GWAS inferences, including genetic correlations and Mendelian Randomization. They found that MIND can be summarized into a lower two-dimensional representation, G1 and G2, which coincidentally align with the purported trends of cortical differentiation. Based on those findings, the authors argued that their MIND imaging phenotype is a functionally relevant, genetically heritable, biologically informative, and clinically relevant feature. In general, I found this paper has done the due diligence as an imaging GWAS paper, extensively explore the relevant genetic signals from the imaging phenotype. However, this paper focused too much on the discussion about the validity of MIND and their relationship to resting state functional connectivity, while missing the comparisons with other imaging based GWAS. For instance, one loci they specifically mentioned, rs798511, has been reported to be GWAS significant with ventricle volumes. Yet the authors did not discuss why their findings provide more insight than other imaging based GWAS with the same UKBiobank data. Therefore, while I appreciate the efforts in devising a novel imaging phenotypes, I have the following comments/suggestions that hope to improve their paper accordingly.

We thank the reviewer for highlighting some of the strengths of the paper, especially the due diligence of our GWAS and subsequent functional genetic analyses of a novel MRI phenotype. We have addressed their principal caveat, also shared by Reviewer 2, concerning contextualisation of the genetic architecture of MIND in light of our own and previously published genetic studies of other MRI phenotypes, as set out in more detail below.

MIND is based on the structural features (T1 and diffusion), so it is inherently correlated with T1 and diffusion-based metrics, such as cortical surface area, thickness, and diffusivity. Those metrics have been well studied in the context of GWAS, yet the actual comparisons are scarce. Maybe all the signals of G1 is about allometric scaling of human brain so that GWAS on total surface area is already good enough?

We thank the reviewer for their suggestion. In this revision, we have included several novel analyses and additional text to better contextualize the genetics of MIND with those of other measures of brain structure. The analyses, and their resulting plots and discussion, are reflected in the manuscript as follows:

Lines 155-164:

We next studied the extent to which MIND gradients G1 and G2 were genetically related to other commonly-studied measures of whole-cortex structure. As shown in Extended Data Figure 4B, genetic correlations between G1/G2 and whole-brain CT, MD, ICVF, MC, and surface area (SA) (Warrier et al, 2023) were generally weak-moderate ($0.04 < |r_g| < 0.36$ for all comparisons),

although four of these comparisons were statistically significant (G1-SA $r_g = -0.12$; ICVF-G1 $r_g = -0.36$; G2-SA $r_g = -0.19$; G2-MD $r_g = 0.19$, all $P_{FDR} < 0.05$). Of note, the weak genetic correlations between global surface area and both G1 and G2 indicated that MIND gradients were not simply attributable to genetically determined allometric scaling of cortex. Indeed the generally low magnitude of these genetic correlations was not sufficient to explain the genetic architecture of multivariate MIND gradients in terms of the genetics of univariate MRI phenotypes.

Line 235-237:

Neither gradient was colocated with cortical maps of evolutionary expansion or areal allometric scaling, suggesting that the observed dual origin trends did not simply reflect changes in cortical size across species or individuals (Extended Data Figure 4D).

Extended Data Figure 4:

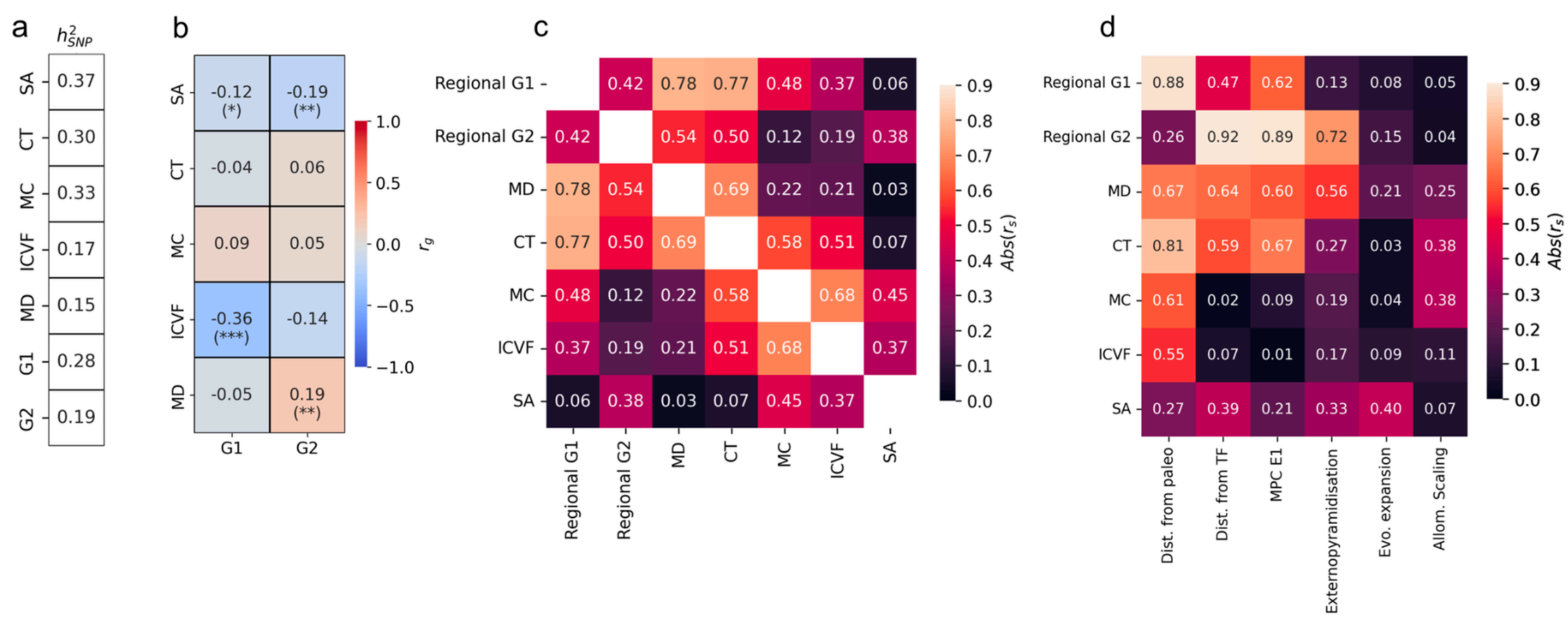

Extended Data Figure 4 **Genetic and spatial relationships between MIND gradients and univariate global measures of brain structure.** A) The SNP heritabilities of MIND G1/G2 and global measures of brain structure. MIND phenotype heritabilities were calculated using LDSC, and all other heritabilities were as reported in Warrier et al (2023). SA: surface area, ICVF: intracellular volume fraction, CT: cortical thickness, MC: mean curvature, MD: mean diffusivity. B) The genetic correlation (LDSC) between MIND G1/G2 and global measures of brain structure, using publicly-available summary statistics from Warrier et al (2023). P-values were FDR corrected: $^* < 0.01 < P < 0.05$, $^{**}0.001 < P < 0.01$, $^{***}P < 0.001$. C) The spatial relationships (absolute Spearman correlation) between MIND G1 and G2 regional scores and the first principal components of regional genetic similarity matrices SA, CT, MC, ICVF, and MD, from Warrier et al (2023). D) The spatial relationships (absolute Spearman correlation) of MIND G1 and G2 regional scores, and the principal genetic components from part (C), with six cortical maps indexing complementary aspects of dual origin theory, brain evolution, microstructure, and areal scaling. These include (1) the distance from paleocortex and (2) distance from area TF (the two dual-origin geodesic distance maps discussed in Fig. 3), (3) the principal gradient of the T1w/T2w microstructural profile covariance network (MPC E1), reflecting laminar myeloarchitectural complexity (Paquola et al, 2020), (4) cortical externopyramidisation, a measure of laminar cytoarchitectural differentiation (Amunts et al, 2013; Sydnor et al, 2021), (5) evolutionary expansion (Sydnor et al, 2021), and (6) allometric scaling (Sydnor et al, 2021).

We have also included additional specific discussion of rs79851, which the reviewer astutely noted was also previously implicated with normal pressure hydrocephaly and ventricular size (Räsänen et al., 2024).

Lines 410-417:

GNA12, encoding GCPR α-subunit 12, has been causally linked to several potentially relevant neurobiological processes including transmembrane signalling and regulating neuronal migration in the developing mouse cortex (Moers et al., 2008). As shown in Fig 2B, the locus of genetic association implicating *GNA12* also **includes a SNP (rs798511*)* that has been previously linked to normal pressure hydrocephalus and ventricular size (Räsänen et al., 2024). Building on these findings, we suggest that** this locus represents a specific and plausible set of genes pleiotropically associated with paleocortical trends in both structural similarity (G1) and directly-comparable functional connectivity, as well as trans-diagnostic risk of several neuropsychiatric disorders.

<u>1.2:</u> How about GWAS results reported by Chen et al. on genetically derived parcellations of cortical surface area/thickness? They also found developmentally relevant gradients that are very similar to the two gradients reported here. Why MIND is more relevant than theirs?

We agree that the work by Chen et al. (2013) was an important and relevant early contribution to the genetic study of brain structure. However, we respectfully wish to clarify multiple key points that differentiate our analyses from those of Chen et al. (2013).

Most importantly, Chen et al. (2013) reported a twin study, not a GWAS; comparable genomic analysis of their heritability results was thus precluded. Their paper, published more than 10 years ago, was methodologically leading at the time but nonetheless limited to using AE models on a sample of 203 twin pairs; whereas our analyses used contemporary imaging-genetic methods on a sample of >30,000 unrelated individuals.

Chen et al. (2013) studied regional CT and SA with the goal of finding cortical patterns of regional heritability (estimated by independent univariate AE), or inter-regionally shared heritability (using bivariate AE), that were only estimable on the basis of the entire sample, resulting in an average or canonical map of the genetic architecture of cortex that demonstrated 2 categorically distinct clusters (anatomically, dorso-ventral and antero-posterior).

In contrast, our approach combined multiple MRI features into one integrated measure of morphometric divergence with the goal of measuring inter-areal systems of cortical similarity and differentiation. We then used GWAS methods and data, rather than AE modeling of twin data, to investigate the genetic architecture of cortex-wide networks or gradients of inter-areal differentiation, down to the level of specific genes.

It is virtually impossible to make a direct comparison between Chen et al's results and our results, because of the major methodological differences between the studies, and because the Chen et al maps are not publicly available. However, we have added some discussion of the

Chen et al paper to highlight their early leadership of the general principle that genetic effects on human cortical structure are distributed and differentiated across multiple cortical areas, and to note the methodological points of difference between prior studies using twin data and contemporary imaging-genetic studies based on GWAS.

Lines 2-21:

Identifying the genetic basis of the human brain's complex network structure (Bullmore and Sporns, 2009; Bassett and Sporns, 2017) is essential for understanding how the brain develops, functions, and relates to health and cognition (Dennis et al, 2019). **Over a decade of work, rooted in seminal twin studies ,** has shown that individual differences in brain network organization measured by multiple modalities of magnetic resonance imaging (MRI) are under genetic influence (Schutte et al, 2013; Chiang et al, 2009; Glahn et al, 2010; Dennis et al, 2019). **Recent genome-wide association studies (GWAS) have since enabled the investigation of common genetic variation** associated with certain brain network phenotypes, including (f)MRI-derived functional connectivity (FC) from correlational analysis of regional time-series, and diffusion (d)MRI-derived structural connectivity (SC) from tractographic reconstruction of axonal projections between regions (Wainberg et al, 2024; Tissink et al, 2023; Roelfs et al, 2024; Foo et al, 2021; Cheng et al, 2024). However, our understanding of genetic influences on brain network organization remains fragmented. For example, it has proven difficult to identify any meaningful genetic overlap between brain structural and functional connectivity phenotypes derived from different MRI modalities (Tissink et al, 2023; Roelfs et al, 2024; Gajwani et al, 2023; Luppi et al, 2024). **The spatially-varying genetic architecture of localised (regional) MRI measures of brain structure has been well characterized by both twin studies (Chen et al, 2013; Wright et al., 2002) and more contemporary GWAS (Smith et al, 2021; Grasby et al, 2020; Zhao et al, 2019; Elliott et al, 2018; Warrier et al, 2023; Fu et al, 2024; Tissink et al, 2024)**. **It has also been shown by both twin studies (Chen et al, 2013) and GWAS (Warrier et al, 2023) that common genetic effects are largely distributed across major systems or classes of cortical areas, implying that formation of large-scale cortical gradients (of lamination) or cortical networks (of axonal or synaptic connectivity) are under genetic control.** However, it has not yet been shown how such genetically-driven cortical gradients derived from structural MRI data can be related to prior theories of cortical organisation, or measures of functional connectivity, or genetic risks for neuropsychiatric disorders and biomedical traits.

Nevertheless, we greatly appreciate the reviewer's point that it is important to contextualise the two principal gradients of genetically distributed effects on cortical similarity and differentiation (MIND G1 and G2) with prior genetic or phenotypic maps of structural cortical systems. To do so, we studied the spatial relationships between MIND G1 and G2 regional scores and the first two genetic principal components for each of the constituent MRI features: surface area (SA), cortical thickness (CT), mean curvature (MC), intracellular volume fraction (ICVF), and mean diffusivity (MD) (Warrier et al., 2023). We further compared these patterns of spatial genetic variation in brain structure with six cortical maps indexing complementary aspects of brain evolution, microstructural organization, and areal scaling. The results from these analysis are discussed and shown in Extended Data Figure 4C-D as follows:

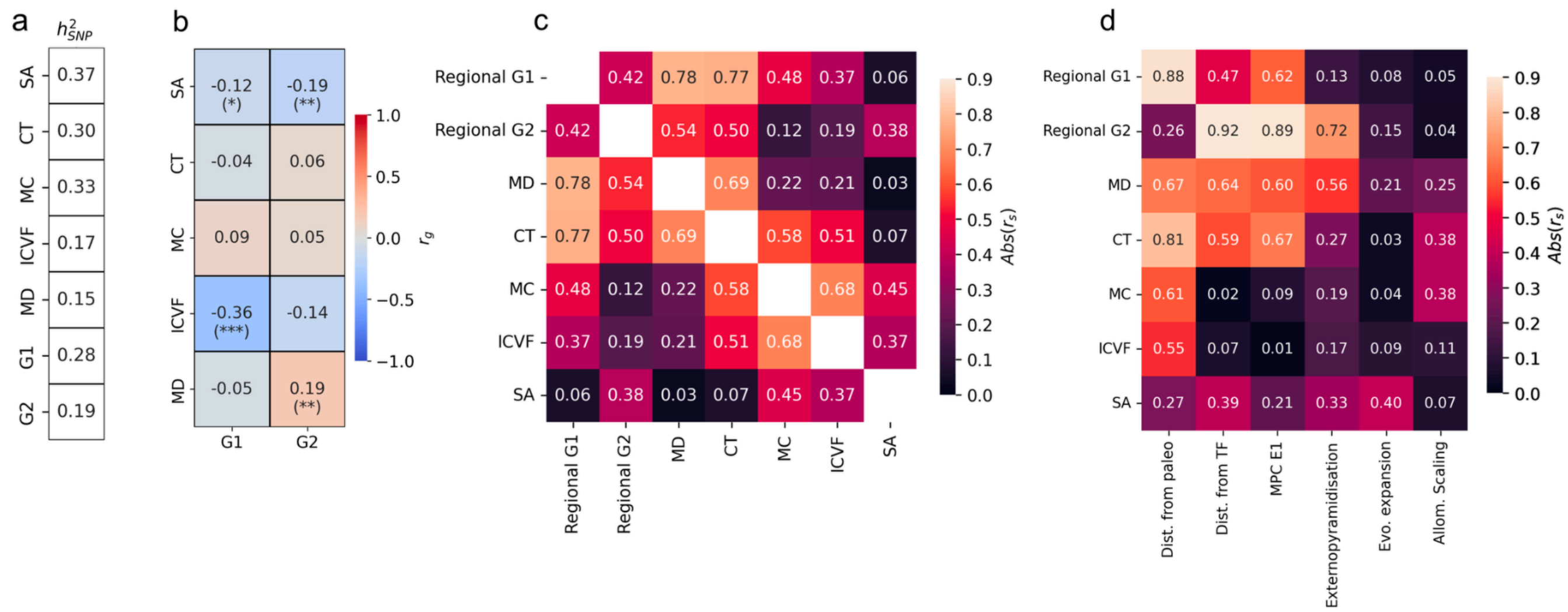

Extended Data Figure 4 **Genetic and spatial relationships between MIND gradients and univariate global measures of brain structure.** A) The SNP heritabilities of MIND G1/G2 and global measures of brain structure. MIND phenotype heritabilities were calculated using LDSC, and all other heritabilities were as reported in Warrier et al (2023). SA: surface area, ICVF: intracellular volume fraction, CT: cortical thickness, MC: mean curvature, MD: mean diffusivity. B) The genetic correlation (LDSC) between MIND G1/G2 and global measures of brain structure, using publicly-available summary statistics from Warrier et al (2023). P-values were FDR corrected: $^* < 0.01 < P < 0.05$, $^{**}0.001 < P < 0.01$, $^{***}P < 0.001$. C) The spatial relationships (absolute Spearman correlation) between MIND G1 and G2 regional scores and the first principal components of regional genetic similarity matrices SA, CT, MC, ICVF, and MD, from Warrier et al (2023). D) The spatial relationships (absolute Spearman correlation) of MIND G1 and G2 regional scores, and the principal genetic components from part (C), with six cortical maps indexing complementary aspects of dual origin theory, brain evolution, microstructure, and areal scaling. These include (1) the distance from paleocortex and (2) distance from area TF (the two dual-origin geodesic distance maps discussed in Fig. 3), (3) the principal gradient of the T1w/T2w microstructural profile covariance network (MPC E1), reflecting laminar myeloarchitectural complexity (Paquola et al, 2020), (4) cortical externopyramidisation, a measure of laminar cytoarchitectural differentiation (Amunts et al, 2013; Sydnor et al, 2021), (5) evolutionary expansion (Sydnor et al, 2021), and (6) allometric scaling (Sydnor et al, 2021).

Lines 238-251:

Finally, we compared the spatial patterns of MIND G1 and G2 with the first genetic principal components of each of several structural MRI features (Warrier et al., 2023). Areal G1 and G2 scores showed varying degrees of spatial alignment with the genetic PCs of individual MRI features ($0.06 < |r_s| < 0.77$; Extended Data Figure 4C). Interestingly, strong spatial alignments (e.g. areal G1–CT PC1 $r_s = 0.77$) did not reflect strong underlying genetic correlations (G1–global CT $r_g = -0.04$; Extended Data Figure 4B), suggesting that similar spatial patterning did not necessarily imply genetic concordance. Paleocortical distance was more strongly associated with areal G1 than with the first genetic principal component of any individual structural feature (Extended Data Figure 4D). Similarly, distance from parahippocampal area TF was more strongly correlated with areal G2 than with any of the univariate genetic principal components. Additionally, areal G2 showed stronger correlations with brain maps indexing laminar complexity of cortical myeloarchitecture (i.e. the first microstructural profile covariance gradient; $|r_s| = 0.89$) and cytoarchitecture (i.e. externopyramidization; $|r_s| = 0.72$) than did individual feature PCs (Extended Data Figure 4D). Overall, these results indicated that the integrative, multivariate MIND gradients G1 and G2 were more precisely aligned with the

anatomical predictions of dual origin theory than the genetic principal components of any of the individual, univariate structural MRI features that were used to estimate MIND.

1.3: The authors argued that MIND is a more useful metric than functional connectivity because it has higher heritability. This can be comparing apple to orange, as the functional connectivity is mapped onto the MIND regions and constrained by its signal to noise ratios (resting state in UKB is a short 7 minutes scan, notoriously noisy).

This is a complex and interesting point to which our response is broken down into three components as follows:

The authors argued that MIND is a more useful metric than functional connectivity because it has higher heritability.

We do not argue that increased heritability makes MIND more useful than functional connectivity. Rather, we suggest that MIND serves as a novel and useful bridge between genetics, structure, and function – MIND being substantially more heritable than FC while also showing robust and concordant genetic correlations with FC. This positions MIND as a novel complement to both univariate structural MRI phenotypes and (relational) functional connectivity measures. We have now included the following text to clarify and emphasize these points as follows:

Lines 333-339:

Taken together, **these results position MIND as a novel and useful bridge between genetics, univariate brain structure, and functional connectivity: MIND being a structurally-derived relational measure that is substantially more heritable than the functionally-derived relational measure of FC, while also being robustly and concordantly correlated with FC genetically.**

Lines 32-34:

As such, structural similarity is positioned to bridge structural MRI features defined locally (e.g. cortical thickness) with network-based measures that are defined relationally between brain areas (e.g. functional connectivity).

To offer further support of this claim, we confirm that the strong genetic relationships observed between MIND and FC could not be explained by genetic correlations with univariate measures of global brain structure. The results and discussion of this confirmatory analysis are shown in Extended Data Figure 6B and discussed as follows:

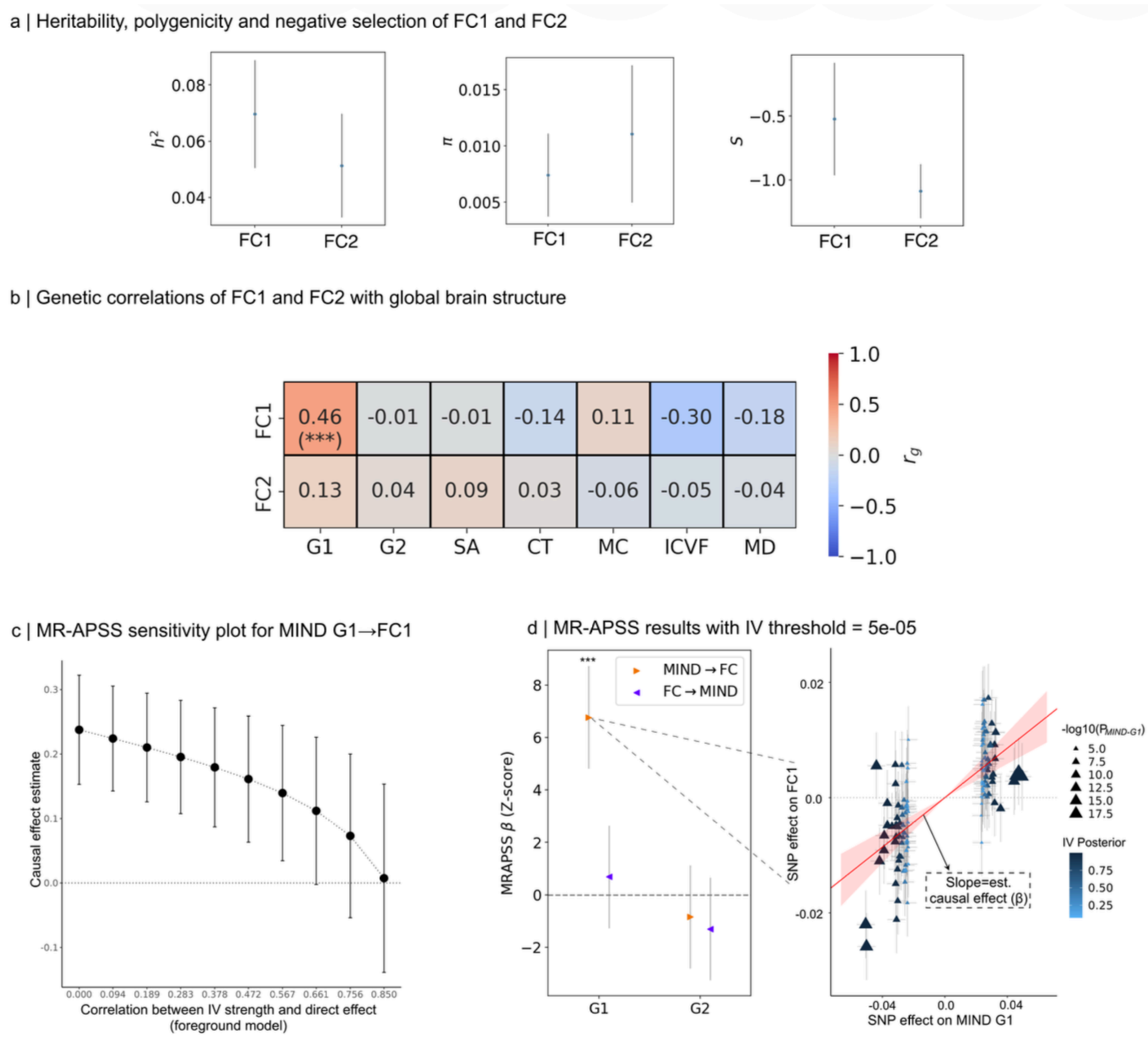

Extended Data Figure 6 Genetic relationships between FC, global brain structure, MIND gradients. A) Estimates of SNP-based heritability, polygenicity parameter π, and negative selection parameter S of fMRI-derived functional connectivity (FC) summarised along the two principal MIND genetic gradients (labeled FC1 and FC2). All estimates were calculated using SBayesS (Zeng et al, 2018). Shaded lines indicated 95% confidence intervals. B) The genetic correlation (LDSC) between FC1 and FC2 with MIND G1, MIND G2, and five univariate global measures of brain structure, using publicly-available summary statistics from Warrier et al (2023). SA: surface area, ICVF: intracellular volume fraction, CT: cortical thickness, MC: mean curvature, MD: mean diffusivity. P-values were FDR corrected: $* < 0.01 < P < 0.05$, $** 0.001 < P < 0.01$, $*** P < 0.001$. C) Sensitivity plot for the result shown in Figure 4E showing a significant causal estimate of MIND G1 on FC1. This plot tests the robustness of the result to model assumptions about the relationship between instrumental variable (IV) strength and the direct causal effect in the foreground model, which is assumed to be zero (Hu et al, 2022). The plot shows here that the positive causal effect of MIND on FC along G1 result remains significant as long as this value remains below 0.661. To put this number in context, the result remains significant for over twice as large a parameter space as the causal evidence for body mass index (BMI) on Type II diabetes, a widely-accepted causal relationship which was previously shown to be robust for values below 0.247 (Hu et al, 2022). D) A replication of the gradient-level MR-APSS results shown in Figure 4E, but with an IV threshold of $5e-05$ instead of $5e-04$. The left plot shows estimated (forward and reverse) causal relationships between MIND and FC (with 95% CI) separately for both G1 and G2. The right plot shows the highly significant causal effect of G1 on FC1, which is shown by the significantly positive slope of the regression plot (Z-scored β=6.2, $P = 6.0$e-10).

Lines 306-317:

The genetic relationship between MIND and FC was clarified and strengthened through gradient-level comparison. On the paleocortical trend represented by G1, MIND G1 scores and FC1 scores were significantly genetically correlated at approximately the same level as the most strongly genetically correlated edges (HDL r_g = 0.44, P = 1.2e-04; LDSC r_g = 0.46, P = 6.5e-08). **FC1 was not significantly genetically correlated with univariate global measures of brain structure (CT, MC, ICVF, MD, or SA; Extended Data Fig. 6B), suggesting that the genetic overlap between MIND G1 and FC1 reflected a shared network organization rather than associations with simpler univariate structural metrics.** However, on the archicortical trend represented by G2, there was no significant genetic correlation between MIND G2 and FC2 scores (HDL r_g = 0.04, P = 0.61; LDSC r_g = 0.05, P = 0.76; Fig. 4D). This result suggests that the two genetically independent trends of cortical architecture, defined by structural MRI network analysis and aligned with dual origin theory based on cortical histology, have quite distinct relationships to brain functional activity.

[Comparing heritability of MIND and fMRI phenotypes] is constrained by the signal to noise ratios (resting state in UKB is a short 7 minutes scan, notoriously noisy).

While UKB resting-state fMRI are 7 minutes long, this duration is by no means unusual for large-scale studies, and several studies have successfully performed GWAS on UKB rs-fMRI data already (e.g. Elliott et al., 2018). Additionally, even if rs-fMRI scans were available with longer acquisition time and higher signal-to-noise ratio, this would likely increase heritability and thus *increase* the significance of MIND-FC genetic relationships. As such, we note our current results should represent the *lower bound* on the true genetic relationship between MIND and FC. We include this point to the text as follows:

Lines 333-339:

Taken together, **these results position MIND as a novel and useful bridge between genetics, univariate brain structure, and functional connectivity: MIND being a structurally-derived relational measure that is substantially more heritable than the functionally-derived relational measure of FC, while also being robustly and concordantly correlated with FC genetically. Given the relatively low heritability of the FC phenotypes studied, and the short duration and/or low signal-to-noise of fMRI time series data available at sufficient scale to support GWAS, the observed genetic overlaps between MIND and FC likely represent the lower bound of their true genetic relationship.**

[Comparing heritability of MIND and fMRI phenotypes] can be comparing apple to orange, as the functional connectivity is mapped onto the MIND region.

We demonstrated robust and concordant MIND-FC genetic relationships at the edge level where both phenotypes are defined identically (same 276 edges, same parcellation). This comparison was conducted on equal footing in a strict like-for-like manner, eliminating concerns of biased comparisons. We include the following text to clarify this methodology as follows:

Line 274-281:

To do this, we conducted an additional set of 276 GWAS on the same set of 276 edges as defined for the MIND networks, and in a subsample (N_{max}=30,376) of the same UK Biobank participants; but now with each edge representing the correlation between regional mean fMRI time series recorded with the participant in a resting state (Fig. 4A). **These edge-level GWAS for the relational measure of functional connectivity were thus estimated in the same individuals, using an identical cortical parcellation, as previously used for edge-level GWAS of MIND, a relational measure of cortical similarity. This methodological alignment between MIND and FC allowed us to directly compare the genetics of cortical similarity and functional connectivity on a like-for-like, unbiased basis.**

<u>1.4:</u> Can the authors explain why the reported SNP heritability of MIND is smaller than other structural imaging features reported?

We apologise for any lack of clarity in our presentation of these data. The SNP heritability of MIND is not smaller than all other structural imaging features studied. For example, the heritabilities of MIND G1 and G2 are 0.28 and 0.19, respectfully. The SNP heritabilities of global MD, ICVF, MC, and CT (the four constituent structural features), are 0.15, 0.17, 0.33, 0.3, respectively (as reported in Warrier et al. (2023), which curated and analyzed the primary sample used in our present study). Thus, as expected, the two MIND gradients demonstrate heritabilities that are intermediate compared to those of the structural features from which they are constructed – not smaller.

We present these results more clearly in the text as follows:

Extended Data Figure 4A:

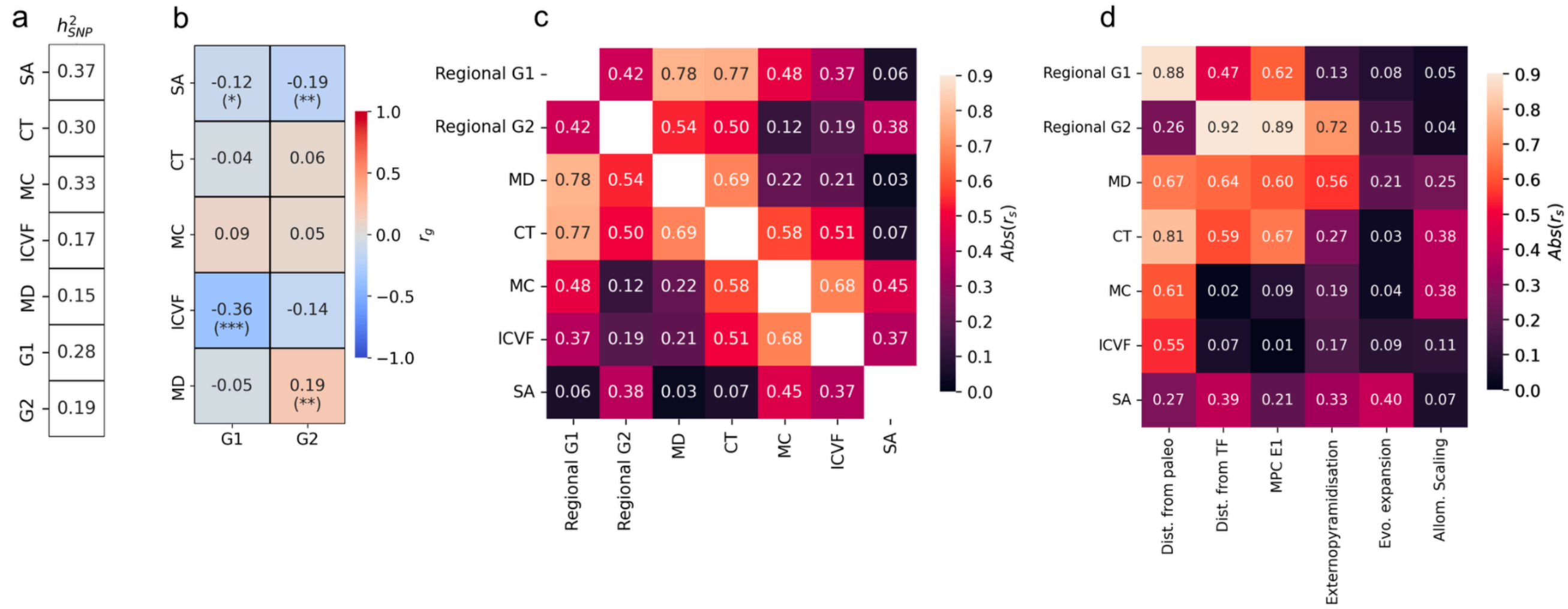

Extended Data Figure 4 **Genetic and spatial relationships between MIND gradients and univariate global measures of brain structure.** A) The SNP heritabilities of MIND G1/G2 and global measures of brain structure. MIND phenotype heritabilities were calculated using LDSC, and all other heritabilities were as reported in Warrier et al (2023). SA: surface area, ICVF: intracellular volume fraction, CT: cortical thickness, MC: mean curvature, MD: mean diffusivity. B) The genetic correlation (LDSC) between MIND G1/G2 and global measures of brain structure, using publicly-available summary statistics from Warrier et al (2023). P-values were FDR corrected: $* < 0.01 < P < 0.05$, $**0.001 < P < 0.01$, $***P < 0.001$. C) The spatial relationships (absolute Spearman correlation) between MIND G1 and G2 regional scores and the first principal components of regional genetic similarity matrices SA, CT, MC, ICVF, and MD, from Warrier et al (2023). D) The spatial relationships (absolute Spearman correlation) of MIND G1 and G2 regional scores, and the principal genetic components from part (C), with six cortical maps indexing complementary aspects of dual origin theory, brain evolution, microstructure, and areal scaling. These include (1) the distance from paleocortex and (2) distance from area TF (the two dual-origin geodesic distance maps discussed in Fig. 3), (3) the principal gradient of the T1w/T2w microstructural profile covariance network (MPC E1), reflecting laminar myeloarchitectural complexity (Paquola et al, 2020), (4) cortical externopyramidisation, a measure of laminar cytoarchitectural differentiation (Amunts et al, 2013; Sydnor et al, 2021), (5) evolutionary expansion (Sydnor et al, 2021), and (6) allometric scaling (Sydnor et al, 2021).

Lines 139-141:
Both MIND gradients were more heritable than MIND edges (on average): h^2 G1 = 0.281 ± 0.035; h^2 G2 = 0.192 ± 0.022 (Extended Data Figures 3C **and 4A)**.

Line 526:
The h^2_{SNP} of the final four selected features are reported in **Extended Data Figure 4A**.

1.5: Furthermore, the Mendelian Randomization seems suggest MIND G1 is irrelevant to psychiatric disorders, CRP, and BMI, despite its strong negative selection signal. G2, on the other hand, is reversely caused by BMI, which is also a known risk factor for other reported traits (MDD and CRP). This might mean the signals from the MIND were actually a down-stream epiphenomenon of certain disease process, such as obesity.

We respectfully disagree with the Reviewer's comment that MIND G1 is irrelevant to psychiatric disorders. We apologise for any lack of clarity in our presentation of the relevant results, which we have revised as follows:

Lines 391-400:

We observed a higher number of significant local genetic correlations as compared to global genetic correlations (86% of MIND-trait pairs had ≥1 significant local r_g versus 32% with significant global r_g), with many local correlations in discordant directions (Fig. 5A). Although MIND gradient G1 had fewer significant global genetic correlations with diagnostic and biomedical traits than G2, it had more significant local genetic correlations **(indeed, MIND G1 was significantly locally correlated with every studied phenotype)**. For example, by global genetic correlation analysis CRP, ADHD, and MDD were only significantly linked to G2, not G1; but all these traits had more significant local genetic correlations with G1 than with G2. **Local genetic correlations specifically linked G1 (and not G2) to Alzheimer's disease (four loci), bipolar disorder (three loci), and gestational age at birth (three loci) – associations that were not apparent at the global level (Fig. 5A).**

Lines 411-414:
Building on these findings, we suggest that this locus [within 7p22.3] represents a specific and plausible set of genes pleiotropically associated with paleocortical trends in structural similarity **(G1)** and directly-comparable functional connectivity, as well as trans-diagnostic risk of several neuropsychiatric disorders.

Lines 367-371:
The paleocortical trend of structural similarity represented by G1 was globally genetically correlated with schizophrenia (SCZ; r_g=0.08, P=0.03 corrected for two gradient-level tests). **Follow up analysis suggested a forward (but not reverse) causal relationship between G1 and schizophrenia (G1 → SCZ, MR-APSS β = 0.037, P = 0.04 uncorrected; SCZ → G1, MR-APSS β=-0.004, P=0.90 uncorrected; Extended Data Table 1), indicating that paleocortical architecture may causally contribute to schizophrenia risk.**

We agree with Reviewer's comment that the reverse causal relationships observed for G2 (BMI → G2, CRP → G2) suggest that this gradient may partially reflect downstream consequences of metabolic or immune processes. This is an important finding indicating that immunometabolic health may influence cortical architecture along archicortical trends. We have adapted the text to clarify these results and avoid overstating causality:

Line 378-385:

Subsequent MR analysis demonstrated no forward causal effect of G2 on any disorders or traits (Extended Data Table 1). However, there was strong evidence for a reverse causal effect of BMI on G2 (MR-APSS β=-0.15, P = 8.0e-04 uncorrected) and suggestive evidence for a reverse causal effect of CRP on G2 (MR-APSS β=-0.07, P = 0.045 uncorrected), **indicating that immuno-metabolic health may have downstream effects on archicortically aligned trends of cortical architecture**. These results provide some preliminary support for the prior prediction arising from dual origin theory (Pandya et al., 2015) that the two trends of cortical architectonic differentiation may be differentially implicated in pathogenesis of neuropsychiatric disorders.

1.6: The multiple comparison correction is not consistently applied across study. They have used GWAS significance level, experiment-wide significance level, FDR correction, and some form of Bonferroni correction with limited number of tests.

We thank the reviewer for pointing this out. As we are conducting a wide range of different analyses, we endeavoured to use the most appropriate correction for each analysis. To make this clearer in the paper, we have added a new section in Methods, "Statistical hypothesis testing," where we clarify and justify the use of correction levels across our different analyses.

Lines 644-652:

For GWAS-level tests, we used $P < 5\times10^{-8}$ (standard genome-wide significance). For edge-level tests we defined experiment-wide significance at $P < 1.8\times10^{-10}$ (5×10^{-8}/276 edges). For gradient-level tests, we defined experiment-wide significance at $P < 2.5\times10^{-8}$ (5×10^{-8}/2 gradients).These Bonferroni corrections follow standard practice for neuroimaging-genetics studies (Warrier et al., 2023). All other tests used false discovery rate (FDR) correction at 5%, unless otherwise noted, and were corrected for the number of tests specific to each analysis. These FDR tests are appropriate for correlated tests where Bonferroni would be overly conservative. P-values from follow-up Mendelian Randomization analyses were not corrected for multiple comparisons (Extended Data Table 1).

Additionally, we agree that for consistency, our gradient-level GWAS should include a correction for two gradient-level tests (to mirror the correction for number of edges considered in edge-level analyses), as the reviewer notes. We have correspondingly amended the results and text as follows. We note that these improvements to the consistency of multiple comparisons correction across studies do not substantially change the pattern of significant results.

Lines 142-146:

G1 was significantly associated with 31 loci at the genome-wide threshold of $P < 5\times10^{-8}$; G2 was associated with a single locus at this threshold (see Extended Data Fig. 3A). **After additionally correcting for two gradient-level tests, G1 and G2 were significantly associated with 26 and 0 loci, respectively.** This discrepancy between the number of associated loci for G1 and G2 was consistent with our finding that G1 was roughly ten times *less* polygenic than G2 (π_{G1}=0.33%, π_{G2}=3.7% using SBayesS; Extended Data Fig. 3C) (Zeng et al, 2018, 2021).

1.7: Why a strict two-sample MR is using IVW instead of pleiotropy robust approach? (line 273)

For this replication analysis, we wanted to confirm that the MIND→FC relationship would hold when analyzed using the most widely-used two-sample MR, which is IVW. However, we additionally replicate the result using pleiotropy-robust approaches such as weighted-median (Extended Data Figure 7), with no meaningful change in the results. We highlight these results and points in the main text.

Line 328-332:

The causal relationship of MIND G1 on FC1 was significantly replicated when using MIND summary statistics from the (non-overlapping) replication cohort in a strict two-sample setting (IVW $\beta=0.19$, $P=1.3e-05$; **Weighted Median $\beta = 0.14$, $P=1.7e-03$**; Extended Data Fig. 5B), and was robust across a **range of commonly-used two-sample MR methods** and sensitivity analyses (Extended Data Figs. 6C-D, 7).

Reviewer #2:

2.1: This article by Sebenius et al. consists of a genetic analysis of a brain MRI phenotype called Morphometric INverse Divergence (MIND), which the authors introduced in a previous paper. The genetic analyses are standard (GWAS, LDSC, colocalization, MR) and methodologically sound.

We thank the reviewer for this evaluation of the methodological soundness of the study.

2.2: My main concern/question is about the impact and interest of these GWAS results, considering that the MIND phenotypes can be difficult to interpret and seem quite complex to generate, and likely sensitive to the input data (e.g., choice of metrics included).

This is an important and complex point and we will break down our response in two components for clarity.

MIND phenotypes can be difficult to interpret and seem quite complex to generate

We have extensively revised and expanded the introduction to more clearly articulate why the current study is both well-justified and of urgent interest and impact. In this new discussion, we highlight two key points. First, structural similarity, and MIND networks in particular, have a rich theoretical and empirical basis that positions them to bridge structural MRI features defined locally (e.g. cortical thickness) with network-based measures that are defined relationally between brain areas (e.g. functional connectivity). Second, and more practically, since its recent development, the method has already been cited over 100 times by studies applying it to a wide range of neuroscientific applications, including lifespan brain development (Liang et al, 2025), neuropsychiatric and neurodegenerative disorders (Estevez-Fraga et al, 2025; García-San-Martín et al, 2025; Zou et al, 2025; Montagnese et al, 2025; Ge et al, 2025), cross-species connectomics (Hutchings et al, 2025; Smith et al, 2025), infectious disease (Li et al., 2025), empathy (Yu et al., 2025), cognition (Han et al., 2025), and beyond. Within this context of broad and increasing use, a comprehensive genetic analysis of MIND network phenotypes is not only theoretically-justified, but also represents a timely contribution to the research community to inform existing and future studies using the method. We reflect these important points in a substantially revised introduction as follows:

Lines 26-49:

To address these key knowledge gaps, we used an innovative approach to human MRI network phenotyping, based on estimating the structural similarity between cortical areas. It has already been established that the similarity between cortical areas which is measurable at the whole-brain scale of MRI, e.g., in terms of inter-areal correlation of macro- and/or micro-structural MRI feature vectors, is representative of the architectonic similarity between areas at a cellular scale (Seidlitz et al, 2018; Paquola et al, 2019; Sebenius et al, 2023, 2024). Architectonically similar areas of cortex are also more likely to be inter-connected by axonal projections (Beul et al, 2015, 2017; Wei et al, 2018). As such, structural similarity is well positioned to bridge structural MRI

features defined locally (e.g. cortical thickness) with network-based measures that are defined relationally between brain areas (e.g. functional connectivity).

Several methods have emerged recently to estimate the connectome from MRI similarity analysis (Wang and He, 2024; Paquola et al, 2019; Snyder et al, 2024; Sebenius et al, 2023). We used Morphometric INverse Divergence (MIND) for several technical, biological, and practical reasons. First, it has been biologically validated and technically benchmarked favourably against alternative structural connectivity and similarity estimators (Sebenius et al, 2023). Importantly, MIND similarity was strongly co-located with cortical patterns of genomic co-expression, and has moderate twin-based heritability (average nodal $h^2_{SNP} \sim 0.15$), indicating that MIND phenotypes might be advantageous for analysis of genetic effects on brain networks (Sebenius et al, 2023). Finally, since its recent development, MIND has been rapidly taken up by the research community for the analysis of brain network organization across many different applications, including lifespan development (Liang et al, 2025), neuropsychiatric and neurodegenerative disorders (Estevez-Fraga et al, 2025; García-San-Martín et al, 2025; Zou et al, 2025; Montagnese et al, 2025; Ge et al, 2025), cross-species connectomics (Hutchings et al, 2025; Smith et al, 2025), and beyond. As such, a comprehensive genetic analysis of MIND network phenotypes is not only empirically and theoretically justified, but also timely.

MIND phenotypes are likely sensitive to the input data (e.g., choice of metrics included).

In previous work, we showed that a) multivariate MIND network structure is not dominated by a single feature, and that b) including a larger number of complementary features increases the alignment between structural similarity and biological benchmarks such as axonal connectivity and gene expression similarity (Sebenius et al., 2023). Building on this, in the present study we aimed to create an integrated measure of structural similarity by carefully selecting features based on three principled criteria: (1) vertex-level interpretability, (2) genetic complementarity, and (3) heritability. We have adapted the following text to highlight these points.

Lines 88-91:

MIND estimates similarity between cortical areas by comparing their vertex- or voxel-level distributions of one or more structural MRI features. **Previous work has shown that multivariate MIND network structure is not dominated by a single feature, and that including a larger number of complementary features increases the alignment between structural similarity and biological benchmarks such as axonal connectivity and gene expression similarity (Sebenius et al, 2023).** On this basis, we aimed to use an integrative measure of structural similarity, representative of multiple, complementary aspects of cortical organization. From the range of MRI features potentially available in the UKB MRI cohort, we therefore selected four MRI features that were **interpretable, heritable, and complementary** (see Methods for details): two macro-structural metrics - cortical thickness (CT) and mean curvature (MC); and two micro-structural metrics - mean diffusivity (MD) and neurite density index (NDI).

We have additionally revised the section “Structural MRI feature selection” for a more extensive discussion of the feature selection and justification process used in our study as follows:

Lines 482-526:

Structural MRI feature selection

Structural similarity necessarily depends on the measurements of brain structure on which it is based. **We previously showed that multivariate networks are both more stable and more aligned with benchmarks of biological validity; and that multivariate MIND network architecture is not disproportionately influenced by a single feature (Sebenius et al., 2023) . On this basis, we estimated similarity based on a diverse set of interpretable macro- and micro-structural MRI features, and aimed to select these features in a principled manner**. We considered a total possible set of 13 structural MRI phenotypes which have previously been genetically analyzed in a recent GWAS study (Warrier et al, 2023): these included expansion-related phenotypes (e.g., surface area; SA, volume; Vol), cortical thickness (CT), curvature phenotypes (mean curvature; MC, Gaussian curvature; GC), standard diffusion-based phenotypes (mean diffusivity; MD, fractional anisotropy; FA) and neurite orientation dispersion and density imaging (NODDI) features from diffusion-weighted imaging (orientation dispersion index; ODI, isotropic volume fraction; ISOVF, and neurite density index; NDI, also known as intracellular-volume fraction; ICVF). From among these 13 phenotypes, we selected a final set of four MRI features as the basis for MIND similarity network estimation, each of which satisfied the following three criteria:

- *Local (vertex-level) interpretability*. MIND calculates structural similarity by comparing regional distributions of structural features measured at the level of individual vertices on the cortical surface. Therefore, we only considered features that were well-defined at the level of the individual vertex. For this reason, we excluded all expansion-based phenotypes including surface area and volume, which are only well-defined by aggregating (summing) information across vertices. Measured at the vertex level, these measurements are a direct function of the size and number of vertices used to reconstruct the surface mesh. Cortical thickness, curvature-based phenotypes, and diffusion-based phenotypes passed this criterion.

- *Genetic complementarity.* We aimed to integrate features capturing diverse genetic signals while avoiding the inclusion of multiple features that capture redundant signal. Warrier et al (2023) calculated the genetic correlations between each of the 13 globally-measured phenotypes and identified five genetically-defined clusters of structural features, representing: 1) expansion, 2) curvature, 3) thickness, 4) neurite density and orientation, and 5) water diffusion. To minimize redundancy while maximizing signal diversity, we included one feature from each cluster (except the expansion-related cluster excluded by the interpretability criterion).

- *SNP-based heritability*. CT was the only representative of the thickness cluster and had high heritability ($h^2 = 0.3$). For each of the other three clusters, we selected a representative feature primarily by maximizing SNP-based heritability. For this reason, from the cluster of curvature phenotypes (MC and GC) we selected MC (h^2=0.33 vs h^2=0.19 for GC), and from the cluster of neurite density and orientation phenotypes (NDI,

> FA, and ODI), we selected NDI (h^2=0.17 vs h^2=0.11 for FA and h^2=0.07 for ODI). The only exception to this preference for the highest heritability feature was for the water diffusion cluster (MD and ISOVF), where we selected MD (h^2=0.15) over ISOVF (h^2=0.24) as MD is more widely used in the neuroimaging community.

We settled on two macro-structural MRI phenotypes (mean curvature, MC; cortical thickness, CT) and two micro-structural phenotypes (mean diffusivity, MD; neurite density index, NDI). MC and CT were automatically defined at the vertex-level via default FreeSurfer outputs. MD and NDI were mapped from volumetric space (voxel resolution) to the vertex-level resolution of the cortical mesh via a surface- based registration in line with Human Connectome Project protocols, as described in Warrier et al (2023). **The h^2_{SNP} of the final four selected features are reported in Extended Data Figure 4A.**

2.3: The MIND metrics integrate multimodal vertex-wise data from T1 and diffusion (cortical thickness, curvature, mean diffusivity, and neurite density), which is aggregated across regions of interest to build a 360×360 network, which is then reduced to a 23×23 network, which is then summarised by a 2D representation (factors G1 and G2).

We agree with this summary of our methodology.

2.4: The interpretation of G1 and G2 as dimensions of paleocortical dual origin is interesting, but not really supported by the genetic analyses presented here. Even if true, it still feels like a simplistic representation of complex data, which will likely fail to capture the complexity of the brain.

We break down our response to this important comment in two components for clarity.

The interpretation of G1 and G2 as dimensions of paleocortical dual origin is interesting, but not really supported by the genetic analyses presented here.

We include several new analyses to provide further support of a dual-origin interpretation of our results. We tested the hypotheses that, should this interpretation be supported, (1) dual origin axes should be more closely aligned with G1 and G2 than with previously-published genetic principal components of constituent univariate structural features; and that (2) G1 and G2 should be strongly correlated with other brain maps measuring variation in laminar cytoarchitectural and myeloarchitectural complexity. The results of these analyses are summarized in Extended Data Figure 4 and discussed in the text as follows:

Extended Data Figure 4:

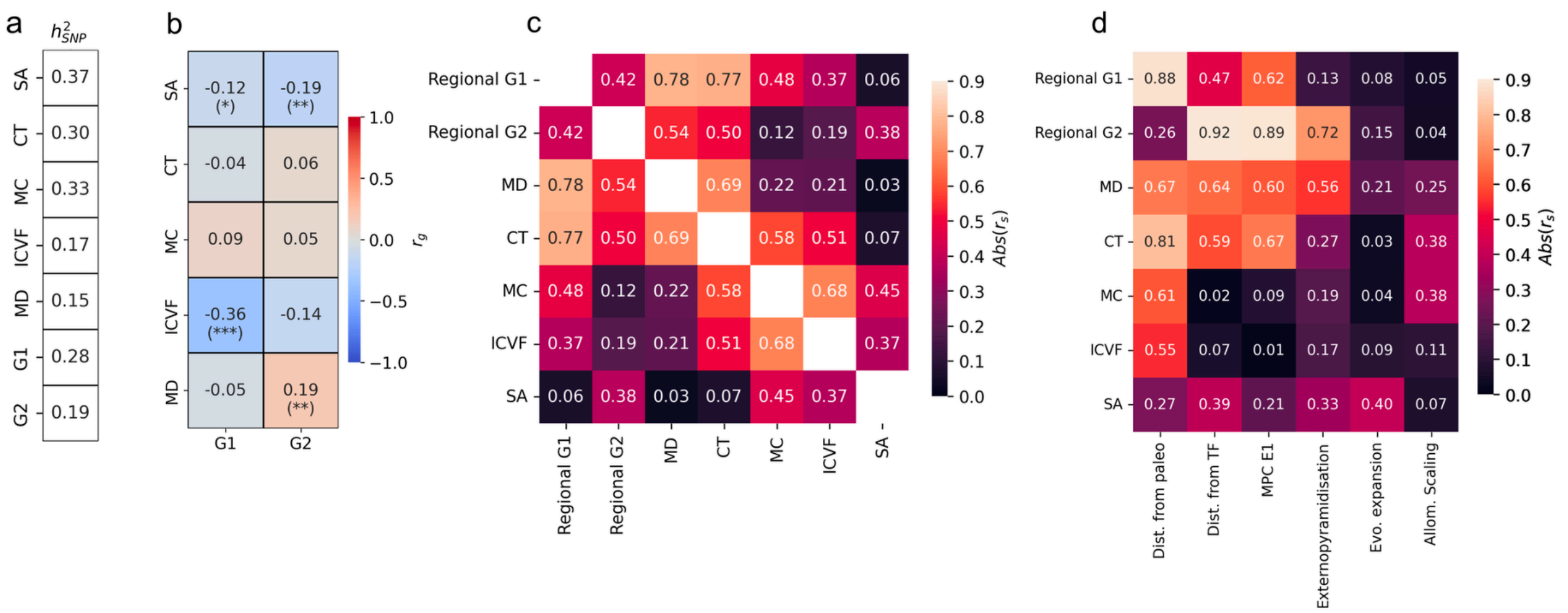

Extended Data Figure 4 **Genetic and spatial relationships between MIND gradients and univariate global measures of brain structure.** A) The SNP heritabilities of MIND G1/G2 and global measures of brain structure. MIND phenotype heritabilities were calculated using LDSC, and all other heritabilities were as reported in Warrier et al (2023). SA: surface area, ICVF: intracellular volume fraction, CT: cortical thickness, MC: mean curvature, MD: mean diffusivity. B) The genetic correlation (LDSC) between MIND G1/G2 and global measures of brain structure, using publicly-available summary statistics from Warrier et al (2023). P-values were FDR corrected: $* < 0.01 < P < 0.05$, $**0.001 < P < 0.01$, $***P < 0.001$. C) The spatial relationships (absolute Spearman correlation) between MIND G1 and G2 regional scores and the first principal components of regional genetic similarity matrices SA, CT, MC, ICVF, and MD, from Warrier et al (2023). D) The spatial relationships (absolute Spearman correlation) of MIND G1 and G2 regional scores, and the principal genetic components from part (C), with six cortical maps indexing complementary aspects of dual origin theory, brain evolution, microstructure, and areal scaling. These include (1) the distance from paleocortex and (2) distance from area TF (the two dual-origin geodesic distance maps discussed in Fig. 3), (3) the principal gradient of the T1w/T2w microstructural profile covariance network (MPC E1), reflecting laminar myeloarchitectural complexity (Paquola et al, 2020), (4) cortical externopyramidisation, a measure of laminar cytoarchitectural differentiation (Amunts et al, 2013; Sydnor et al, 2021), (5) evolutionary expansion (Sydnor et al, 2021), and (6) allometric scaling (Sydnor et al, 2021).

Lines 238-251:

Finally, we compared the spatial patterns of MIND G1 and G2 with the first genetic principal components of each of several structural MRI features (Warrier et al., 2023). Areal G1 and G2 scores showed varying degrees of spatial alignment with the genetic PCs of individual MRI features ($0.06 < |r_s| < 0.77$; Extended Data Figure 4C). Interestingly, strong spatial alignments (e.g. areal G1–CT PC1 $r_s = 0.77$) did not reflect strong underlying genetic correlations (G1–global CT r_g = -0.04; Extended Data Figure 4B), suggesting that similar spatial patterning did not necessarily imply genetic concordance. Paleocortical distance was more strongly associated with areal G1 than with the first genetic principal component of any individual structural feature (Extended Data Figure 4D). Similarly, distance from parahippocampal area TF was more strongly correlated with areal G2 than with any of the univariate genetic principal components. Additionally, areal G2 showed stronger correlations with brain maps indexing laminar complexity of cortical myeloarchitecture (i.e. the first microstructural profile covariance gradient; $|r_s|= 0.89$) and cytoarchitecture (i.e. externopyramidization; $|r_s|= 0.72$) than did individual feature PCs (Extended Data Figure 4D). Overall, these results indicated that the integrative, multivariate MIND gradients G1 and G2 were more precisely aligned with the

anatomical predictions of dual origin theory than the genetic principal components of any of the individual, univariate structural MRI features that were used to estimate MIND.

Even if true, it still feels like a simplistic representation of complex data, which will likely fail to capture the complexity of the brain.

While any dimensionality reduction technique will definitionally lose complexity, we note that G1 and G2 capture >70% of genetic covariance across 276 edges, suggesting they represent major organizational principles rather than oversimplified representations. We do not claim they capture all complexity. We include the following discussion to emphasize these point:

Lines 125-133:
Given this evidence for distributed effects of localised genetic variation on similarity (or dis-similarity) of multiple pairs of cortical areas, we estimated the genetic correlations between all possible pairs of MIND edges, compiled as a {276 edge x 276 edge} genetic correlation matrix, *G* (Fig 2C). There were evidently many strong genetic correlations between edges in the network and we used gradient decomposition (Vos De Wael et al., 2020) to summarise genetic effects on MIND networks in terms of the first two gradients, G1 and G2 (Fig 2D). **Together, they accounted for over 70% of the (co)variance in the inter-areal genetic correlation matrix, *G* (see Methods for details), suggesting that they represent major organizational principles rather than oversimplified representations of the genetic architecture of the human cortex.** As the gradient decomposition was performed on an edge-by-edge matrix, each of the resulting gradients can also be considered as a network, with each edge weighted by its coefficients on G1 or G2.

<u>2.5:</u> The authors identify genetic overlap between G1 and G2 and psychiatric dimensions (some of which might be explained by BMI? [minor]), but this is not necessarily groundbreaking or novel (rG/MR between brain structure and psychiatric domains has been reported before). I wonder if you would pick up the same signal using the first 2 PCs from a PCA performed on all brain features? In short, I was not convinced of the added value of studying MIND phenotypes, due to their complexity and lack of interpretability. Maybe a comparison of results against other approaches that can reduce the dimension (PCA, ICA, network-based approaches…) could help make a stronger case for the use of MIND in genetics.

We break down our responses to this important point into multiple parts for clarity.

The authors identify genetic overlap between G1 and G2 and psychiatric dimensions (some of which might be explained by BMI? [minor]).

We agree with the Reviewer's comment that the reverse causal relationships observed for G2 (BMI → G2, CRP → G2) suggest that this gradient may partially reflect downstream consequences of metabolic or immune processes. This is an important finding indicating that immunometabolic health may influence cortical architecture along archicortical trends. We have adapted the text to clarify these results and avoid overstating causality:

Line 375-382:

Subsequent MR analysis demonstrated no forward causal effect of G2 on any disorders or traits (Extended Data Table 1). However, there was strong evidence for a reverse causal effect of BMI on G2 (MR-APSS β=-0.15, P = 8.0e-04 uncorrected) and suggestive evidence for a reverse causal effect of CRP on G2 (MR-APSS β=-0.07, P = 0.045 uncorrected), **indicating that immuno-metabolic health may have downstream effects on archicortically aligned trends of cortical architecture.** These results provide preliminary support for the prior prediction arising from dual origin theory (Pandya et al., 2015) that the two trends of cortical differentiation are differentially implicated in pathogenesis of neuropsychiatric disorders.

[The authors identify genetic overlap between G1 and G2 and psychiatric dimensions], but this is not necessarily groundbreaking or novel (rG/MR between brain structure and psychiatric domains has been reported before). I wonder if you would pick up the same signal using the first 2 PCs from a PCA performed on all brain features? In short, I was not convinced of the added value of studying MIND phenotypes, due to their complexity and lack of interpretability. Maybe a comparison of results against other approaches that can reduce the dimension (PCA, ICA, network-based approaches…) could help make a stronger case for the use of MIND in genetics.

First, we note that MIND network phenotypes are already being widely used to study brain network alterations across neuropsychiatric disorders and in many other contexts. A comprehensive analysis of the extent of genetic overlap between MIND and these traits therefore intrinsically presents an important contribution to inform the current research landscape. We highlight this point with the following text in the introduction.

Lines 42-48:

Finally, since its recent development, MIND has been rapidly taken up by the research community for the analysis of brain network organization across many different applications, including lifespan development (Liang et al, 2025), neuropsychiatric and neurodegenerative disorders (Estevez-Fraga et al, 2025; García-San-Martín et al, 2025; Zou et al, 2025; Montagnese et al, 2025; Ge et al, 2025), cross-species connectomics (Hutchings et al, 2025; Smith et al, 2025), and beyond. As such, a comprehensive genetic analysis of MIND network phenotypes is not only empirically and theoretically justified, but also timely.

However, we appreciate the reviewer's suggestion to confirm that the observed genetic overlaps between MIND gradients with clinical traits could not be exclusively explained by these network phenotypes being proxies of simpler measures of brain structure. To do so, we calculated the genetic correlations between MIND gradients and the univariate measures of brain structure which are included in MIND network construction as well as surface area. We reasoned that if MIND gradients represented a novel, emergent phenotype of cortical architecture, they should not be too strongly indicated by a single, simpler index of global brain structure. These analyses and their resulting plots and discussion are reported in Extended Data Figure 4B and the main text as follows:

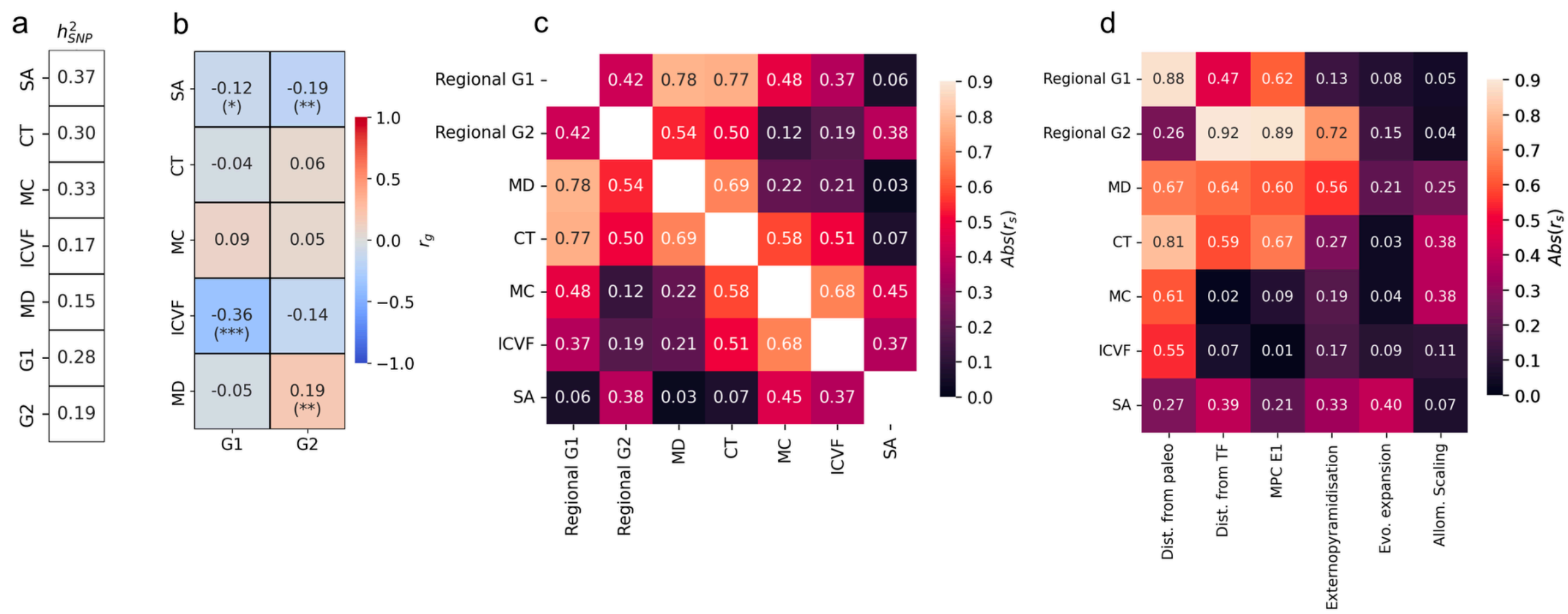

Extended Data Figure 4 **Genetic and spatial relationships between MIND gradients and univariate global measures of brain structure.** A) The SNP heritabilities of MIND G1/G2 and global measures of brain structure. MIND phenotype heritabilities were calculated using LDSC, and all other heritabilities were as reported in Warrier et al (2023). SA: surface area, ICVF: intracellular volume fraction, CT: cortical thickness, MC: mean curvature, MD: mean diffusivity. B) The genetic correlation (LDSC) between MIND G1/G2 and global measures of brain structure, using publicly-available summary statistics from Warrier et al (2023). P-values were FDR corrected: $* < 0.01 < P < 0.05$, $**0.001 < P < 0.01$, $***P < 0.001$. C) The spatial relationships (absolute Spearman correlation) between MIND G1 and G2 regional scores and the first principal components of regional genetic similarity matrices SA, CT, MC, ICVF, and MD, from Warrier et al (2023). D) The spatial relationships (absolute Spearman correlation) of MIND G1 and G2 regional scores, and the principal genetic components from part (C), with six cortical maps indexing complementary aspects of dual origin theory, brain evolution, microstructure, and areal scaling. These include (1) the distance from paleocortex and (2) distance from area TF (the two dual-origin geodesic distance maps discussed in Fig. 3), (3) the principal gradient of the T1w/T2w microstructural profile covariance network (MPC E1), reflecting laminar myeloarchitectural complexity (Paquola et al, 2020), (4) cortical externopyramidisation, a measure of laminar cytoarchitectural differentiation (Amunts et al, 2013; Sydnor et al, 2021), (5) evolutionary expansion (Sydnor et al, 2021), and (6) allometric scaling (Sydnor et al, 2021).

Lines 159-168:

We next studied the extent to which MIND gradients G1 and G2 were genetically related to other commonly-studied measures of whole-cortex structure. As shown in Extended Data Figure 4A, genetic correlations between G1/G2 and whole-brain CT, MD, ICVF, MC, and surface area (SA) (Warrier et al, 2023) were generally weak-moderate ($0.04 < |r_g| < 0.36$ for all comparisons), although four of these comparisons were statistically significant (G1-SA r_g = –0.12; ICVF-G1 r_g = –0.36; G2-SA r_g = –0.19; G2-MD r_g = 0.19, all $P_{FDR} < 0.05$). Of note, the weak genetic correlations between global surface area and both G1 and G2 indicated that MIND gradients were not simply attributable to genetically determined allometric scaling of cortex. Indeed the generally low magnitude of these genetic correlations was not sufficient to explain the genetic architecture of multivariate MIND gradients in terms of the genetics of univariate MRI phenotypes.

Regarding alternative dimensionality approaches: structural similarity provides an interpretable bridge between structural MRI features defined locally (e.g. cortical thickness) and network-based measures that are defined relationally between brain areas (e.g. functional connectivity). Crucially, alternative methods to reduce the dimensionality of brain structure

cannot play this role – for example, a PCA on all structural features would yield weights per brain region and per feature, without any natural way to study interregional relationships in any theoretically-justified or interpretable manner.

Therefore, the interpretation and impact of the genetic overlaps we observe between MIND and clinical traits provide a novel link between the genetics of brain structure with directly-comparable measures of functional connectivity as well as relevant biomedical outcomes. Such integrated insights were not possible in previous imaging-genetics research studying brain structure and functional connectivity separately, nor would they be possible through the application of other dimensionality reduction approaches such as PCA.

We underscore this point in the text as follows:

Lines 27-34:

It has already been established that the similarity between cortical areas which is measurable at the whole-brain scale of MRI, e.g., in terms of inter-areal correlation of macro- and/or micro-structural MRI feature vectors, is representative of the architectonic similarity between areas at a cellular scale (Seidlitz et al, 2018; Paquola et al, 2019; Sebenius et al, 2023, 2024). Architectonically similar areas of cortex are also more likely to be inter-connected by axonal projections (Beul et al, 2015, 2017; Wei et al, 2018). As such, structural similarity is uniquely positioned to bridge structural MRI features defined locally (e.g. cortical thickness) with network-based measures that are defined relationally between brain areas (e.g. functional connectivity).

Lines 333-339:

Taken together, these results position MIND as a novel and useful bridge between genetics, univariate brain structure, and functional connectivity: MIND being a structurally-derived relational measure that is substantially more heritable than the functionally-derived relational measure of FC, while also being robustly and concordantly correlated with FC genetically.

To offer further support for this claim, we confirm that the strong genetic relationships observed between MIND and FC could not be explained by genetic correlations with univariate measures of global brain structure alone. The results of this confirmatory analysis are shown in Extended Data Figure 6B and discussed as follows:

Extended Data Figure 6B:

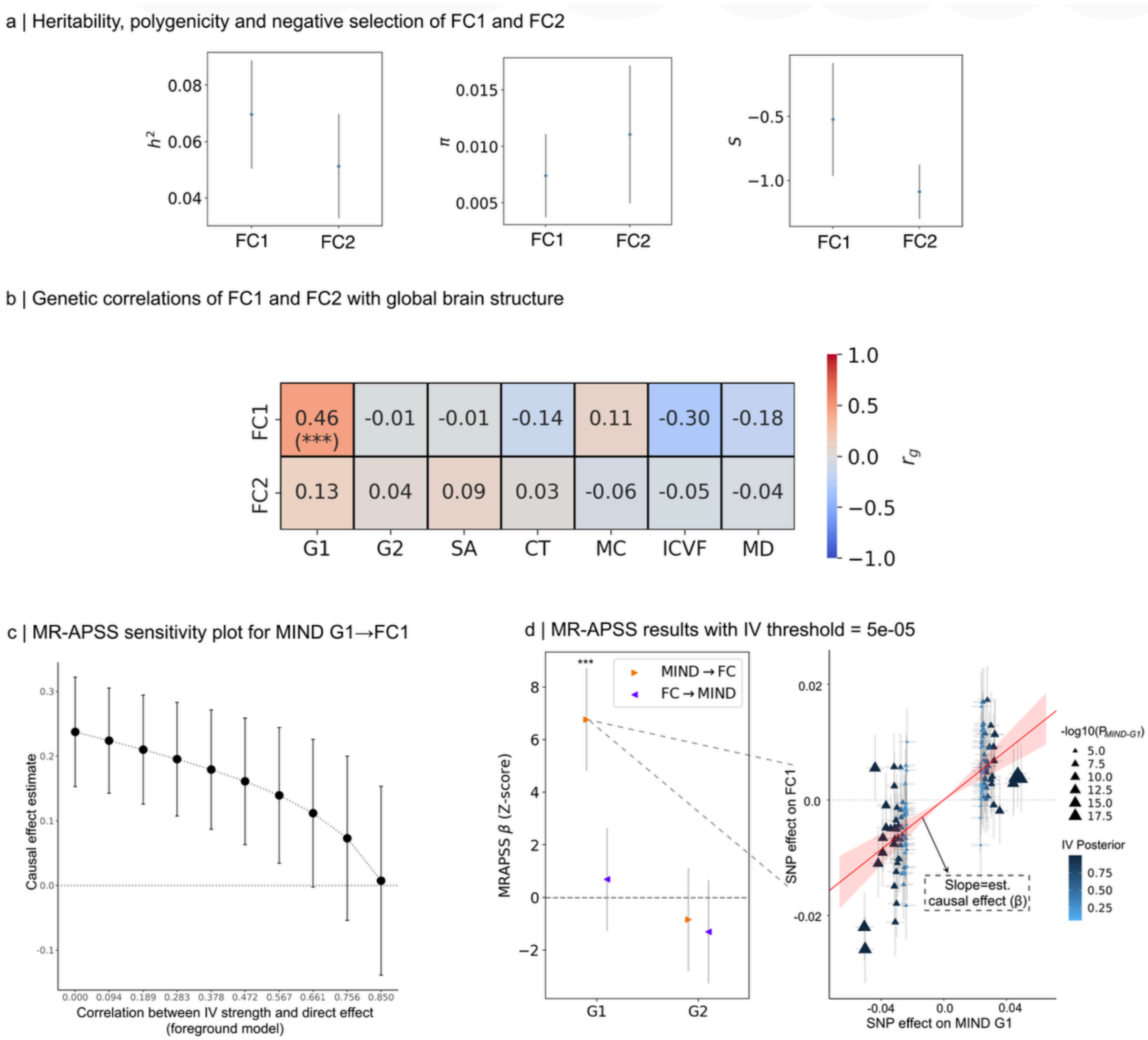

Extended Data Figure 6 Genetic relationships between FC, global brain structure, MIND gradients. A) Estimates of SNP-based heritability, polygenicity parameter π, and negative selection parameter S of fMRI-derived functional connectivity (FC) summarised along the two principal MIND genetic gradients (labeled FC1 and FC2). All estimates were calculated using SBayesS (Zeng et al, 2018). Shaded lines indicated 95% confidence intervals. B) The genetic correlation (LDSC) between FC1 and FC2 with MIND G1, MIND G2, and five univariate global measures of brain structure, using publicly-available summary statistics from Warrier et al (2023). SA: surface area, ICVF: intracellular volume fraction, CT: cortical thickness, MC: mean curvature, MD: mean diffusivity. P-values were FDR corrected: $* < 0.01 < P < 0.05$, $** 0.001 < P < 0.01$, $*** P < 0.001$. C) Sensitivity plot for the result shown in Figure 4E showing a significant causal estimate of MIND G1 on FC1. This plot tests the robustness of the result to model assumptions about the relationship between instrumental variable (IV) strength and the direct causal effect in the foreground model, which is assumed to be zero (Hu et al, 2022). The plot shows here that the positive causal effect of MIND on FC along G1 result remains significant as long as this value remains below 0.661. To put this number in context, the result remains significant for over twice as large a parameter space as the causal evidence for body mass index (BMI) on Type II diabetes, a widely-accepted causal relationship which was previously shown to be robust for values below 0.247 (Hu et al, 2022). D) A replication of the gradient-level MR-APSS results shown in Figure 4E, but with an IV threshold of $5e-05$ instead of $5e-04$. The left plot shows estimated (forward and reverse) causal relationships between MIND and FC (with 95% CI) separately for both G1 and G2. The right plot shows the highly significant causal effect of G1 on FC1, which is shown by the significantly positive slope of the regression plot (Z-scored β=6.2, $P = 6.0$e-10).

Lines 309-312:

The genetic relationship between MIND and FC was clarified and strengthened through gradient-level comparison. On the paleocortical trend represented by G1, MIND G1 scores and FC1 scores were significantly genetically correlated at approximately the same level as the most strongly genetically correlated edges (HDL r_g = 0.44, P = 1.2e-04; LDSC r_g = 0.46, P = 6.5e-08). **FC1 was not significantly genetically correlated with univariate global measures of brain structure (CT, MC, ICVF, MD, or SA; Extended Data Fig. 6B), suggesting that the genetic overlap between MIND G1 and FC1 reflected a shared network organization rather than associations with simpler univariate structural metrics.** However, on the archicortical trend represented by G2, there was no significant genetic correlation between MIND G2 and FC2 scores (HDL r_g = 0.04, P = 0.61; LDSC r_g = 0.05, P = 0.76; Fig. 4D). This result suggests that the two genetically independent trends of cortical architecture, defined by structural MRI network analysis and aligned with dual origin theory based on cortical histology, have quite distinct relationships to brain functional activity.

Dear Reviewers,

Thank you very much for your feedback on our manuscript, “The genetic architecture of cortical similarity networks.” We are delighted that our initial revisions have addressed the majority of your initial comments and concerns. We have now included further clarifications to the text in the response to the remaining suggestions. As before, the remainder of the response is structured as follows:

• In black font, we restate each reviewer comment.

• In purple font, we offer a brief response and provide a description of the changes made to the manuscript to address the comment.

• In red font, we include the verbatim changes to the manuscript itself. Where applicable, **bold-faced** red font is used to highlight changes within an existing passage.

We greatly appreciate your continued engagement with our work.

Yours sincerely,
Isaac Sebenius on behalf of the authors

Reviewer #1 (Remarks to the Author):

The revised manuscript has addressed many of my concerns and showcased the unique aspects of the MIND they derived from multi-modal imaging.

We appreciate this comment and are glad that our initial revision addressed the majority of this reviewer's concerns.

There are two remaining issues I hope the authors can improve upon:
1). Although the authors has used MR to test the potential causal relationships between their derived metrics and psychiatric disorders, it is by no means to be actually causal. This is especially true since the significance test results are marginal at best. I recommend to tone down the causal language and discuss the limitations.

We thank the reviewer for this comment. We have softened the language surrounding the causality between MIND phenotypes and psychiatric disorders (particularly for G1 on schizophrenia) and discussed the need to replicate these results as follows:

Lines 375-379:

"Follow up analysis **provided tentative evidence** for a forward (but not reverse) causal effect of G1 on schizophrenia (G1→SCZ, MR-APSS $\varepsilon = 0.037$, $P = 0.04$ uncorrected; SCZ→G1, MR-APSS ε=-0.004, $P = 0.90$ uncorrected; Supplementary Table A1), **consistent with the possibility that paleocortical architecture may contribute to schizophrenia risk."**

Lines 388-391:

"These results provide preliminary support for the prior prediction arising from dual origin theory (Pandya et al, 2015) that the two trends of cortical architectonic differentiation may be differentially implicated in pathogenesis of neuropsychiatric disorders, **though replication of the putative causal links between them represents an important direction for future work**."

2). It is good to see there are more comparisons to traditional uni-modal metrics to show MIND cannot be fully explained by one single modality or allometric scaling in the polygenic context. Yet it is still not addressing them in the loci level. This is in the same vein with the comparison to genetic map by Chen et al., whereas method differences cannot fully account for the uniqueness of the end results. Please put the discussion in context.

We have included the following discussion to address these important points as follows:

Lines 155-166:

"We next studied the extent to which MIND gradients G1 and G2 were genetically related to other commonly-studied measures of whole-cortex structure. As shown in Supplementary Figure 4B, genetic correlations between G1/G2 and whole-brain CT, MD, ICVF, MC, and surface area (SA) (Warrier et al, 2023) were generally weak-moderate ($0.04 < |rg| < 0.36$ for all comparisons), although four of these comparisons were statistically significant (G1-SA rg = −0.12; ICVF-G1 rg = −0.36; G2-SA rg = −0.19; G2-MD rg = 0.19, all PFDR < 0.05). Of note, the weak genetic correlations between global surface area and both G1 and G2 indicated that MIND gradients were not simply attributable to genetically determined allometric scaling of cortex. **Some degree of overlap with prior structural imaging GWAS is nonetheless expected, and is reflected both in these global correlations and at the level of individual loci - for example, *rs798511* (Fig. 2B) has previously been linked to ventricular size (Räsänen et al, 2024).** However, the generally low magnitude of these genetic correlations suggested that this overlap is partial, and that the genetic architecture of multivariate MIND gradients is not predominantly captured by the genetics of univariate MRI phenotypes."

Line 254 - 262:

"Overall these results support our prediction that the two principal gradients of genetically coordinated effects on MIND similarity networks should be anatomically aligned with the paleocortical (especially) and archicortical trends of dual origin theory (Goulas et al, 2019; Valk et al, 2020, 2022). As such, they show how dual origin theory can provide a framework for describing and genetically explaining two major trends of cortical differentiation in the human brain, in addition to its original focus on evolutionary progression of cortical structure across species. **More broadly, these results suggest that the dual origin framework may serve as a unifying interpretation for other cortical trends that have already been identified in previous neuroimaging and genetics studies (Chen et al, 2013; Warrier et al, 2023). This possibility warrants systematic investigation in future work.**"

Reviewer #2 (Remarks to the Author):

Thank you for your revisions. I think some of them help make a better case about the MIND phenotypes and the findings.
I don't have more comments that would really improve the manuscript without wasting everyone's time in another round of review.

We greatly appreciate the positive evaluation of the revised manuscript.
